# New 4-(Morpholin-4-Yl)-3-Nitrobenzhydrazide Based Scaffold: Synthesis, Structural Insights, and Biological Evaluation

**DOI:** 10.3390/molecules30163343

**Published:** 2025-08-11

**Authors:** Michał Janowski, Sara Janowska, Sylwia Andrzejczuk, Urszula Kosikowska, Radomir Jasiński, Barbara Mirosław, Marcin Feldo, Monika Wujec, Oleg M. Demchuk

**Affiliations:** 1 Medical University of Lublin, Doctoral School, 7 Chodzki Str., 20-093 Lublin, Poland; jjjmichal1@gmail.com; 2 Department of Pathobiochemistry and Interdisciplinary Applications of Ion Chromatography, Biomedical Sciences, Medical University of Lublin, 1 Chodzki Str., 20-093 Lublin, Poland; sara.janowska@wp.pl; 3 Medical University of Lublin, Department of Pharmaceutical Microbiology, 1 Chodzki Str., 20-093 Lublin, Poland; sylwia.andrzejczuk@umlub.pl (S.A.); urszula.kosikowska@umlub.pl (U.K.); 4 Cracow University of Technology, Department of Organic Chemistry and Technology, Warszawska 24, 31-155 Cracow, Poland; 5 Maria Curie-Sklodowska University in Lublin, Department of General and Coordination Chemistry and Crystallography, Institute of Chemical Sciences, Faculty of Chemistry, Maria Curie-Sklodowska sq. 2, 20-031 Lublin, Poland; barbara.miroslaw@mail.umcs.pl; 6 Medical University of Lublin, Chair and Department of Vascular Surgery and Angiology, 11 Staszica St., 20-081 Lublin, Poland; marcin.feldo@umlub.pl; 7 Medical University of Lublin, Department of Organic Chemistry, 4a Chodzki Str., 20-093 Lublin, Poland; monika.wujec@umlub.pl; 8 The John Paul II Catholic University of Lublin, Faculty of Medicine, Konstantynów 1J, 20-708 Lublin, Poland

**Keywords:** semicarbazides, thiosemicarbazides, hydrazones, hydrazides, antibacterial activity

## Abstract

The search for new antimicrobial agents is one of the major challenges in contemporary medicinal chemistry due to the global issue of increasing drug resistance. In our efforts to identify chemical structures with antibiotic activity that differ from commonly used antibiotics, we focused our research on (thio)semicarbazides and hydrazones. Guided by literature reports, we designed and synthesized a series of novel semicarbazides, thiosemicarbazides, and hydrazones based on the structure of 4-(morpholino-4-yl)-3-nitrobenzohydrazide. The obtained derivatives were subsequently evaluated in in vitro assays for their activity against reference strains of Gram-positive and Gram-negative bacteria. Among the studied groups of compounds, the semicarbazide derivatives exhibited the highest activity. The most active compound identified in the study was a semicarbazide containing a 4-bromophenyl moiety. This compound showed antibacterial potential against Enterococcus faecalis, with a MIC value of 3.91 µg/mL. Among the thiosemicarbazides, the most active compound contained a 4-trifluoromethylphenyl group, with MIC values against Gram-positive bacterial strains (excluding Staphylococcus aureus) ranging from 31.25 to 62.5 µg/mL. None of the tested hydrazones exhibited antimicrobial activity against the examined bacteria. Additionally, the structures of the new compounds were confirmed by single-crystal X-ray analysis, which enabled the investigation of their properties using advanced quantum chemical calculations.

## 1. Introduction

The greatest challenge of contemporary research on new antimicrobial drugs is the growing multidrug resistance of pathogenic microorganisms [1,2]. The development of resistance to chemical substances is a natural defense mechanism of bacteria. Microorganisms resistant to antibiotic compounds existed long before antibiotics were introduced into medicine. Natural antibiotics, e.g., those produced by microorganisms living in soil, water, and plants [3], acted as chemical weapons in the evolutionary struggle for survival. In defense against them, bacteria have developed numerous resistance mechanisms, such as pumps that remove toxins and enzymes that break down chemical substances. These strategies existed long before the era of antibiotic therapy, proving that resistance is a natural and age-old phenomenon [4].

The fight of microorganisms against antibiotics has been going on for thousands of years and will not cease regardless of medical progress. The increase in resistance of microorganisms may be attributed to factors such as the irrational use of antibiotic therapy or the creation of large population centers [1,5]. Microorganisms demonstrate an extraordinary ability to acquire and spread antibiotic resistance through horizontal and vertical gene transfer mechanisms [4], and evolutionarily through spontaneous mutations, gene amplification, overexpression, etc. Therefore, the protection of public health requires constant research on new antibiotics [6].

In response to the described problem of drug resistance, many research groups are undertaking work aimed at improving the antibacterial effectiveness of known drugs [7]. However, such an approach is associated with the risk of a situation in which bacteria that have developed resistance to one drug will also be resistant to a new antibiotic with a similar structure or mechanism of action. Designing structurally new drugs that differ in terms of both chemical and biological mechanisms of action is a direction that limits the risk of cross-resistance to a new pharmaceutical. Therefore, the main goal of research on new antibiotics is to find new chemical structures, and not to continue searching for new members of already known chemical classes [8,9,10].

**Figure 1:**
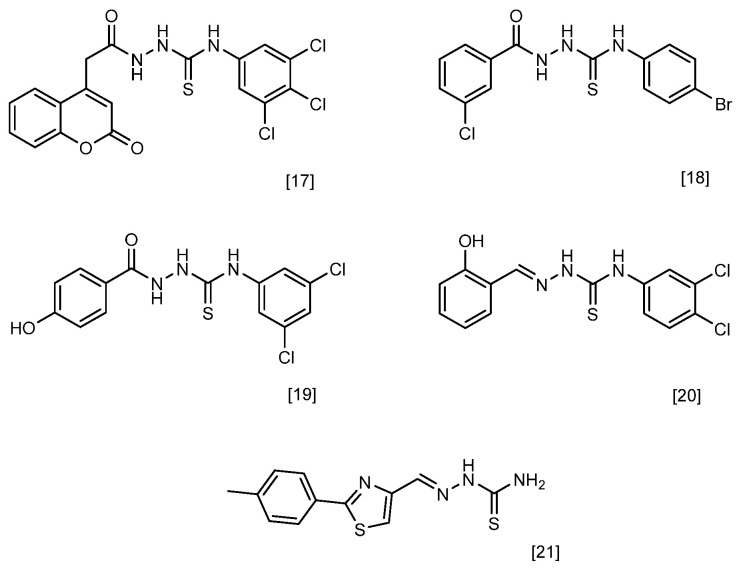
Chemical structures of the most active compounds reported in the cited literature, showing high antibacterial activity, including against *Staphylococcus aureus*.

One of the promising classes of compounds that allows for a wide range of chemical modifications is the semicarbazides [11]. Their structure (Figure 1) gives them a wide spectrum of biological and chemical properties. The structure of c semicarbazides differs from known antibacterial drugs as well as naturally derived antimicrobial compounds and therefore may constitute a new class of antimicrobial agents [12]. The analogues of semicarbazides are thiosemicarbazides. Many thiosemicarbazides with antimicrobial potential have been described [13]. Studies have shown that compounds containing this group can exhibit antitubercular [14], antiviral [15], and antibacterial activity [16,17,18,19,20,21]. Both semicarbazides and thiosemicarbazides are considered derivatives of hydrazine. Another related group of hydrazine derivatives with well-documented antimicrobial activity is hydrazones. These compounds exhibit pharmacological properties, including antifungal, antibacterial, antiviral, antimalarial, cytostatic, anti-inflammatory, and antipyretic effects [22,23]. In recent years, numerous scientific studies have been published on the antibacterial activity of hydrazones belonging to the Schiff bases and possessing an azomethine group [24,25,26,27,28,29,30]. The motif of hydrazone can be found in the structures of some drugs used as oral antibacterial agents, such as nifuroxazide and thiacetazone [31].

**Scheme 1:**
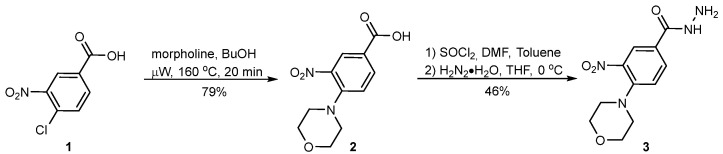
The synthesis of compound **3**.

In order to develop new antimicrobial compounds, we undertook research aimed at obtaining a series of derivatives of recently reported aromatic hydrazide 4-(morpholin-4-yl)-3-nitrobenzhydrazide **3** (Scheme 1), which shows intriguing physicochemical and biological properties [32,33].

## 2. Results and Discussion

### 2.1. Chemistry

The main aim of this study was to synthesize new derivatives of **3** to investigate their in vitro antimicrobial activity. The synthesis of **3** was based on microwave-assisted aromatic nucleophilic condensation of unprotected 4-chloro-3-nitrobenzoic acid (**1**), run in the presence of an excess of morpholine and butanol in a closed pressure vessel heated by microwave irradiation up to 160 °C for just 20 min. The purification of the product was as simple as acidifying the reaction mixture with diluted hydrochloric acid, followed by filtration and open-air drying of the pure product, which was obtained in 79% yield. Next, acid **2** was transformed to the corresponding chloride under the usual conditions and catalyzed by DMF reaction with SOCl_2_. Eventually, the last underwent a reaction with an excess of hydrazine hydrate. Upon the completion of the reaction, product **3** was isolated and purified by crystallization from chloroform in 46% yield. The main by-product formed in the reaction was 4-(morpholin-4-yl)-*N′*-[4-(morpholin-4-yl)-3-nitrobenzoyl]-3-nitrobenzohydrazide, formed as a result of the double acylation of hydrazine.

**Scheme 2:**
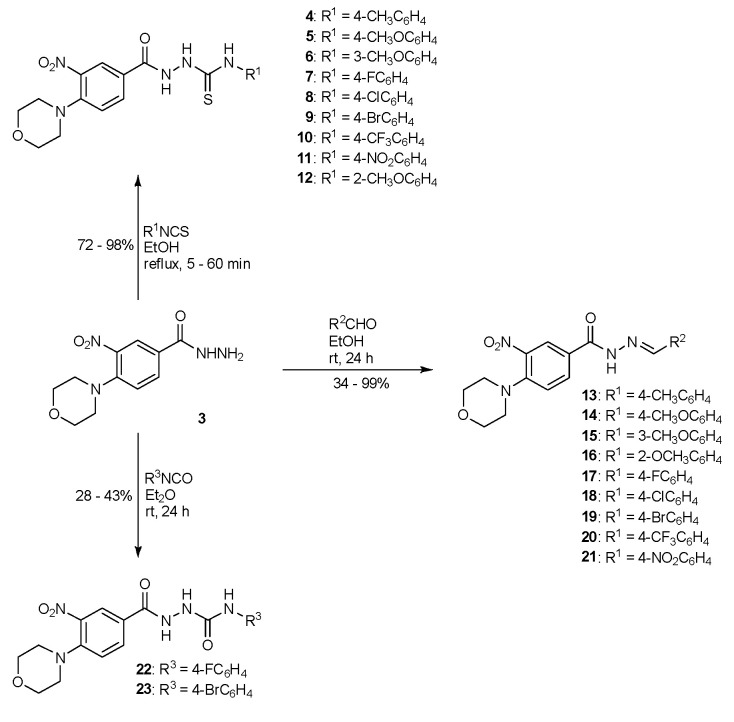
The synthesis of derivatives of compound **3**: thiosemicarbazides (**4**–**12**), semicarbazides (**22**, **23**), and hydrazones (**13**–**21**).

Compound **3** was tested against the panel reference Gram-positive and Gram-negative bacterial strains (Staphylococcus aureus, Staphylococcus epidermidis, Micrococcus luteus, Bacillus subtilis, Bacillus cereus, Klebsiella pneumoniae, Pseudomonas aeruginosa, Pseudomonas mirabilis, Acineobacter baumannii, Salmonella typhimurium) in vitro. It showed low activity with MIC over 1000 µg/mL, which could be easily rationalized by the fact that this compound has low affinity to proteins and cannot be considered a good ligand for biogenic metals. Nevertheless, in the case of Enterococcus faecalis (MIC = 250 µg/mL) and Escherichia coli (MIC = 62.5 µg/mL), some antimicrobial activity was observed, which may be attributed to the nitro group and hydrazine motive. Inspired by this observation, we decided to increase the antibiotic activity of **3** by preparing three series of its derivatives belonging to the compounds classed as biologically active: thiosemicarbazide, hydrazone, and semicarbazide. To synthesize new derivatives, 4-(morpholin-4-yl)-3-nitrobenzhydrazide (**3**) was used as a substrate in reactions with selected iso(thio)cyanates and corresponding benzaldehydes, as presented in Scheme 2.

New compounds within the series differed in the substituents attached to the terminal nitrogen atom of the hydrazide group. To determine the structures of the most promising functional groups attached to the maternal hydrazide **3,** thiosemicarbazide (**4**–**12**) and semicarbazide (**22**, **23**) derivatives were obtained. The biological activities of thiosemicarbazides and semicarbazides were compared with the activities of corresponding hydrazones (**13**–**21**) derived from the compound **3** in in vitro tests against a panel of Gram-negative and Gram-positive bacteria, using the double dilution method in broth.

The syntheses of thiosemicarbazides **4**–**12** were carried out by the reaction of 3 with isothiocyanates, according to the methodology previously described [12]. The different types of substituents in the phenyl group allow us to analyze the influence of electron-withdrawing/electron-donating functionalities on the antibacterial activities. The time required to obtain new derivatives was determined experimentally by monitoring the progress of the reaction by thin-layer chromatography. The reaction yields ranged from 72 to 98%. The structures of the new compounds were determined using ^1^H and ^13^C NMR spectra. The ^1^H NMR spectra showed chemical shifts of protons located on nitrogen atoms as three or two between 9.43 and 10.69 ppm.

**Figure 2:**
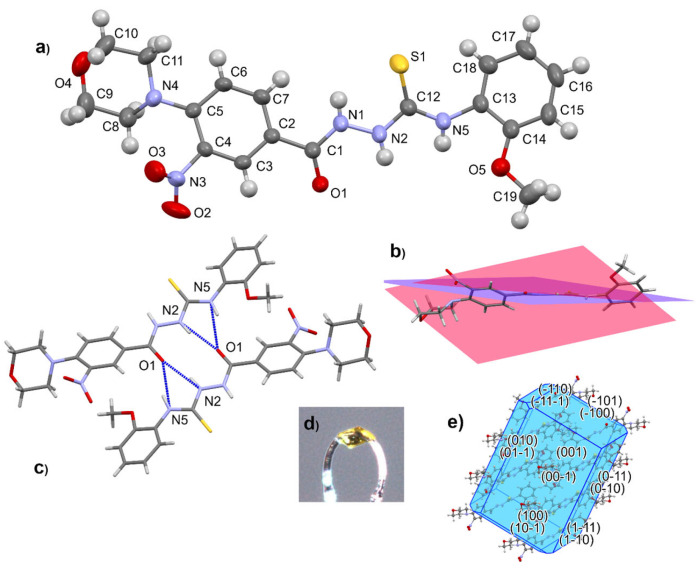
Molecular structure of **12** with atom numbering scheme (**a**), conformation of molecule (**b**), hydrogen-bonded dimer (**c**), crystal form (**d**), and morphology with crystal faces (**e**).

The molecular structure of a representative member of the thiosemicarbazide series was determined by single-crystal X-ray diffraction analysis of 4-(2-methoxyphenyl)-1-[4-(morpholin-4-yl)-3-nitrobenzoyl]thiosemicarbazide (**12**) (Figure 2).

Compound **12** crystallizes in the shape of yellow plates (space group *P*-1). The morpholine ring with chair conformation is attached by the N atom to the aromatic ring of 3-nitrobenzhydrazide. The thiourea part is not coplanar with the aromatic substituents. Geometry parameters are presented in the Supplementary Materials (Tables S1–S5, Figures S46 and S47). In the crystal structure of **12,** centrosymmetric hydrogen-bonded dimers are the main supramolecular entity.

The reaction between **3** and substituted benzaldehydes in anhydrous ethanol afforded hydrazones **13**–**21** in moderate to high yields (34–99%). The ^1^H NMR spectra of these compounds confirmed the proposed structures. In all the compounds, the NH peaks of hydrazone appeared at δ 11.75–12.16 ppm. The peak for =CH group was observed at δ 8.35–8.76 ppm, confirming the formation of final products.

**Figure 3:**
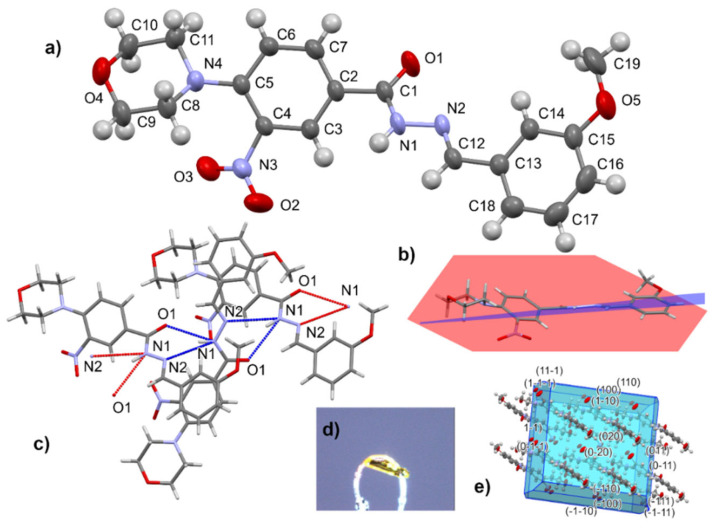
Ortep view on molecule **15** with atom numbering scheme (**a**), side view of the molecule (**b**), bifurcated N1-H1…O1 and N1-H1…N2 hydrogen bonds (**c**), crystal form (**d**), and morphology with Miller indices of main crystal faces (**e**).

X-ray analysis of hydrazones was performed on compound **15** as an example. It gave yellow needle-like crystals (Figure 3). The hydrazide part is not coplanar with the nitrophenyl fragment (twisted by ca. 39°). Worth mentioning is that nitro -NO_2_ and carbonyl C=O groups are on opposite sides, whereas in **12,** these groups were both on the same side of the molecule. The Supplementary Materials contain details on molecular and crystal structure (Tables S1–S5, Figures S48 and S49). The monoclinic crystal (space group *P*2_1_/*c*) is stabilized by intermolecular bifurcated N1–H1…O1 and N1–H1…N2 hydrogen bonds.

Eventually, semicarbazide derivatives **22**, **23** were obtained in the reaction of hydrazide with 4-fluorophenyl isocyanate and 4-bromophenyl isocyanate, respectively, in diethyl ether at room temperature. In the ^1^H NMR spectra signals of protons NH-NH-C(=O)-NH group as three singlets at 8.20 ppm, 8.86 ppm, and 10.35 ppm were observed.

**Figure 4:**
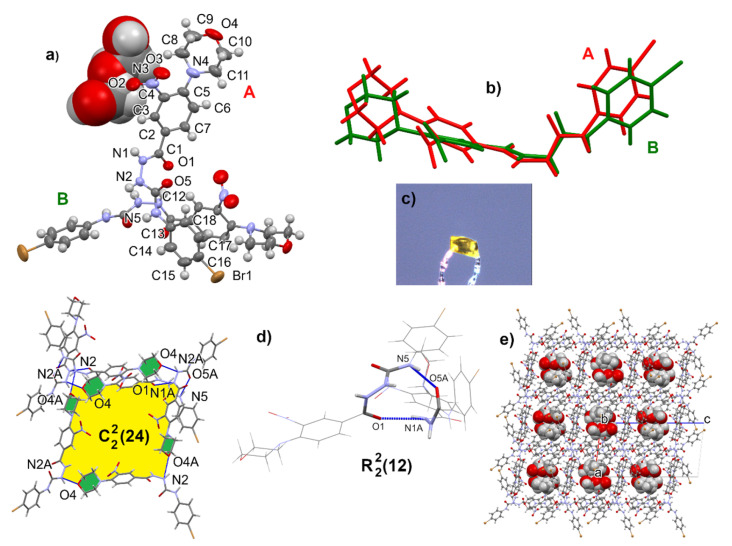
Ortep view on molecules A and B of compound **23** in a methanol solvate with atom numbering scheme given for molecule A (**a**), overlap of two symmetrically independent molecules (**b**), crystal form (**c**), hydrogen bonding motifs (**d**), and clathrate structure with methanol molecules inside (**e**).

The geometry of the semicarbazides was confirmed by XRD analysis of **23** crystallized from methanol. The crystals appeared on the second day, and the diffraction analysis showed the structure of a methanolic solvate. The crystal architecture is built of $R_{2}^{2}(12)$ rings based on two interactions: N5-H5…O5A and N1A-H1A…O1. The next units are linked by an analogous ring (N5A-H5A…O5 and N1-H1…O1A), resulting in a chain of rings. The morpholine O4 and O4A atoms are acceptors of additional N-H…O interactions, forming spiral $C_{2}^{2}(24)$ chains (Figure 4). In the asymmetric unit, there are two molecules of 4-(4-bromophenyl)-1-[4-(morpholin-4-yl)-3-nitrobenzoyl]semicarbazide and disordered over three positions, with methanol molecules closed in cages. Geometric details can be found in Supplementary Materials in Tables S1–S5, Figures S50 and S51.

### 2.2. Antibacterial Evaluation

The in vitro antibacterial activity of the synthesized compounds derived from 3 thiosemicarbazide, hydrazone, and semicarbazide was assessed based on the inhibition of the growth of reference bacterial strains in vitro, using the broth microdilution method. The results of antibacterial activity tests (MIC values) are presented in Table 1.

In the antibacterial activity tests, all nine hydrazones (**13**–**21**) did not show sufficient antibacterial activity against any of the bacterial strains used in the study. At the same time, eight out of nine tested thiosemicarbazide derivatives (**4**, **5**, **7**–**12**) showed moderate antibacterial activity. Notably, both obtained semicarbazide derivatives (**22**, **23**) showed a clear antibacterial activity, which in some cases exceeded the activity of the reference drugs used in the studies.

The predictable lack of activity of hydrazones **13**–**21** may be due to their lower ability to form strong hydrogen bonds with proteins, as they act as moderate hydrogen bond acceptors (Ar-C=O----H) and rather poor donors of a single hydrogen bond (Ar-C(O)NH----O) (Figure 4). This hypothesis is supported by the distribution of the electrostatic potential obtained in the results of the DFT calculations.

Since we were only able to perform XRD analysis of three compounds from those obtained—**12**, **15**, and **23**—which belong to three studied classes (hydrazones, thiosemicarbazides, and semicarbazides) but bearing different substituents at phenyl moiety, the equilibrium geometry of identically substituted compounds should be calculated to make a proper comparison of the electronic properties. The bromine substituent present in the most active compound **23** was therefore selected. To assure proper steric configuration of calculated compounds, DFT calculations of the equilibrium geometry of compounds **9**, **19**, and **23** were performed at the ωB97X-D/6-311g(d,p) level of theory, and the geometry of the obtained structures was compared with experimental XRD data. A satisfactory correlation was observed, as presented in Table 2.

The calculations confirmed that compound **23** has a higher potential to form hydrogen bonds than compound **9**, which in turn has a greater potential than compound **19**.

**Figure 5:**
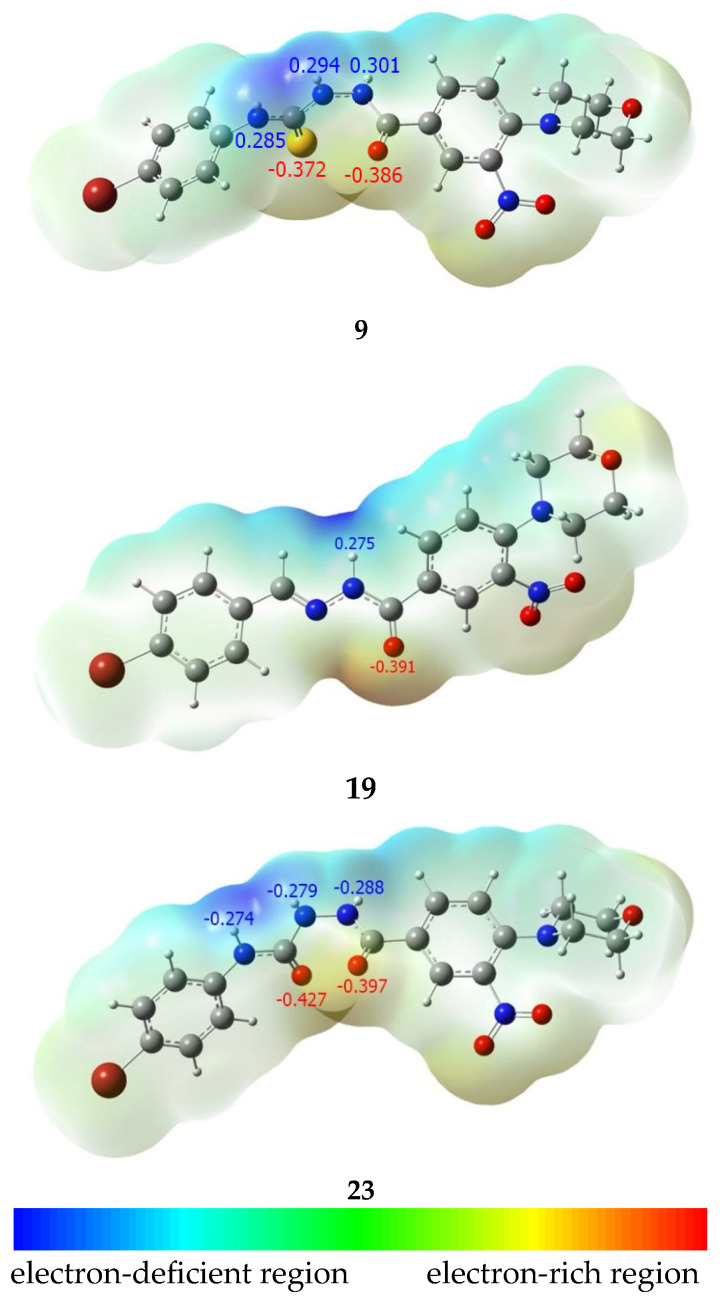
Molecular electrostatic potential (MEP) map coloured in the range of −0.17 to 0.17 hartree (−106.6 to 106.6 kcal/mol) for the spin density surface isovalue = 0.004, and Mulliken charges at hydrogen bond donor and acceptor atoms.igure.

The antibacterial activity present in molecules from the thiosemicarbazides may be related to their higher ability to chelate metals in coordination complexes. This is due to the fact that they contain non-bonding electron pairs at soft nucleophilic heteroatoms (N and S) and π electrons of >C=O and >C=S bonds. Consequently, they are characterized by a strong ability to form coordination interactions with metal atoms and ions [34]. Thiosemicarbazide derivatives have the ability to form complexes with iron, zinc, nickel, copper, and other metal ions that are important for biological processes and are present in some active sites of enzymes [13]. Nevertheless, the sulfur atom present in thiosemicarbazides **4**–**12** is a poor acceptor of a hydrogen bond. Compared to the sulfur atom present in compounds**,** the oxygen atom in the carbonyl moiety is a relatively stronger hydrogen bond acceptor (Figure 5). That is why in the cases of interactions with proteins, where coordination of the metal atom is not involved, thiosemicarbazides could not be especially active. The low activity of sulfur-containing derivatives (**4**–**12**) indicates that, in our cases, an interaction of tested compounds with metal ions is not responsible for the antimicrobial activity, but interactions with proteins still may be very important. Thus, hydrazones and thiosemicarbazides, from the series obtained by us, are characterized by low affinity to important molecular targets for antimicrobial activity, so they are not active. In contrast to that, semicarbazides **22** and **23** possess two strong hydrogen bond acceptors and three strong hydrogen bond donors. Thus, even in the cases where coordination of metal is not involved, they form strong complexes with proteins by the formation of multiple hydrogen bonds. This hydrogen bond-forming potential is clearly visible on the crystallographic structure of **23** (Figure 4), where two oxygen atoms are involved in the formation of the hydrogen bond, enforcing the crystal net. Additionally, in the model molecule **9**, the C=O bond is more polarized (charges on atoms C and O are equal 0.469e and −0.386e, respectively) than the C=S bond (charges on atoms C and S are 0.258e and −0.372e, respectively), while in the cases of the model molecule **23,** charges on atoms C and O are +0.462e and -0.397e (for hydrazide carbonyl) as well as +0.586e and −0.427e (for semicarbazide carbonyl), respectively. This makes semicarbazides more polar than thiosemicarbazides, and therefore more soluble in water. This increases the ability of semicarbazide molecules to migrate to the molecular target located in the cell, in vitro. All three types of compounds obtained possess additional functions as a nitro group (bioactive potential of the nitro group was recently evaluated [35,36]), a morpholine motif, and phenyl substituents. Those groups may be responsible for secondary interactions in the ligand–proteins complexes and the antimicrobial activity of the compounds.

The theoretical explanations presented above are supported by the following experimental data: thiosemicarbazide (**7**), containing a 4-fluorophenyl group, shows low bacteriostatic activity, within the MIC range from 500 µg/mL to >1000 µg/mL. Its analogue, semicarbazide (**22**), shows MIC values ranging from 7.81 µg/mL to >1000 µg/mL, showing higher activity against three bacterial strains (*Stapylococcus epidermidis* ATCC 12228, *Enterococcus faecalis* ATCC 29212, *Bacillus cereus* ATCC 10876) than the reference drug used (colistin). The corresponding hydrazone **17** was not active.

The highest potential was demonstrated by compound **23**, which is a semicarbazide containing a 4-bromophenyl moiety, characterized by antibacterial activity with a MIC value of 7.81 µg/mL against *S. epidermidis* ATCC 12228, with activity exceeding two reference drugs by almost two and three times: amoxicillin (MIC = 12.5 µg/mL) and colistin (MIC = 31.25 µg/mL). This compound also showed high activity against *E. faecalis* ATCC 29212, with a MIC value of 3.91 µg/mL (amoxicillin MIC = 0.39 µg/mL, colistin MIC = 100 µg/mL). Those values are much higher than we observed in the case of the sulfur analogue **9** (Table 1) and inactive **19**.

Most of the tested thiosemicarbazide derivatives showed low to moderate antibacterial activity. The antibacterial potential of these compounds was higher against Gram-positive bacteria (MIC from 31.25 µg/mL to >1000 µg/mL) than against Gram-negative bacteria (MIC from 500 µg/mL to >1000 µg/mL). The most sensitive bacterial strain to the action of compounds containing a thiourea group was *E. faecalis* ATCC 29212, the multiplication of which was inhibited by eight out of nine molecules from this group (MIC from 62.5 µg/mL to >1000 µg/mL).

The most active thiosemicarbazide derivative was compound **10**, which had a 4-trifluoromethylphenyl group in its structure. This molecule showed antibacterial potential, expressed by the MIC value of 31.25 µg/mL against *Micrococcus luteus* ATCC 10240 and *B. cereus* ATCC 10876 strains. At the same time, it showed MIC of 62.5 µg/mL against *S. epidermidis* ATCC 12228, *E. faecalis* ATCC 29212, and *Bacillus subtilis* ATCC 6633 strains. Bactericidal activity tests were also conducted for the studied compounds, determining the MBC values. Among all synthesized compounds, only compound **10** demonstrated bactericidal activity in the tests, with values of 125 µg/mL for *S. epidermidis* ATCC 12228, 62.5 µg/mL for *E. faecalis* ATCC 29212, and 250 µg/mL for *Micrococcus luteus* ATCC 10240. These results are consistent with the previous work on the antibacterial activity of thiosemicarbazide derivatives, in which it was observed that the trifluoromethylphenyl fragment is an interesting pharmacophore, positively influencing the antibacterial activity in this group of compounds [12].

Analysis of data from other researchers suggests that the biological activity of thiosemicarbazide and semicarbazide derivatives increases upon the introduction of a phenyl moiety bearing a halogen substituent [17,18,19,20,21]. This relationship is also confirmed by the results of the compound series described in the present study. According to the cited literature, the highest activity was observed for compounds containing dichlorophenyl or trichlorophenyl groups with MIC values for the most active dichlorinated derivatives ranging from 1.95 to 15.63 µg/mL [19,20], while for trichlorinated derivatives, the values were significantly lower, ranging from 0.06250 to 0.00781 µg/mL [17].

Therefore, the inclusion of halogen substituents, especially trichlorinated ones, appears to be a reasonable strategy in the design of future compound series.

It is also noteworthy that a series of compounds containing a coumarin moiety described in the literature exhibited very high activity, including activity against *Staphylococcus aureus*, with MIC values for the most active compounds also in the range of 0.06250 to 0.00781 µg/mL [17].

When comparing these results with the findings of the present study, it can be concluded that the presence of a morpholine substituent does not enhance the biological activity within this class of compounds.

Consequently, as a future research direction, it seems valuable to consider the synthesis of thiosemicarbazide and semicarbazide derivatives bearing condensed heterocyclic systems, such as coumarin, which may significantly improve the biological properties of the obtained compounds.

## 3. Materials and Methods

### 3.1. Chemicals and Instruments

All the chemicals utilized in this research were obtained from Sigma-Aldrich (Munich, Germany) and employed without further purification. The ^1^H NMR, ^13^C NMR, and ^19^F NMR spectra were acquired on a JEOL ECZL500 (Jeol Ltd., 1-2, Musashino 3-Chome Akishima, Tokyo, Japan) in DMSO-d_6_. The IR spectra of the obtained compounds were recorded on a Nicolet 6700 FT-IR spectrophotometer. The NMR and IR spectra can be found in the Supplementary Materials. Melting points were measured with a Cole-Parmer Stuart SMP50 automatic melting point apparatus (Cole-Parmer Ltd., Staffordshire, United Kingdom) and were uncorrected. The purity of the compounds and the reaction progress were tracked by TLC using an aluminum sheet with 60 F_254_ plates (Merck Co., Kenilworth, NJ, USA) with a CHCl_3_/EtOH (10:1, *v*/*v*) solvent system. The microwave-assisted reactions were conducted within the SEM Discover Microwave Synthesizer (CEM Corporation, 3100 Smith Farm Road, Matthews, NC, USA). The HPLC and MS analyses were performed on a Shimadzu LCMS Q-Tof 9030 ESI (Kyoto, Japan) instrument equipped with a column Dr. Maisch (Dr. Maisch, Ammerbuch-Entringen, Germany) ReproSil-Pur Basic-C18 3 µm 100 mm, which was eluted at 40 °C by MeOH/H_2_O = 70/30 with a rate of 0.3 mL/min. The injections of analyte were applied at the first minute of elution.

#### 3.1.1. Synthesis of Compound **3**

**4-(morpholin-4-yl)-3-nitrobenzoic acid** (**2**)

The high-pressure microwave glass reactor equipped with a magnetic stir bar was charged with 40 mmol of 4-chloro-3-nitrobenzoic acid (**1**), 92 mmol of morpholine, 8 mL of butanol, and sealed with a Teflon-coated silicone cap. The reaction mixture was heated by microwave irradiation to achieve a temperature of 160 °C and stirred for an additional 20 min at this temperature. After that, the reactor was cooled down to ambient temperature, and the reaction mixture was poured into the solution of 50 mL saturated hydrochloric acid in 300 mL of water. The completion of precipitation of the product was promoted by dissolution in a mixture of 25 g of NaCl. The solid was filtered off, washed with water, and dried in air to yield 7.9 g of **2**. The compound was used without additional purification.

Yield: 79%. M.p.: 176 °C. The spectral data and properties of the obtained compound **2** were in full agreement with the literature data [37]

**4-(morpholin-4-yl)-3-nitrobenzhydrazide (3)**

The reaction flask equipped with a reflux condenser and magnetic stirring was charged with 20 mmole of 2, 30 mL of benzene, 30 mL of toluene, and 5 mL of SOCl_2_. The stirred reaction mixture was maintained in reflux conditions for 16 h. After the reaction completion, it was cooled down to ambient temperature, and solvents were evaporated off under reduced pressure. The residue was dissolved in 50 mL of dry THF. Such a prepared mixture was added dropwise to the stirred solution of 10 mL of 80% aqueous hydrazine in 150 mL of THF at −5 °C. After the completion of the addition, the stirred solution was allowed to heat up to ambient temperature, and the stirring was continued for an additional 16 h. The THF was evaporated off under reduced pressure, and the dispersed residue was washed with 100 mL of saturated sodium hydrogen carbonate, 100 mL of water, and 100 mL of diethyl ether. The crude product was dried in air and crystallized from chloroform to yield 2.4 g of pure **3**.

Yield: 46%. M.p.: 132 °C (decompose). IR (cm^−1^): 3300 (NH), 2831 (CH aliph.), 1610 (C=O), 1231 (C-O-C). ^1^H NMR (500 MHz, DMSO-d_6_) δ (ppm): 3.03–3.05 (m, 4H, 2CH_2_), 3.64–3.66 (m, 4H, 2CH_2_), 4.47 (s, 2H, NH_2_), 7.29 (d, 1H, Ar, *J* = 8.8 Hz); 7.97 (dd, 1H, Ar, *J* = 8.8, 2.2); 8.24 (d, 1H, Ar, *J* = 2.2), 9.83 (s, 1H, NH). ^13^C NMR (125 MHz, DMSO-d_6_) δ (ppm): 51.23, 66.38, 120.85, 125.53, 125.62, 132.60, 140.78, 147.29, 164.25. HRMS (ESI): *m*/*z* = calcd. 267.10878 (M + H+, C11 H14 N4 O4), found. 267.10814, diff. 2.401 ppm.

#### 3.1.2. Synthesis of Thiosemicarbazide Derivatives (**4**–**12**)

The 0.001 mole of 4-(morpholin-4-yl)-3-nitrobenzhydrazide was dissolved under heating in 5 mL anhydrous ethanol. An equimolar amount of an appropriate isothiocyanate was added to the solution. The mixture was refluxed for 5 min for compounds **4**, **5**, and **8**, for 10 min for compound **8**, and for 1 h for compounds **7**, **10**, and **12**. The solution was then cooled to room temperature, and the precipitate was filtered. The product was washed with hot water and diethyl ether. After drying, the product was crystallized from 96% ethanol.

**4-(4-methylphenyl)-1-[4-(morpholin-4-yl)-3-nitrobenzoyl]thiosemicarbazide (4)**

Yield: 98%. M.p.: 205 °C. IR (cm^−1^): 3218 (NH), 2945 (CH aliph.), 1639 (C=O), 1559 (CH arom.), 1351 (C=S), 1281 (C-O-C). ^1^H NMR (500 MHz, DMSO-d_6_) δ (ppm): 2.24 (s, 3H, CH_3_), 3.07 (m, 4H, 2CH_2_), 3.65 (m, 4H, 2CH_2_), 7.09 (d, 2H, Ar-H, *J* = 8.0 Hz), 7.23–7.30 (m, 2H, Ar-H), 7.34 (d, 1H, Ar-H, *J* = 8.8 Hz), 8.05 (dd, 1H, Ar-H, *J* = 8.8, 1.4 Hz), 8.37 (d, 1H, Ar-H, *J* = 1.3 Hz), 9.61 (s, 1H, NH), 9.68 (s, 1H, NH), 10.57 (s, 1H, NH). ^13^C NMR (125 MHz, DMSO-d_6_) δ (ppm): 21.08, 51.18, 66.34, 110.86, 126.78, 128.20, 137.02, 137.19, 140.35, 147.72. HRMS (ESI): *m*/*z* = calcd. 416.13870 (M + H+, C19 H21 N5 O4 S), found. 416.13867, diff. 0.076 ppm.

**4-(4-methoxyphenyl)-1-[4-(morpholin-4-yl)-3-nitrobenzoyl]thiosemicarbazide (5)**

Yield: 73%. M.p.: 210 °C. IR (cm^−1^): 2972 (NH), 2855 (CH aliph.), 1612 (C=O), 1510 (CH arom.), 1342 (C=S), 1293 (C-O-C). ^1^H NMR (500 MHz, DMSO-d_6_) δ (ppm):3.07 (m, 4H, 2CH_2_), 3.66 (m 4H, 2CH_2_), 3.70 (s, 3H, OCH_3_), 6.85 (d, 2H, Ar-H, *J* = 9.0 Hz), 7.22 (d, 2H, Ar-H, *J* = 8.4 Hz), 7.34 (d, 1H, Ar-H, *J* = 8.8 Hz), 8.05 (dd, 1H, Ar-H, *J* = 8.8, 2.2 Hz), 8.37 (s, 1H, Ar-H), 9.58 (s, 1H, NH), 9.66 (s, 1H, NH), 10.56 (s, 1H, NH). ^13^C NMR (125 MHz, DMSO-d_6_) δ (ppm):51.18, 55.74, 66.34, 113.77, 120.63, 124.56, 126.70, 132.80, 133.54, 140.34, 147.71, 157.37, 164.57. HRMS (ESI): *m*/*z* = calcd. 432.13362 (M + H+, C19 H21 N5 O5 S), found. 432.13345, diff. 0.385 ppm.

**4-(3-methoxyphenyl)-1-[4-(morpholin-4-yl)-3-nitrobenzoyl]thiosemicarbazide (6)**

Yield: 93%. M.p.: 191 °C. IR (cm^−1^): 3132 (NH), 2963 (CH aliph.), 1673 (C=O), 1530 (CH arom.), 1384 (C=S), 1291 (C-O-C). ^1^H NMR (500 MHz, DMSO-d_6_) δ (ppm): 3.08 (m, 4H, 2CH_2_), 3.67 (m, 4H, 2CH_2_), 3.70 (s, 3H, CH_3_), 6.70 (dd, 1H, Ar-H, *J* = 8.2, 2.5 Hz), 6.98 (d, 1H, Ar-H, *J* = 8.7 Hz), 7.07 (d, 1H, Ar-H), 7.19 (t, 1H, Ar-H, *J* = 8.1 Hz), 7.34 (d, 1H, Ar-H, *J* = 8.8 Hz), 8.05 (dd, 1H, Ar-H, *J* = 8.8, 2.2 Hz), 8.37 (d, 1H, Ar-H, *J* = 2.1 Hz), 9.69 (s, 2H, 2NH), 10.59 (s, 1H, NH). ^13^C NMR (125 MHz, DMSO-d_6_) δ (ppm): 51.17, 55.63, 66.34, 120.55, 124.44, 127.09, 134.42, 140.32, 140.82, 147.76. HRMS (ESI): *m*/*z* = calcd. 432.13362 (M + H+, C19 H21 N5 O5 S), found. 432.13387, diff. 0.587 ppm.

**4-(4-fluorophenyl)-1-[4-(morpholin-4-yl)-3-nitrobenzoyl]thiosemicarbazide (7)**

Yield: 93%. M.p.: 208 °C. IR (cm^−1^): 3144 (NH), 2952 (CH aliph.), 1608 (C=O), 1506 (CH arom.), 1348 (C=S), 1295 (C-O-C). ^1^H NMR (500 MHz, DMSO-d_6_) δ (ppm): 3.08 (m, 4H, 2CH_2_), 3.67 (m, 4H, 2CH_2_), 7.13 (t, 2H, Ar-H, *J* = 8.9 Hz), 7.33–7.37 (m, 3H, Ar-H), 8.05 (dd, 1H, Ar-H, *J* = 8.8 Hz, 2.2 Hz), 8.37 (d, 1H, Ar-H, *J* = 2.2 Hz), 9.73 (s, 1H, NH), 9.75 (s, 1H, NH), 10.60 (s, 1H, NH). ^13^C NMR (125 MHz, DMSO-d_6_) δ (ppm): 51.17, 66.34, 115.35, 120.53, 124.42, 126.70, 128.89, 133.63, 136.02, 140.31, 147.76. ^19^F (470 MHz, DMSO-d_6_) δ (ppm): −117.19. HRMS (ESI): *m*/*z* = calcd. 420.11363 (M + H+, C18 H18 N5 O4 F S), found. 420.11360, diff. 0.071 ppm.

**4-(4-chlorophenyl)-1-[4-(morpholin-4-yl)-3-nitrobenzoyl]thiosemicarbazide (8)**

Yield: 98%. M.p.: 211 °C. IR (cm^−1^): 3109 (NH), 2916 (CH aliph.), 1607 (C=O), 1514 (CH arom.), 1348 (C=S), 1281 (C-O-C). ^1^H NMR (500 MHz, DMSO-d_6_) δ (ppm): 3.08 (m, 4H, 2CH_2_), 3.06 (m, 4H, 2CH_2_), 7.35 (d, 3H, Ar-H, *J* = 8.8 Hz), 7.42–7.45 (m, 2H, Ar-H), 8.06 (dd, 1H, Ar-H, *J* = 8.7 Hz, 2.2 Hz), 8.37 (d, 1H, Ar-H, *J* = 2.1 Hz), 9.78 (s, 2H, 2NH), 10.62 (s, 1H, NH). ^13^C NMR (125 MHz, DMSO-d_6_) δ (ppm): 51.84, 66.34, 120.55, 124.01, 128.20, 138.72, 140.29, 147.78. HRMS (ESI): *m*/*z* = calcd. 436.08408 (M + H+, C18 H18 N5 O4 S Cl), found. 436.08380, diff. 0.641 ppm.

**4-(4-bromophenyl)-1-[4-(morpholin-4-yl)-3-nitrobenzoyl]thiosemicarbazide (9)**

Yield: 97%. M.p.: 199 °C. IR (cm^−1^): 3108 (NH), 2913 (CH aliph.), 1606 (C=O), 1514 (CH arom.), 1348 (C=S), 1260 (C-O-C). ^1^H NMR (500 MHz, DMSO-d_6_) δ (ppm): 3.08 (m, 4H, 2CH_2_), 3.60 (m, 4H, 2CH_2_), 4.35 (d, 1H, Ar-H, *J* = 8.8 Hz), 7.38–7.42 (m, 1H, Ar-H), 7.47 (d, 2H, Ar-H, *J* = 8.7 Hz), 8.05 (dd, 1H, Ar-H, *J* = 8.8 Hz, 2.2 Hz), 8.37 (d, 1H, Ar-H, *J* = 2.2 Hz), 9.80 (s, 2H, 2NH), 10.62 (s, 1H, NH). ^13^C NMR (125 MHz, DMSO-d_6_) δ (ppm): 51.16, 65.94, 120.54, 124.34, 126.09, 126.62, 128.60, 131.38, 131.82, 133.61, 139.15. HRMS (ESI): *m*/*z* = calcd. 480.03356 (M + H+, C18 H18 N5 O4 S Br), found. 480.03374, diff. 0.366 ppm.

**1-[4-(morpholin-4-yl)-3-nitrobenzoyl]-4-[4-(trifluoromethyl)phenyl]thiosemicarbazide (10)**

Yield: 77%. M.p.: 202 °C. IR (cm^−1^): 3107 (NH), 2943 (CH aliph.), 1605 (C=O), 1514 (CH arom.), 1385 (C=S), 1294 (C-O-C). ^1^H NMR (500 MHz, DMSO-d_6_) δ (ppm): 3.08 (m, 4H, 2CH_2_), 3.67 (m, 4H, 2CH_2_), 7.35 (d, 1H, Ar-H, *J* = 8.8 Hz), 7.65 (d, 2H, Ar-H, *J* = 8.7 Hz), 7.70–7.71 (m, 2H, Ar-H), 8.06 (dd, 1H, Ar-H, *J* = 8.8, 2.2 Hz), 8.38 (d, 1H, Ar-H, *J* = 2.2 Hz), 9.94 (s, 2H, 2NH), 10.66 (s, 1H, NH). ^13^C NMR (125 MHz, DMSO-d_6_) δ (ppm): 51.16, 66.34, 113.47, 120.58, 123.78, 124.25, 125.86, 126.33, 126.74, 140.27, 148.21.^19^F NMR (470 MHz, DMSO-d_6_) δ (ppm): –58.75. HRMS (ESI): *m*/*z* = calcd. 470.11044 (M + H+, C19 H18 N5 O4 F3 S), found. 470.11044, diff. 0.008 ppm.

**1-[4-(morpholin-4-yl)-3-nitrobenzoyl]-4-(4-nitrophenyl)thiosemicarbazide (11)**

Yield: 78%. M.p.: 196 °C. IR (cm^−1^): 3109 (NH), 2916 (CH aliph.), 1607 (C=O), 1514 (CH arom.), 1348 (C=S), 1294 (C-O-C). ^1^H NMR (500 MHz, DMSO-d_6_) δ (ppm): 3.07 (m, 4H, 2CH_2_), 3.67 (m, 4H, 2CH_2_), 7.36 (d, 1H, Ar-H, *J* = 8.8 Hz), 7.84–7.86 (m, 2H, Ar-H), 8.06 (dd, 1H, Ar-H, *J* = 8.8, 2.2 Hz), 8.17 (d, 2H, Ar-H, *J* = 9.2 Hz), 8.38 (d, 1H, Ar-H, *J* = 2.2 Hz), 10.07, 10.12, 10.69 (3s, 3H, 3NH). ^13^C NMR DEPT 135 (125 MHz, DMSO-d_6_) δ (ppm): 51.15, 66.33, 120.31, 124.16, 125.45, 126.74, 133.68. HRMS (ESI): *m*/*z* = calcd. 447.10813 (M + H+, C18 H18 N6 O6 S), found. 447.10793, diff. 0.447 ppm.

**4-(2-methoxyphenyl)-1-[4-(morpholin-4-yl)-3-nitrobenzoyl]thiosemicarbazide (12)**

Yield: 93%. M.p.: 211 °C. IR (cm^−1^): 3218 (NH), 2945 (CH aliph.), 1639 (C=O), 1559 (CH arom.), 1351 (C=S), 1291 (C-O-C). ^1^H NMR (500 MHz, DMSO-d_6_) δ (ppm): 3.08 (m, 4H, CH_2_), 3.67 (m, 4H, CH_2_), 3.70 (s, 3H, CH_3_), 6.89 (t, 1H, Ar–H, J = 7.7 Hz), 7.00 (d, 1H, Ar–H, J = 8.2 Hz), 7.14 (t, 1H, Ar–H, J = 7.7 Hz), 7.34 (d, 1H, Ar–H, J = 8.8 Hz), 7.61 (s, 1H, Ar–H), 8.05 (dd, 1H, Ar–H, J = 8.8, 2.2 Hz), 8.37 (d, 1H, Ar–H, J = 2.2 Hz), 9.25 (s, 1H, NH), 9.70 (s, 1H, NH), 10.66 (s, 1H, NH) ppm; ^13^C NMR (125 MHz, DMSO-d_6_) δ (ppm): 51.15, 56.23, 66.34, 112.86, 120.38, 127.44, 133.53 ppm. HRMS (ESI): *m*/*z* = calcd. 432.13362 (M + H+, C19 H21 N5 O5 S), found. 432.13370, diff. 0.193 ppm.

#### 3.1.3. Synthesis of Hydrazone Derivatives (**13**–**21**)

The new hydrazone derivatives were prepared using the method described previously [29,30]. We started by dissolving under heating 0.001 mole of 4-(morpholin-4-yl)-3-nitrobenzhydrazide in 5 mL of anhydrous ethanol. Then, 0.0011 mole of the appropriately substituted benzaldehyde was added, and the reaction mixture was left at room temperature for 24 h. The resulting precipitate was filtered off and air-dried.

**N’-(4-methylbenzylidene)-4-(morpholin-4-yl)-3-nitrobenzhydrazide (13**)

Yield 34%. M.p.: 207 °C. IR (cm^−1^): 3225 (NH), 3030 (CH, arom.), 2859 (CH, aliph.), 1644 (C=O), 1604 (C=N), 1508 (CH arom.), 1288 (C-O-C). ^1^H NMR (500 MHz, DMSO-d_6_) δ (ppm): 2.31 (s, 3H, CH_3_), 3.09 (m, 4H, 2CH_2_), 3.67 (m, 4H, 2CH_2_), 7.24 (d, 2H, Ar-H, *J* = 7.8 Hz), 7.36 (d, 1H, Ar-H, *J* = 8.8 Hz), 7.59 (d, 2H, Ar-H, *J* = 7.8 Hz), 8.07 (dd, 1H, Ar-H, *J* = 8.8, 2.2 Hz), 8.37 (s, 1H, CH), 8.38 (d, 1H, Ar-H, *J* = 2.2 Hz), 11.81 (s, 1H, NH). ^13^C NMR (125 MHz, DMSO-d_6_) δ (ppm): 21.58, 51.16, 66.36, 120.82, 125.06, 126.30, 127.65, 130.02, 132.08, 133.47, 140.34, 140.54, 147.69, 148.48, 161.25. HRMS (ESI): *m*/*z* = calcd. 369.15573 (M + H+, C19 H20 N4 O4), found. 369.15577, diff. 0.104 ppm.

**N’-(4-methoxybenzylidene)-4-(morpholin-4-yl)-3-nitrobenzhydrazide (14)**

Yield: 80%. M.p.: 200 °C. IR (cm^−1^): 3288 (NH), 3212 (CH, arom.), 2970 (CH, aliph.), 1639 (C=O), 1611 (C=N), 1528 (CH arom.), 1288 (C-O-C). ^1^H NMR (500 MHz, DMSO-d_6_) δ (ppm):3.09 (m, 4H, 2CH_2_), 3.67 (m, 4H, 2CH_2_), 3.77 (s, 3H, CH_3_), 6.99 (d, 2H, Ar-H, *J* = 8.8 Hz), 7.35 (d, 1H, Ar-H, *J* = 8.8 Hz), 7.64 (d, 2H, Ar-H, *J* = 8.7 Hz), 8.06 (dd, 1H, Ar-H, *J* = 8.8, 2.2 Hz), 8.35 (s, 1H, CH), 8.37 (d, 1H, Ar-H, *J* = 2.2 Hz), 11.75 (s, 1H, NH). ^13^C NMR (125 MHz, DMSO-d_6_) δ (ppm): 51.17, 55.85, 66.36, 114.90, 120.81, 125.19, 126.24, 127.34, 129.28, 133.44, 140.38, 147.65, 148.95, 161.14, 161.44. HRMS (ESI): *m*/*z* = calcd. 385.15065 (M + H+, C19 H20 N4 O5), found. 385.15070, diff. 0.140 ppm.

**N’-(3-methoxybenzylidene)-4-(morpholin-4-yl)-3-nitrobenzhydrazide (15)**

Yield: 76%. M.p.: 197 °C. IR (cm^−1^): 3217 (NH), 3074 (CH, arom.), 2949 (CH, aliph.), 1640 (C=O), 1612 (C=N), 1582 (CH arom.), 1290 (C-O-C). ^1^H NMR (500 MHz, DMSO-d_6_) δ (ppm): 3.09 (m, 4H, 2CH_2_), 3.67 (m, 4H, 2CH_2_), 3.77 (s, 3H, CH_3_), 6.98 (d, 1H, Ar-H, *J* = 6.8 Hz), 7.25 (d, 1H, Ar-H, *J* = 8.5 Hz), 7.33–7.36 (m, 2H, Ar-H), 8.07 (dd, 1H, Ar-H, *J* = 8.7, 2.2 Hz), 8.38 (bs, 2H, Ar-H, CH=), 11.88 (s, 1H, NH). ^13^C NMR (125 MHz, DMSO-d_6_) δ (ppm): 51.15, 55.71, 66.35, 111.81, 116.80, 120.81, 124.91, 126.37, 130.01, 133.50, 136.22, 139.40, 147.35, 148.34, 160.10. HRMS (ESI): *m*/*z* = calcd. 385.15065 (M + H+, C19 H20 N4 O5), found. 385.15064, diff. 0.016 ppm.

**N’-(2-methoxybenzylidene)-4-(morpholin-4-yl)-3-nitrobenzhydrazide (16)**

Yield: 99%. M.p.: 250 °C. IR (cm^−1^): 3260 (NH), 3040 (CH, arom.), 2970 (CH, aliph.), 1671 (C=O), 1605 (C=N), 1518 (CH arom.), 1292 (C-O-C). ^1^H NMR (500 MHz, DMSO-d_6_) δ (ppm): 3.09 (m, 4H, 2CH_2_), 3.67 (m, 4H, 2CH_2_), 3.83 (s, 3H, CH_3_), 6.99 (t, 1H, Ar-H, *J* = 4.5 Hz), 7.08 (d, 1H, Ar-H, *J* = 4.5 Hz), 7.34–7.40 (m, 2H, Ar-H), 7.83 (d, 1H, Ar-H, *J* = 9.5 Hz), 8.08 (dd, 1H, Ar-H, *J* = 8.8, 2.2 Hz), 8.41 (d, 1H, Ar-H, *J* = 2.3 Hz), 8.76 (s, 1H, CH), 11.82 (s, 1H, NH). ^13^C NMR (125 MHz, DMSO-d_6_) δ (ppm): 51.15, 56.22, 66.36, 112.41, 120.80, 121.31, 122.76, 124.96, 126.02, 126.28, 132.18, 133.49, 140.34, 143.78, 147.68, 158.32, 161.11. HRMS (ESI): *m*/*z* = calcd. 385.15065 (M + H+, C19 H20 N4 O5), found. 385.15077, diff. 0.321 ppm.

**N’-(4-fluorobenzylidene)-4-(morpholin-4-yl)-3-nitrobenzhydrazide (17)**

Yield: 76%. M.p.: 215 °C. IR (cm^−1^): 3188 (NH), 3019 (CH, arom.), 2968 (CH, aliph.), 1640 (C=O), 1612(C=N), 1519 (CH arom), 1283 (C-O-C). ^1^H NMR (500 MHz, DMSO-d_6_) δ (ppm): 3.10 (m, 4H, 2CH_2_), 3.67 (m, 4H, 2CH_2_), 7.07 (t, 2H, Ar-H, *J* = 8.7 Hz), 7.36 (d, 1H, Ar-H, *J* = 8.8 Hz), 7.76 (m, 2H, Ar-H), 8.07 (dd, 1H, Ar-H, *J* = 8.8, 2.2 Hz), 8.38 (d, 1H, Ar-H, *J* = 2.2 Hz), 8.40 (s, 1H, CH), 11.89 (s, 1H, NH). ^13^C NMR (125 MHz, DMSO-d_6_) δ (ppm): 51.52, 65.94, 116.40, 116.57, 120.82, 124.89, 126.35, 129.80, 131.40, 133.43, 140.28, 147.28, 147.73, 161.34, 162.65, 164.77. ^19^F (470 MHz, DMSO-d_6_) δ (ppm): −110.34. HRMS (ESI): *m*/*z* = calcd. 373.13066 (M + H+, C18 H17 N4 O4 F), found. 373.13091, diff. 0.659 ppm.

**N’-(4-chlorobenzylidene)-4-(morpholin-4-yl)-3-nitrobenzhydrazide (18)**

Yield: 67%. M.p.: 191 °C. IR (cm^−1^): 3202 (NH), 3033 (CH, arom.), 2973 (CH, aliph.), 1644 (C=O), 1609 (C=N), 1517 (CH arom.), 1290 (C-O-C). ^1^H NMR (500 MHz, DMSO-d_6_) δ (ppm): 3.10 (m, 4H, 2CH_2_), 3.67 (m, 4H, 2CH_2_), 7.36 (d, 1H, Ar-H, *J* = 8.8 Hz), 7.49 (d, 2H, Ar-H, *J* = 8.1 Hz), 7.72 (d, 2H, Ar-H, *J* = 8.1 Hz), 8.07 (dd, 1H, Ar-H, *J* = 8.8, 2.2 Hz), 8.33 (s, 1H, Ar-H), 8.39 (s, 1H, CH), 11.93 (s, 1H, NH). ^13^C NMR (125 MHz, DMSO-d_6_) δ (ppm): 51.14, 60.35, 120.82, 124.78, 126.40, 129.28, 129.51, 133.52, 133.75, 135.11, 140.25, 147.07, 147.77, 161.39. HRMS (ESI): *m*/*z* = calcd. 389.10111 (M + H+, C18 H17 N4 O4 Cl), found. 389.10107, diff. 0.101 ppm.

**N’-(4-bromobenzylidene)-4-(morpholin-4-yl)-3-nitrobenzhydrazide (19)**

Yield: 43%. M.p.: 214 °C. IR (cm^−1^): 3188 (NH), 3020 (CH, arom.), 2969 (CH, aliph.), 1643 (C=O), 1612 (C=N), 1519 (CH arom.), 1289 (C-O-C). ^1^H NMR (500 MHz, DMSO-d_6_) δ (ppm): 3.10 (m, 4H, 2CH_2_), 3.67 (m, 4H, 2CH_2_), 7.36 (d, 1H, Ar-H, *J* = 8.8 Hz), 7.62–7.66 (m, 4H, Ar-H), 8.07 (dd, 1H, Ar-H, *J* = 8.8, 2.2 Hz), 8.38 (d, 1H, Ar-H, *J* = 2.2 Hz), 8.38 (s, 1H, CH), 11.94 (s, 1H, NH). ^13^C NMR (125 MHz, DMSO-d_6_) δ (ppm): 51.14, 66.35, 120.82, 124.77, 126.40, 128.31, 129.57, 132.42, 133.52, 134.03, 140.24, 147.15, 147.77, 161.39. HRMS (ESI): *m*/*z* = calcd. 433.05059 (M + H+, C18 H17 N4 O4 Br), found. 433.05069, diff. 0.221 ppm.

**4-(Morpholin-4-yl)-3-nitro-N’-(4-(trifluoromethyl)benzylidene)benzhydrazide (20)**

Yield: 74%. M.p.: 302 °C. IR (cm^−1^): 3288 (NH), 3213 (CH, arom.), 2973 (CH, aliph.), 1641 (C=O), 1610 (C=N), 1518 (CH arom.), 1290 (C-O-C). ^1^H NMR (500 MHz, DMSO-d_6_) δ (ppm): 3.09–3.11 (m, 4H, 2CH_2_), 3.67–3.69 (m, 4H, 2CH_2_), 7.36 (d, 1H, Ar-H, *J* = 8.8 Hz), 7.78 (d, 2H, Ar-H, *J* = 8.0 Hz), 7.91 (d, 2H, Ar-H, *J* = 8.0 Hz), 8.08 (dd, 1H, Ar-H, *J* = 8.7, 2.2 Hz), 8.40 (d, 1H, Ar-H, *J* = 2.2 Hz), 8.47 (s, 1H, CH), 12.06 (s, 1H, NH). ^13^C NMR (125 MHz, DMSO-d_6_) δ (ppm): 51.15, 66.36, 120.08, 124.64, 125.20, 126.95, 130.28, 138.76, 147.42. HRMS (ESI): *m*/*z* = calcd. 423.12747 (M + H+, C19 H17 N4 O4 F3), found. 423.12766, diff. 0.459 ppm.

**4-(Morpholin-4-yl)-3-nitro-N’-(4-bromobenzylidene)benzhydrazide (21)**

Yield: 87%. M.p.: 236 °C. IR (cm^−1^): 3260 (NH), 3248 (CH, arom.), 2970 (CH, aliph.), 1639 (C=O), 1611 (C=N), 1515 (CH arom.), 1300 (C-O-C). ^1^H NMR (500 MHz, DMSO-d_6_) δ (ppm): 3.11 (m, 4H, 2CH_2_), 3.68 (m, 4H, 2CH_2_), 7.37 (d, 1H, Ar-H, *J* = 8.8 Hz), 7.95–7.96 (m, 2H, Ar-H), 8.08 (dd, 1H, Ar-H, *J* = 8.8, 2.2 Hz), 8.27 (d, 2H, Ar-H, *J* = 8.4 Hz), 8.41 (d, 1H, Ar-H, *J* = 2.2 Hz), 8.50 (s, 1H, CH), 12.16 (s, 1H, NH). ^13^C NMR (125 MHz, DMSO-d_6_) δ (ppm): 55.10, 56.56, 66.34, 120.87, 124.64, 127.96, 133.56, 141.10, 145.85, 148.84, 163.62. HRMS (ESI): *m*/*z* = calcd. 400.12516 (M + H+, C18 H17 N5 O6), found. 400.12529, diff. 0.326 ppm.

#### 3.1.4. Synthesis of Semicarbazide Derivatives (**22, 23**)

The 0.001 mole of 4-(morpholin-4-yl)-3-nitrobenzhydrazide was added to 10 mL of diethyl ether, and an equimolar amount of isocyanate (4-fluorophenylisocyanate, 4-bromophenylisocyanate) was added to the mixture, then left at room temperature for 24 h. The precipitate was filtered, and the product was air-dried.

**4-(4-Fluorophenyl)-1-[4-(morpholin-4-yl)-3-nitrobenzoyl]semicarbazide (22)**

Yield: 28%**.** M.p.: 200 °C. IR (cm^−1^): 3187 (NH), 3021 (CH, arom.), 2969 (CH, aliph.), 1641 (C=O), 1519 (CH arom.), 1290 (C-O-C). ^1^H NMR (500 MHz, DMSO-d_6_) δ (ppm): 3.07 (m, 4H, CH_2_), 3.08 (m, 4H, CH_2_), 7.05 (t, 2H, Ar-H, *J* = 8.9 Hz), 7.34 (d, 1H, Ar-H, *J* = 8.8 Hz), 7.42–7.45 (m, 2H, Ar-H), 8.05 (dd, 1H, Ar-H, *J* = 8.8, 2.2 Hz), 8.20 (bs, 1H, NH), 8.36 (d, 1H, Ar-H, *J* = 2.2 Hz), 8.86 (bs, 1H, NH), 10.35 (s, 1H, NH). ^13^C NMR (125 MHz, DMSO-d_6_) δ (ppm): 51.17, 66.35, 115.63 (d, *J* = 22.1 Hz), 120.74, 120.99, 124.43, 126.40, 133.32, 136.48, 140.39, 147.70, 156.27, 157.00, 158.90, 164.92. HRMS (ESI): *m*/*z* = calcd. 404.13647 (M + H+, C18 H18 N5 O5 F), found. 404.13604, diff. 1.072 ppm.

**4-(4-Bromophenyl)-1-[4-(morpholin-4-yl)-3-nitrobenzoyl]semicarbazide (23)**

Yield: 43%. M.p.:158 °C. IR (cm^−1^): 3189 (NH), 3018 (CH, arom.), 2968 (CH, aliph.), 1641 (C=O), 1519 (CH arom.), 1301 (C-O-C). ^1^H NMR (500 MHz, DMSO-d_6_) δ (ppm): 3.07 (m, 4H, CH_2_), 3.08 (m, 4H, CH_2_), 7.05 (t, 2H, Ar-H, *J* = 8.9 Hz), 7.34 (d, 1H, Ar-H, *J* = 8.8 Hz), 7.42–7.45 (m, 2H, Ar-H), 8.05 (dd, 1H, Ar-H, *J* = 8.8, 2.2 Hz), 8.20 (bs, 1H, NH), 8.36 (d, 1H, Ar-H, *J* = 2.2 Hz), 8.86 (bs, 1H, NH), 10.35 (s, 1H, NH). ^13^C NMR (125 MHz, DMSO-d_6_) δ (ppm): 51.16, 66.35, 113.95, 120.73, 124.35, 126.41, 131.89, 133.32, 139.64, 147.75, 164.91. HRMS (ESI): *m*/*z* = calcd. 464.05641 (M + H+, C18 H18 N5 O5 Br), found. 464.05588, diff. 1.137 ppm.

### 3.2. Microbiology

#### 3.2.1. Microorganisms

The antimicrobial activity of the tested compounds was evaluated against a panel of either the from the American Type Culture Collection (ATCC) reference microorganisms, including Gram-positive (methicillin-sensitive *Staphylococcus aureus* ATCC 29213, methicillin-resistant *Staphylococcus aureus* ATCC 1707, *Staphylococcus epidermidis* ATCC 12228, *Enterococcus faecalis* ATCC 29212, *Micrococcus luteus* ATCC 10240, *Bacillus subtilis* ATCC 6633, *Bacillus cereus* ATCC 10876) and Gram-negative bacteria (*Escherichia coli* ATCC 25922, *Salmonella* Typhimurium ATCC 14028, *Klebsiella pneumoniae* ATCC 13883, *Pseudomonas aeruginosa* ATCC 27853, *Proteus mirabilis* ATCC 12453). The strains are part of the Department of Pharmaceutical Microbiology collection and were stored at −70 °C in the Tryptic Soy Broth (Biomaxima, Lublin, Poland) containing 30% glycerol (*v*/*v*). The bacteria were then resuspended by transferring 10 µL of the frozen suspension onto sterile Mueller–Hinton agar (Biomaxima, Lublin, Poland) and incubated for 18 ± 2 h at 35 ± 1 °C.

#### 3.2.2. Antibacterial Activity Assay

The microbial suspensions, containing fresh 24 h cultures of bacteria in sterile 0.85% NaCl, were evaluated for 0.5 McFarland standard turbidity. Thereafter, they were diluted 100-fold in a sterile Mueller–Hinton broth (Biomaxima, Poland; MHB) to obtain a final density of 1.5 × 10^6^ CFU/mL (colony forming units, CFU; in ratio of 9 mL broth: 1 mL 0.5 McFarland bacterial suspension). The determination of the antimicrobial activity of the test compounds was determined by the method of twofold dilution of the tested compound in broth, in accordance with the European Committee on Antimicrobial Susceptibility Testing (EUCAST) recommendations [38], as previously described [39]. Utilizing this method, the minimum inhibitory concentration (MIC) of the compound required to inhibit the growth of the testing bacteria was measured. This method was used to test for the minimum bactericidal concentration (MBC), the lowest concentration of compound or antibiotic needed to kill the testing bacteria. Next, the MBC/MIC ratio was calculated in terms of bacteriostatic (MBC/MIC ≥4) or bactericidal (MBC/MIC < 4) activity against bacterial growth and proliferation. The powdered test compounds were dissolved in 1 mL DMSO (POCH, Poland) and then diluted in MHB broth to a concentration of 1000 µg/mL. The bactericidal activity of the compounds was then assessed in the range of 0.49–1000 µg/mL. Two antibiotics were selected for use as positive controls: amoxicillin (5 mg/mL, Glentham Life Sciences, Corsham, UK) from the beta-lactam group and colistin sulfate salt (1 mg/mL, Sigma Aldrich, St. Louis, MO, USA) from the polymyxin group.

### 3.3. The Single Crystal X-Ray Diffraction Analysis

Diffraction intensities from crystals **12**, **15,** and **23** were measured by the ω scan technique at room temperature with a SuperNova diffractometer (Cu, *λ* = 1.54184 Å). Crystal structure refinement details are given in Table S1. The models of the crystal structures were found using ShelxS software and refined with the ShelxT package implemented in Olex2-1.5 [40,41]. Crystal **23** crystallizes as a solvate. Because the methanol molecules were disordered over three positions with sof 1/3, they were refined isotropically. Additionally, one of the nitro groups was constrained by the EADP command for O2A and O3A atoms. Drawings were prepared using Mercury 4.0 [42]. Additional information on molecular geometry is given in Supplementary Materials.

Crystal Data for **12** C_19_H_21_N_5_O_5_S (*M* =431.47 g/mol): triclinic, space group *P*-1 (no. 2), *a* = 7.7989(7) Å, *b* = 10.6926(10) Å, *c* = 12.3918(9) Å, *α* = 102.004(7)°, *β* = 94.181(7)°, *γ* = 95.909(7)°, *V* = 1000.66(15) Å^3^, *Z* = 2, *T* = 293(2) K, μ(Cu Kα) = 1.814 mm^−1^, *D_calc_* = 1.432 g/cm^3^, 6822 reflections measured (7.328° ≤ 2Θ ≤ 152.118°), 4034 unique (*R*_int_ = 0.0328, R_sigma_ = 0.0584) which were used in all calculations. The final *R*_1_ was 0.0539 (I > 2σ(I)) and *wR*_2_ was 0.1478 (all data). CCDC No. 2445821.

Crystal data for **15** C_19_H_20_N_4_O_5_ (*M* =384.39 g/mol): monoclinic, space group *P*2_1_/*c* (no. 14), *a* = 8.5020(2) Å, *b* = 26.7674(8) Å, *c* = 8.1607(2) Å, *β* = 90.207(2)°, *V* = 1857.17(8) Å^3^, *Z* = 4, *T* = 293(2) K, μ(Cu K_α_) = 0.847 mm^−1^, *D_calc_* = 1.375 g/cm^3^, 13,220 reflections measured (10.404° ≤ 2Θ ≤ 151.95°), 3820 unique (*R*_int_ = 0.0380, R_sigma_ = 0.0340) which were used in all calculations. The final *R*_1_ was 0.0473 (I > 2σ(I)) and *wR*_2_ was 0.1407 (all data). CCDC No. 2445822.

Crystal data for **23** C_37_H_40_Br_2_N_10_O_11_ (*M* =960.61 g/mol): monoclinic, space group *P*2_1_/*c* (no. 14), *a* = 12.7298(2) Å, *b* = 14.8328(3) Å, *c* = 24.4071(4) Å, *β* = 96.6110(10)°, *V* = 4577.87(14) Å^3^, *Z* = 4, *T* = 293(2) K, μ(Cu K_α_) = 2.798 mm^−1^, *D_calc_* = 1.394 g/cm^3^, 33,144 reflections measured (6.986° ≤ 2Θ ≤ 152.34°), 9434 unique (*R*_int_ = 0.0424, R_sigma_ = 0.0358) which were used in all calculations. The final *R*_1_ was 0.0714 (I > 2σ(I)), and *wR*_2_ was 0.2468 (all data). CCDC No. 2447754.

Crystal structure data were deposited in the Cambridge Crystallographic Data Center and can be obtained free of charge via www.ccdc.cam.ac.uk/data_request/cif (or from the CCDC, 12 Union Road, Cambridge CB2 1EZ, UK; Fax: +44 1223 336033; E-mail: deposit@ccdc.cam.ac.uk).

### 3.4. Quantum Chemical Calculations

All calculations were made in the “Cyfronet” computational center in Kraków based on the PlGrid infrastructure (“Ares” cluster). The DFT calculations were conducted using the GAUSSIAN 16 package [43] and the hybrid ωB97X-D functional, which includes the long-range exchange correction and the semiclassical London-dispersion correction. The standard 6-311G(d,p) basis set, which has d-type polarization for second row elements, and d-type polarization functions for hydrogen atoms ωB97X-D/6-311g(d,p) was used in the analyses. The same function has been recently used for the prediction of many different types of local activity of organic compounds, including oxygen, nitrogen, and sulfur heteroatoms [44,45,46,47]. It should be noted that for the verification of the usability of this level of theory for illustration of geometries of tested molecules, we compared key geometrical parameters of compound **23** derived from RTG analyses, with respective parameters from structures optimized by ωB97X-D/6-311G(d,p) computations (Table 2).

The solvent effects of water were assessed by full optimization of the gas-phase structures at the same computational level using the polarizable continuum model (PCM) in the framework of the self-consistent reaction field (SCRC). Molecular electrostatic potential (MEP) maps were visualized using the GaussView6 program.

## 4. Conclusions

A rationally designed methodology that may assure significant enhancement of antimicrobial activity of substituted aromatic hydrazides was presented in the case of terminal -NH_2_ group functionalization. It has been demonstrated that derivatives of nitro morpholyl benzhydrazides are biologically active and their antimicrobial action can be explained neither by the coordination interaction with biogenic metal cations that are present in the enzyme nor by nonspecific toxicity caused by morpholyl and nitro groups but rather by formation of strong hydrogen bonds that significantly facilitate the activity of maternal hydrazide against the Gram-positive bacteria. The semicarbazides are therefore much more active than thiosemicarbazide- and hydrazone-type derivatives. The structures of new compounds were confirmed by the single crystal X-ray diffraction analyses, and their properties were predicted by advanced quantum chemical calculations, which prove the superiority of semicarbazides in hydrogen bond formation, while their affinity to the transition metal atom cations was proven to be low. Although additional studies on identifying the molecular targets affected by the tested compounds, structure–activity relationships (SAR), and the toxicological properties of active semicarbazides are crucial for the rational development of potent drug candidates and still need to be performed, the presented studies shed light on a clear path toward the coveted and long-awaited new antibacterial substances.

## Figures and Tables

**Table 1:** Antibacterial activity results of the newly synthesized compounds with amoxicillin and colistin as standard antibiotics.

	Gram-Positive Bacteria							Gram-Negative Bacteria					
	*S. aureus* ATCC 29213	*S. aureus* ATCC 1707	*S. epidermidis* ATCC 12228	*M. luteus* ATCC 10240	*E. faecalis* ATCC 29212	*B. subtilis* ATCC 6633	*B. cereus* ATCC 10876	*E. coli* ATCC 25922	*K. pneumoniae* ATCC 13883	*P. aeruginosa* ATCC 27853	*P. mirabilis* ATCC 12453	*A. baumannii* ATCC 19606	*S. Typhimurium* ATCC 14028
**Compounds**	**MIC [µg/mL]**												
**4**	1000	1000	>1000	500	500	>1000	1000	>1000	>1000	1000	>1000	1000	>1000
**5**	>1000	1000	>1000	>1000	1000	>1000	>1000	>1000	>1000	>1000	>1000	1000	>1000
**7**	>1000	1000	>1000	>1000	500	>1000	>1000	>1000	>1000	>1000	>1000	1000	>1000
**8**	>1000	>1000	250	1000	250	>1000	1000	1000	>1000	>1000	>1000	1000	>1000
**9**	>1000	1000	125	125	500	>1000	250	>1000	>1000	>1000	>1000	1000	>1000
**10**	>1000	1000	62.5	31.25	62.5	62.5	31.25	500	>1000	>1000	>1000	1000	>1000
**11**	1000	>1000	>1000	500	250	1000	250	>1000	>1000	1000	>1000	1000	>1000
**12**	>1000	>1000	>1000	>1000	125	>1000	>1000	1000	>1000	1000	>1000	>1000	>1000
**22**	1000	1000	15.625	7.81	15.625	31.25	15.625	500	1000	1000	1000	>1000	1000
**23**	500	1000	7.81	62.5	3.91	>1000	125	>1000	1000	1000	1000	1000	1000
**amoxicillin**	0.781	>100	12.5	0.39	0.39	0.098	>100	>100	>100	>100	0.391	50	0.781
**colistin**	12.5	<0.0488	31.25	0.781	100	0.39	100	50	0.391	0.195	100	<0.0488	<0.0488

**Table 2:** Comparison of the key geometrical parameters of model compounds derived from RTG analyses, with respective parameters from structures optimized by ωB97X-D/6-311G(d,p) quantum chemical calculations.

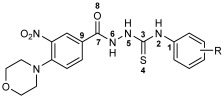		
Parameter	DFT (9)	Rtg (12)
R	4-Br	2-MeO
d_12_ [Å]	1.425	1.422
d_23_ [Å]	1.347	1.343
d_34_ [Å]	1.683	1.665
d_35_ [Å]	1.360	1.360
d_56_ [Å]	1.366	1.382
d_67_ [Å]	1.374	1.334
d_78_ [Å]	1.213	1.235
d_79_ [Å]	1.493	1.486
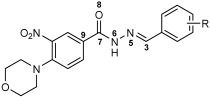		
**Parameter**	**DFT (19)**	**Rtg (15)**
R	4-Br	3-MeO
d_35_ [Å]	1.273	1.270
d_56_ [Å]	1.358	1.381
d_67_ [Å]	1.369	1.342
d_78_ [Å]	1.215	1.227
d_79_ [Å]	1.497	1.493
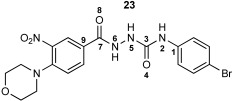		
Parameter	DFT	Rtg
d_12_ [Å]	1.40	1.40
d_23_ [Å]	1.36	1.34
d_34_ [Å]	1.22	1.23
d_35_ [Å]	1.39	1.38
d_56_ [Å]	1.37	1.38
d_67_ [Å]	1.36	1.35
d_78_ [Å]	1.22	1.23
d_79_ [Å]	1.49	1.48

## Data Availability

Data are contained within the article and Supplementary Materials.

## Supplementary Materials

The following supporting information can be downloaded at: https://www.mdpi.com/article/10.3390/molecules30163343/s1, ^1^H NMR spectra for compounds **3**–**23**; ^13^C NMR spectra for compounds **3**–**23**; ^19^F NMR spectra for compounds **10**, **17**, **20**; Crystallographic data for crystals **12**, **15** and **23**; IR spectra for compounds **3**–**23**.
