# Supplementary material for: New 4-(Morpholin-4-Yl)-3-Nitrobenzhydrazide Based Scaffold: Synthesis, Structural Insights, and Biological Evaluation
Source: Molecules. 2025 Aug 11;30(16):3343. doi: 10.3390/molecules30163343 (PMC12388722; doi:10.3390/molecules30163343)
Supplement: Supplementary file 1 [file molecules-30-03343-s001.zip › molecules-3782507-supplementary.pdf]

New 4-(Morpholin-4-yl)-3-nitrobenzhydrazide Based Scaffold: Synthesis, Structural Insights and Biological Evaluation

Michał Janowski [1], Sara Janowska [2], Sylwia Andrzejczuk [3], Urszula Kosikowska [3], Radomir Jasiński [4]*, Barbara Mirosław [5], Marcin Feldo [6], Monika Wujec [7], Oleg M. Demchuk [8]*

1 Doctoral School, Medical University of Lublin, 7 Chodzki Str., 20-093 Lublin, Poland;
2 Department of Pathobiochemistry and Interdisciplinary Applications of Ion Chromatography, Medical University of Lublin, 1 Chodzki Str., 20-093 Lublin, Poland;
3 Department of Pharmaceutical Microbiology, Medical University of Lublin, 1 Chodzki Str., 20-093 Lublin, Poland;
4 Department of Organic Chemistry and Technology, University of Technology in Cracow, Poland;
5 Department of General and Coordination Chemistry and Crystallography, Institute of Chemical Sciences, Faculty of Chemistry, Maria Curie-Sklodowska University in Lublin, Maria Curie-Sklodowska sq. 2, 20-031 Lublin, Poland;
6 Chair and Department of Vascular Surgery and Angiology, Medical University of Lublin, 11 Staszica St., 20-081 Lublin, Poland;
7 Department of Organic Chemistry, Medical University of Lublin, 4a Chodzki Str., 20-093 Lublin, Poland;
8 Faculty of Medicine, The John Paul II Catholic University of Lublin, Konstantynów 1J, 20-708 Lublin, Poland.

* Correspondence: radomir.jasinski@pk.edu.pl; oleh.demchuk@kul.pl

Supplementary Information

Table of contents:

1. ^{1}H NMR spectra for compounds **3 - 23**……………………………………………………….. 2

2. ^{13}C NMR spectra for compounds **3 - 23**………………...…………………………...…... 23

3. ^{19}F NMR spectra for compounds **10**, **17**, **20**.………………...…………..………………..…. 44

4. Supplementary crystallographic data for crystals **12**, **15** and **23**..…………………….. 47

5. IR spectra for compounds **3 - 23**.. 59

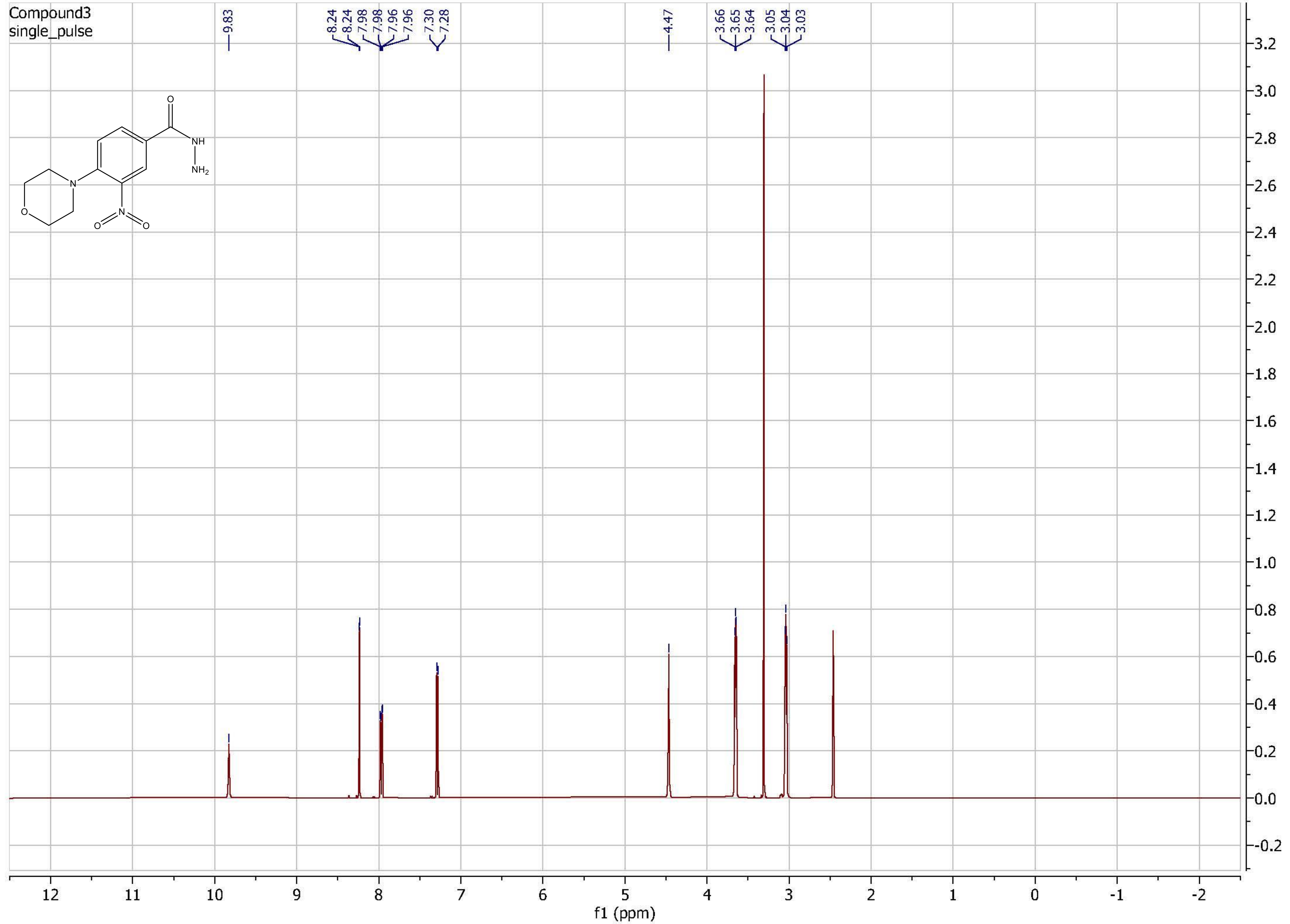

Figure S1. The ^{1}H NMR of compound **3.**

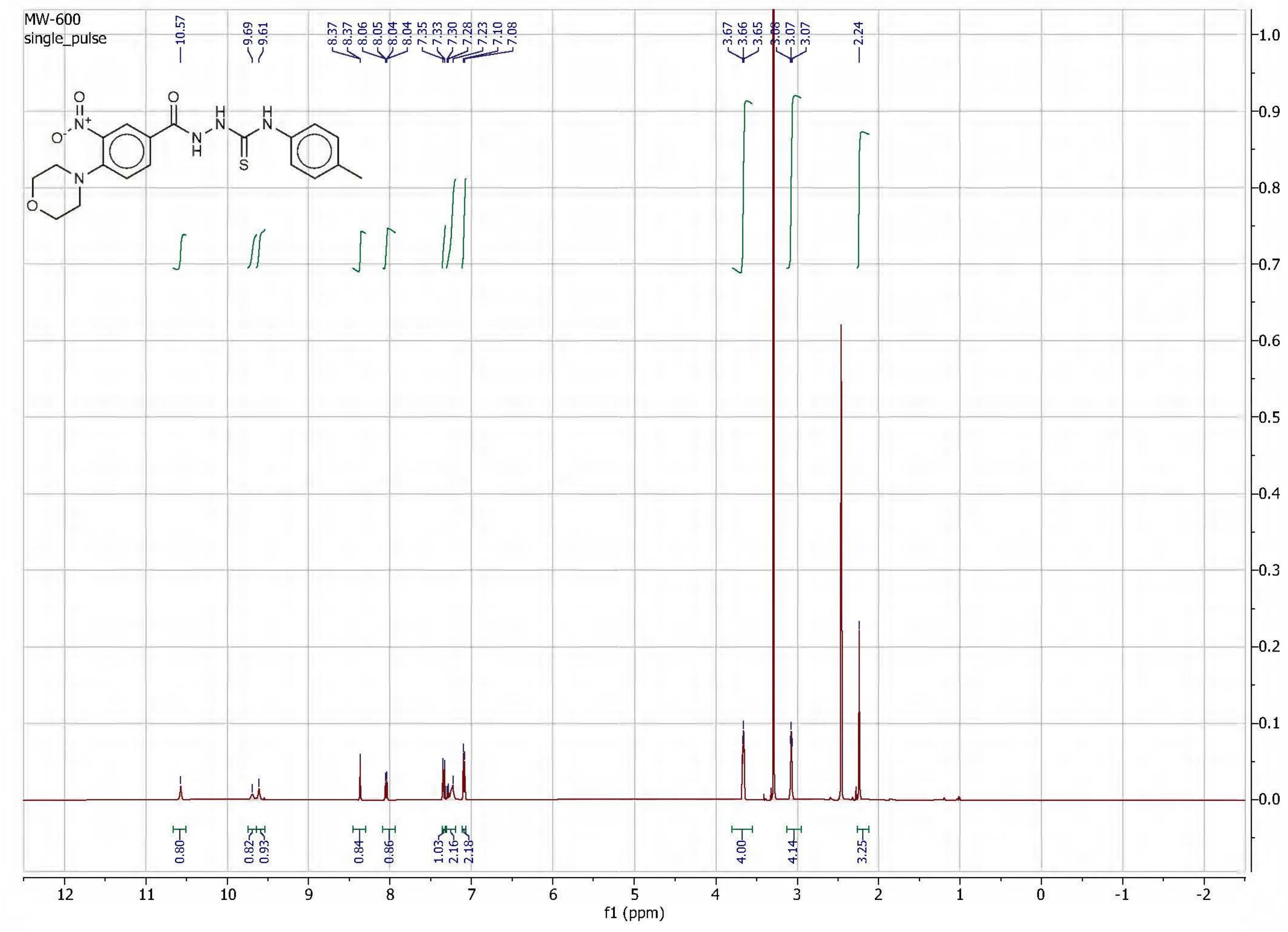

Figure S2. The ^{1}H NMR of compound **4.**

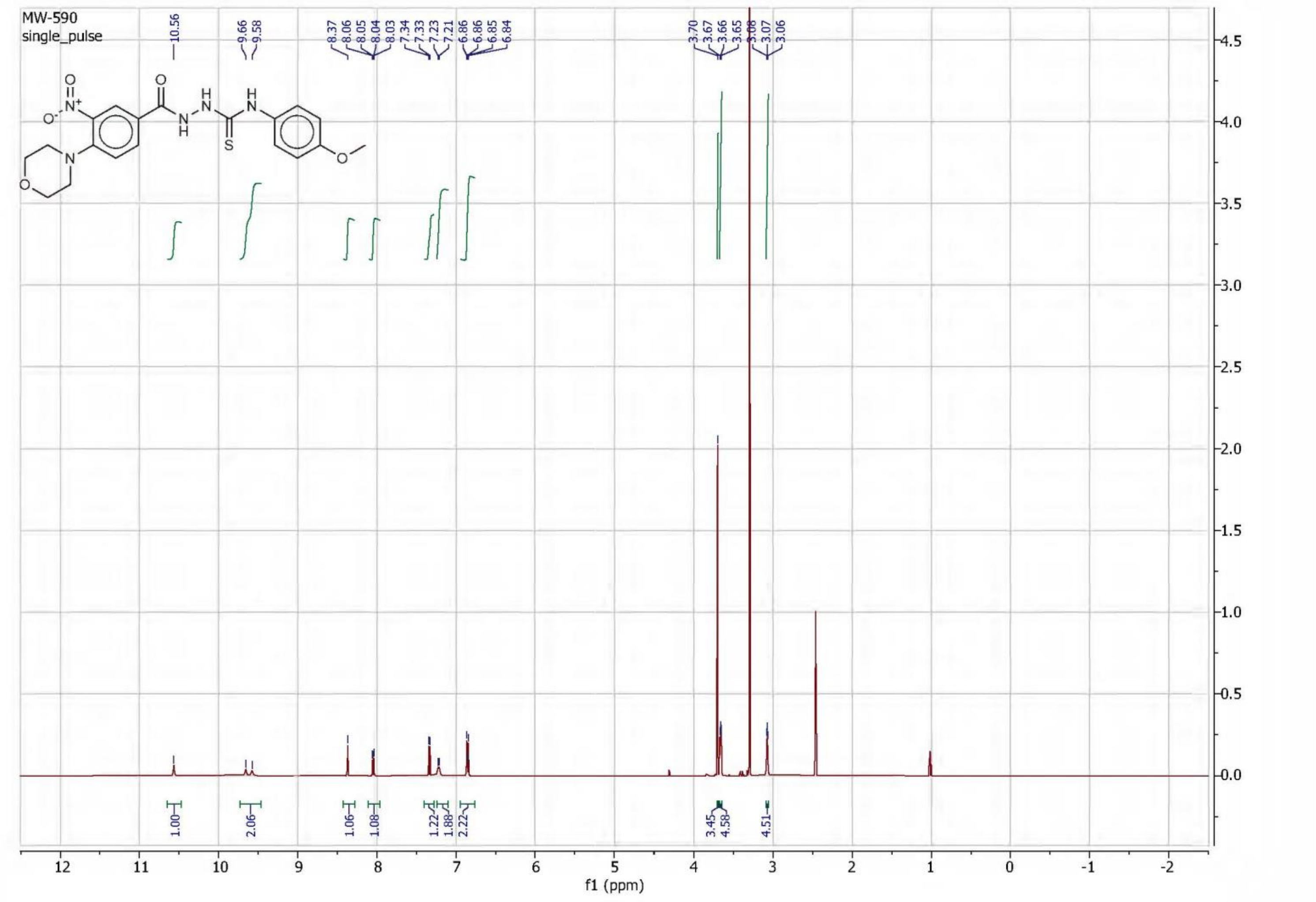

Figure S3. The ^{1}H NMR of compound **5.**

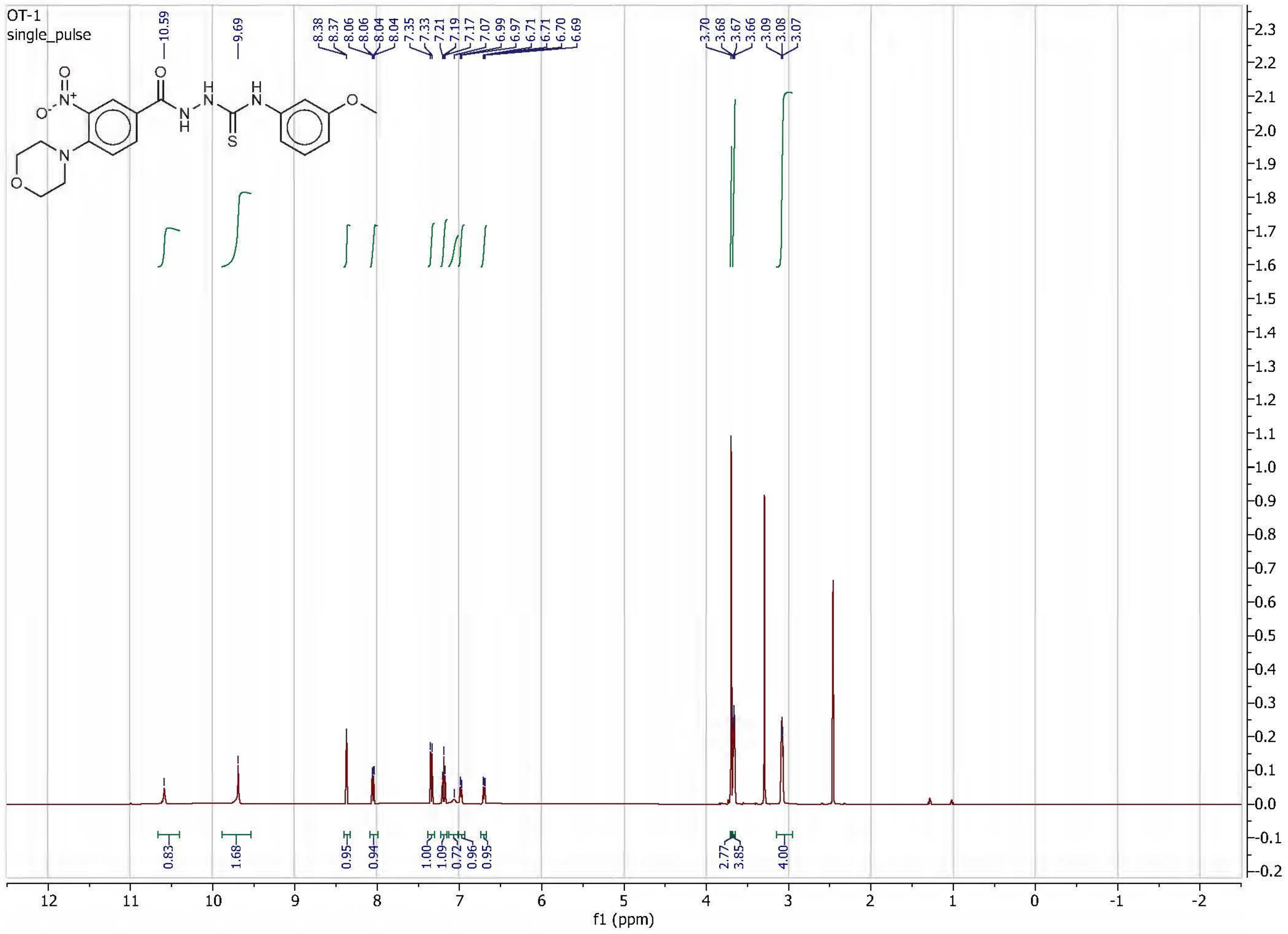

Figure S4. The ^{1}H NMR of compound **6**.

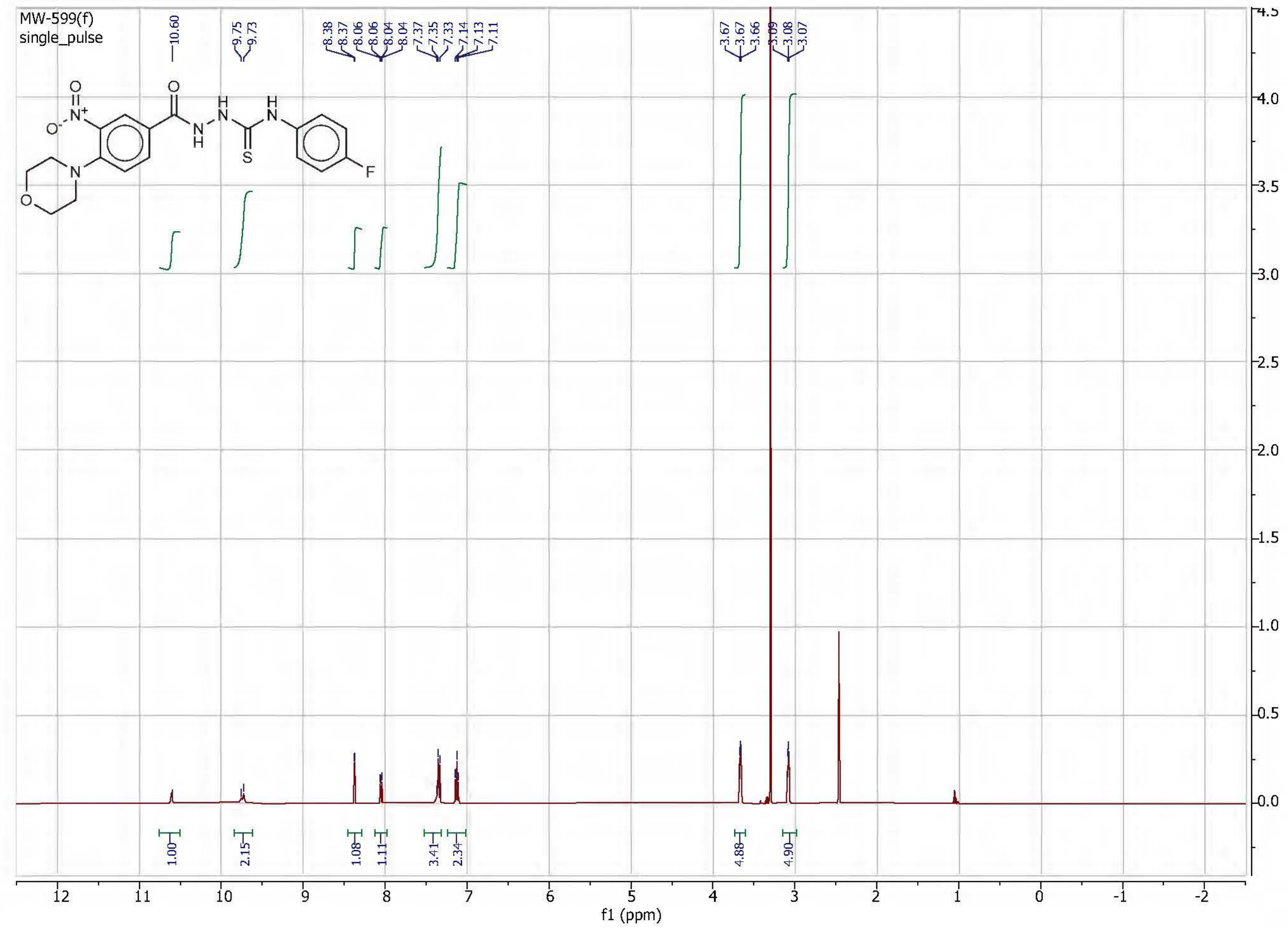

Figure S5. The ^{1}H NMR of compound **7**.

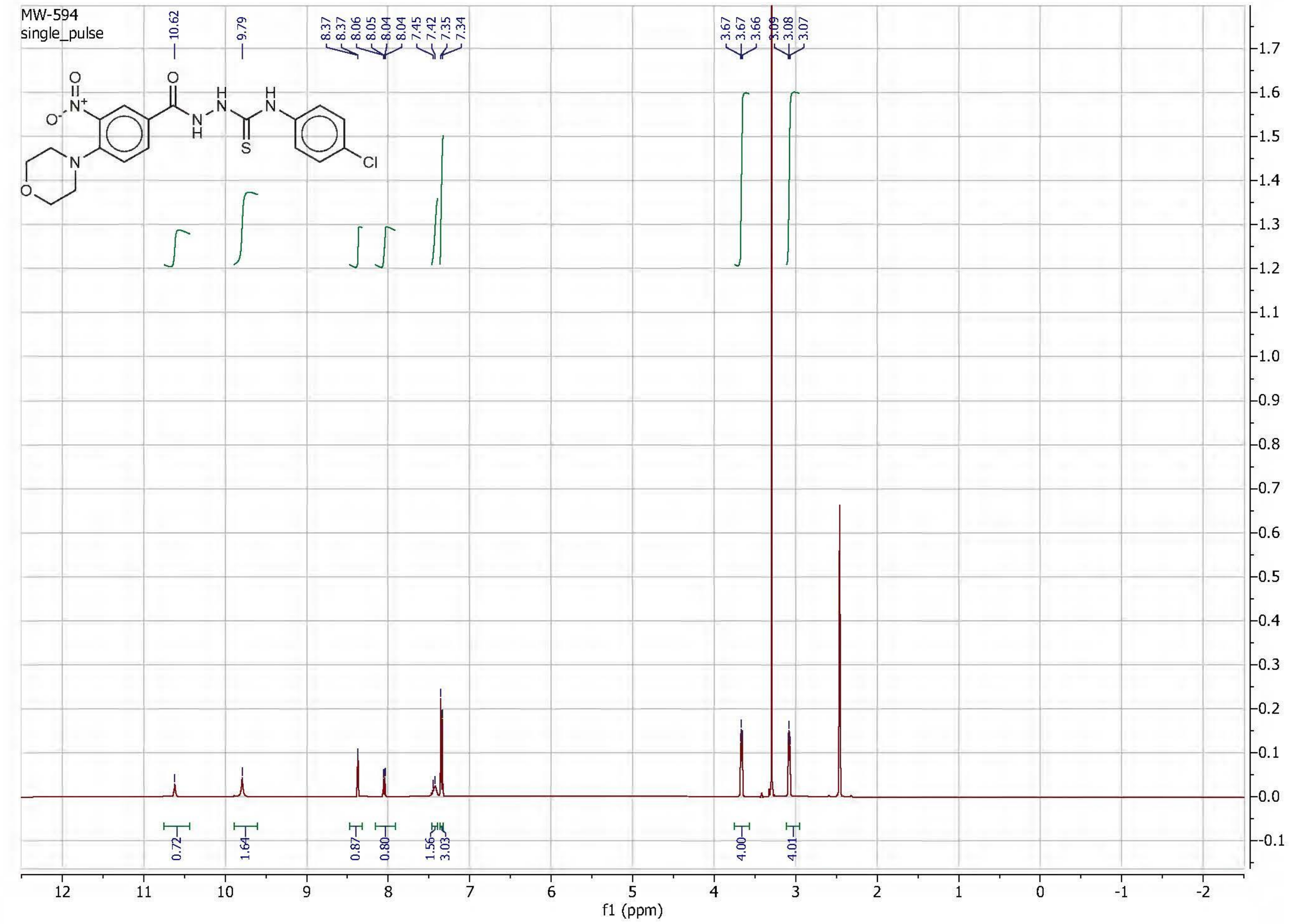

Figure S6. The ^{1}H NMR of compound **8**.

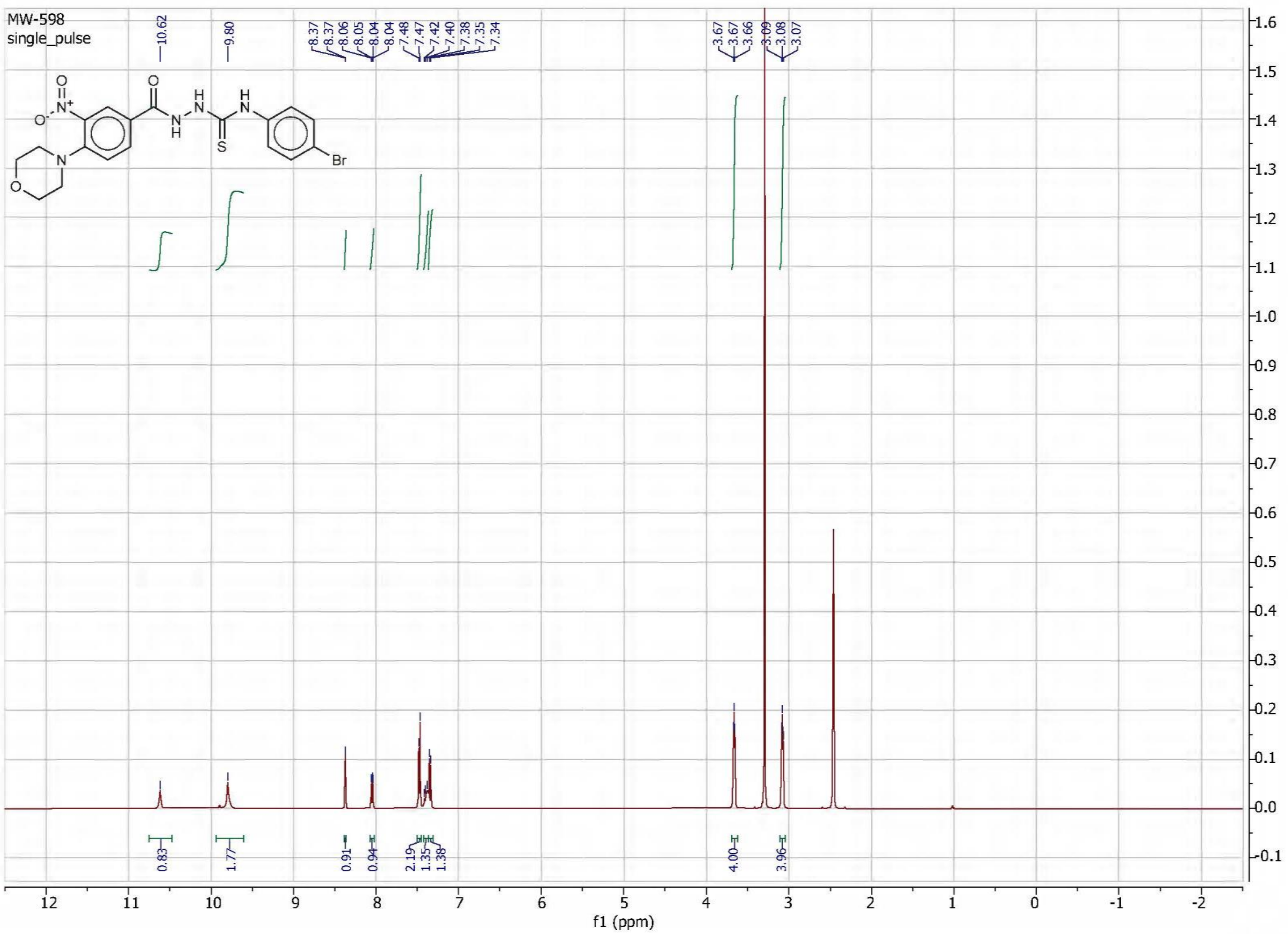

Figure S7. The ^{1}H NMR of compound **9**.

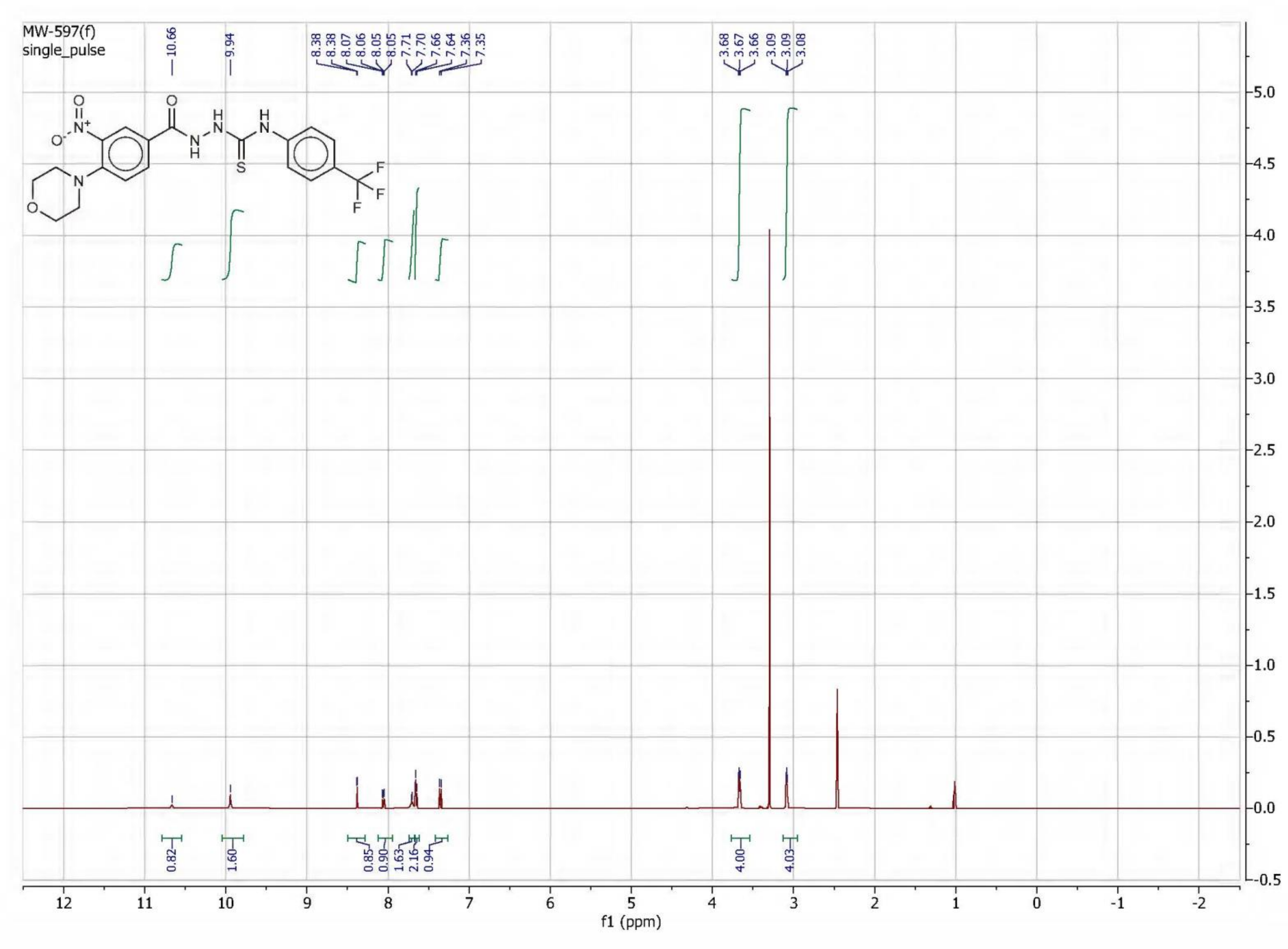

Figure S8. The ^{1}H NMR of compound **10**.

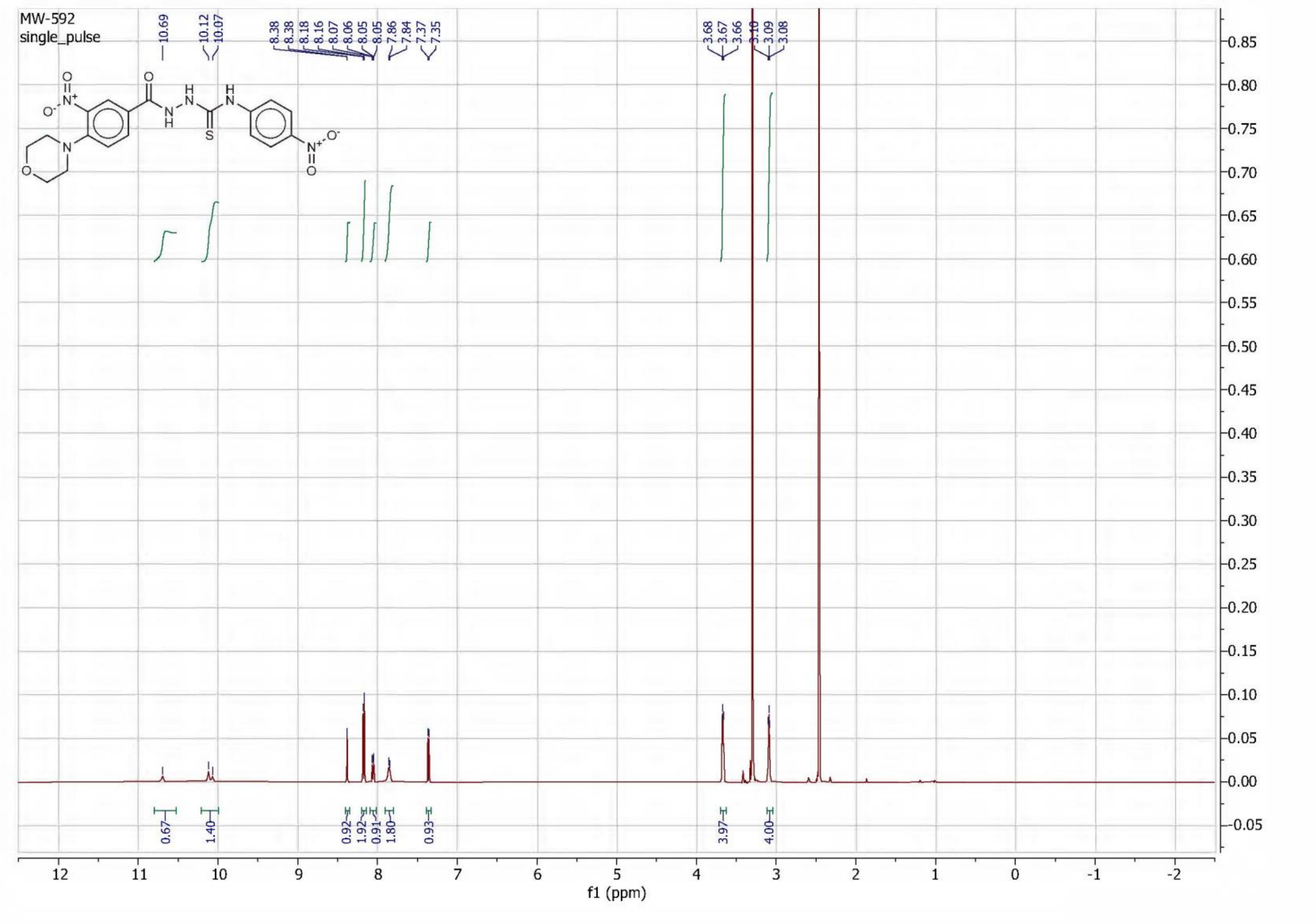

Figure S9. The ^{1}H NMR of compound **11**.

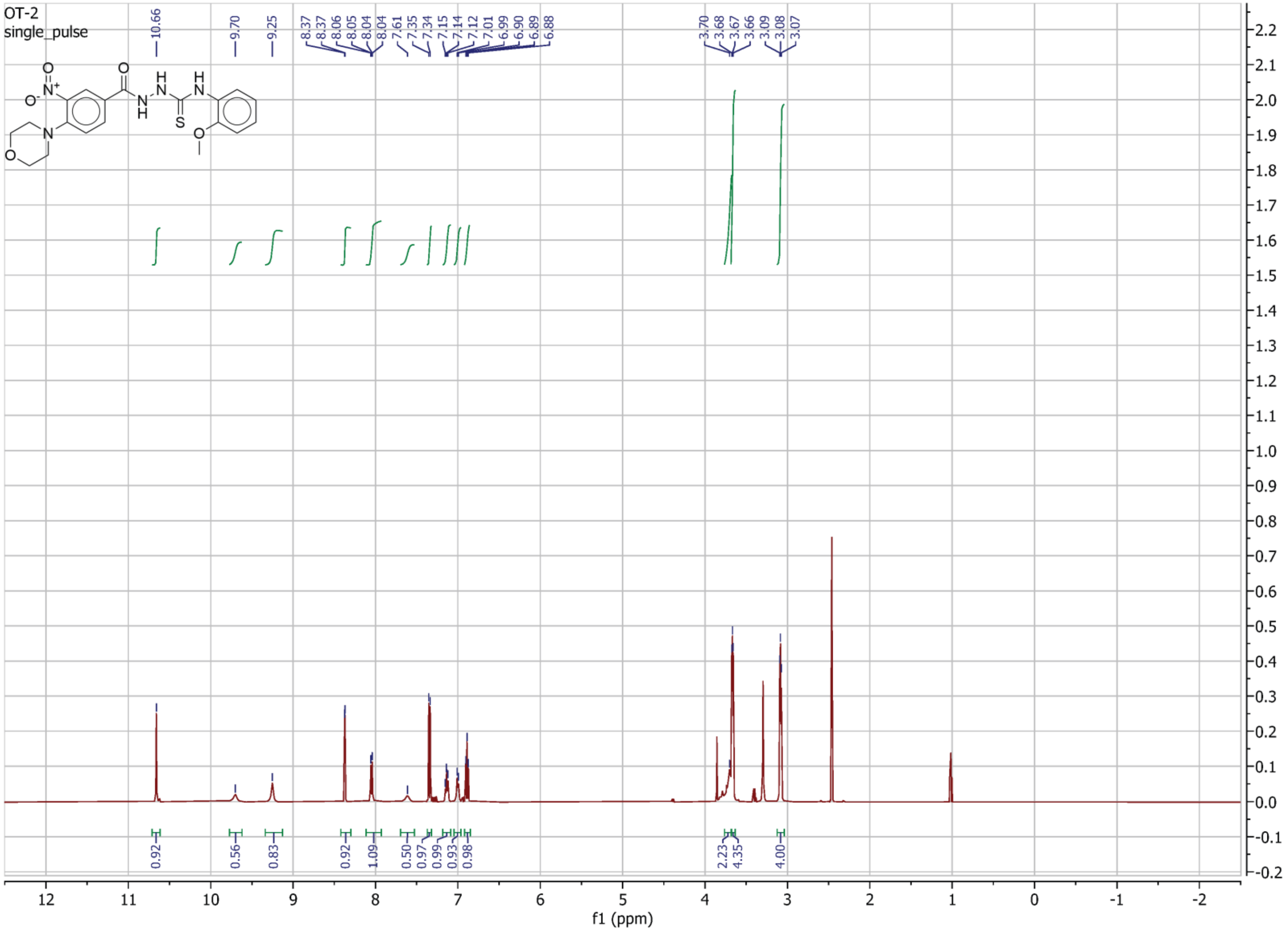

Figure S10. The ¹H NMR of compound **12**.

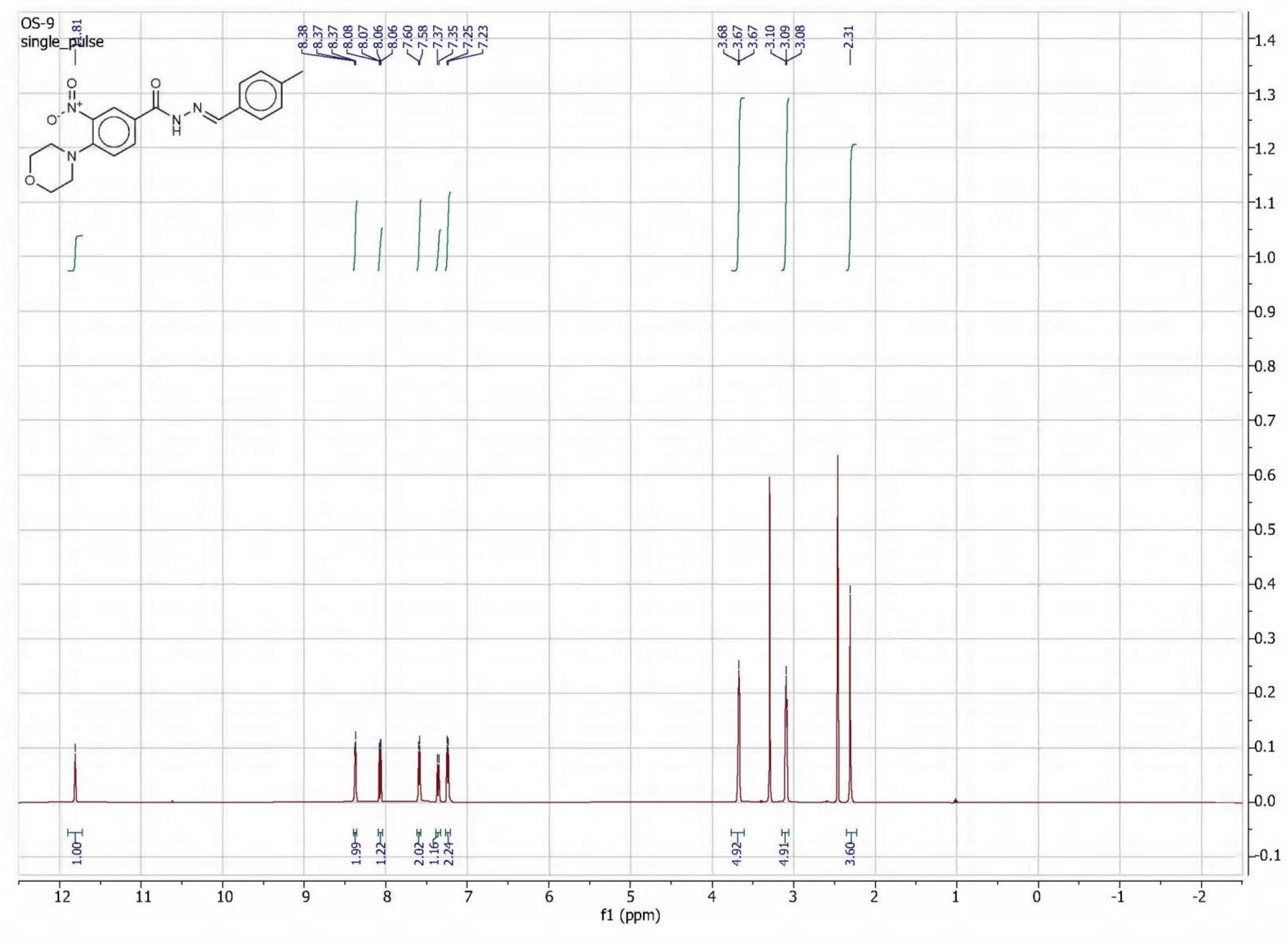

Figure S11. The ^{1}H NMR of compound **13**.

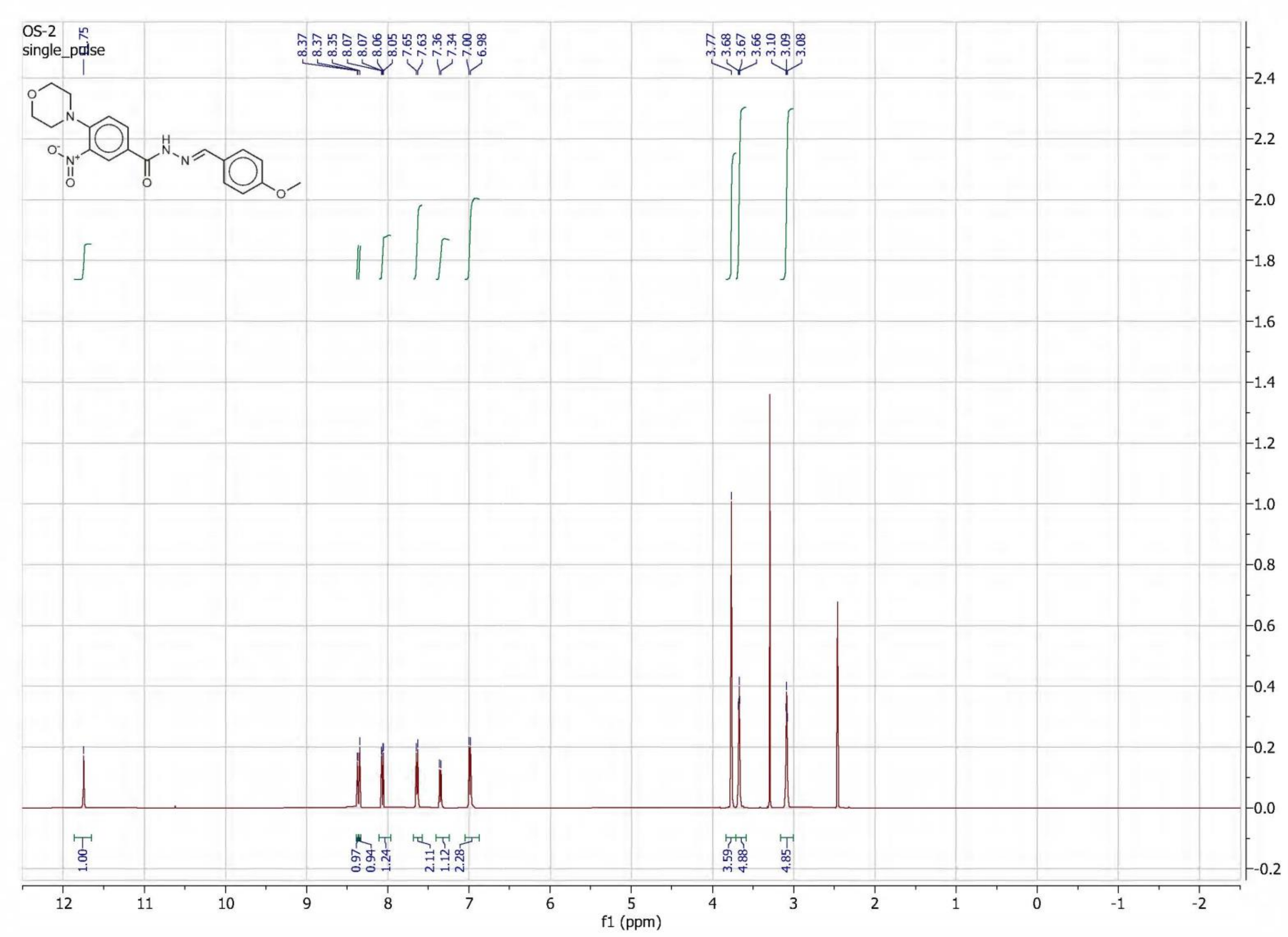

Figure S12. The ^{1}H NMR of compound **14**.

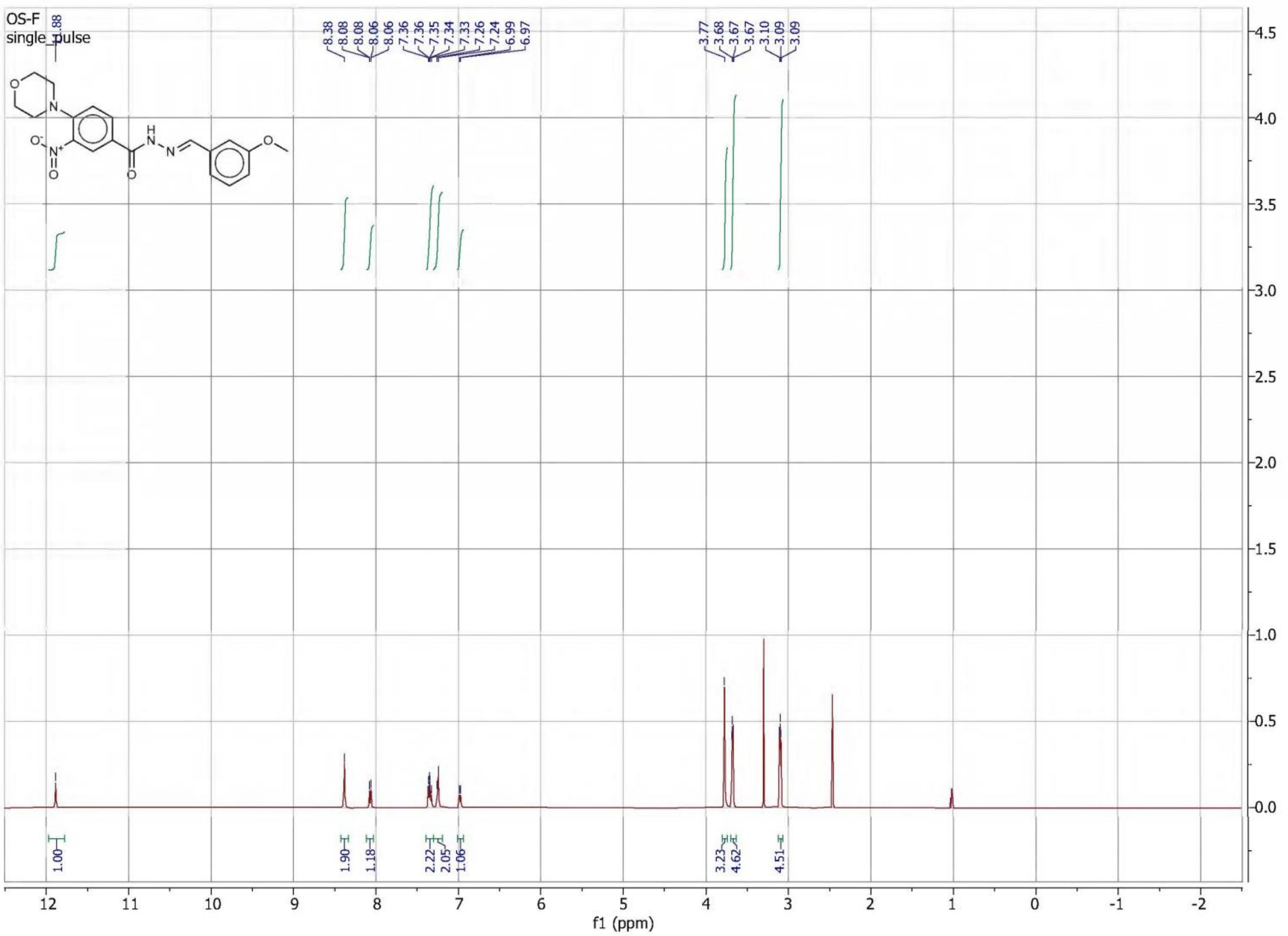

Figure S13. The ^{1}H NMR of compound **15**.

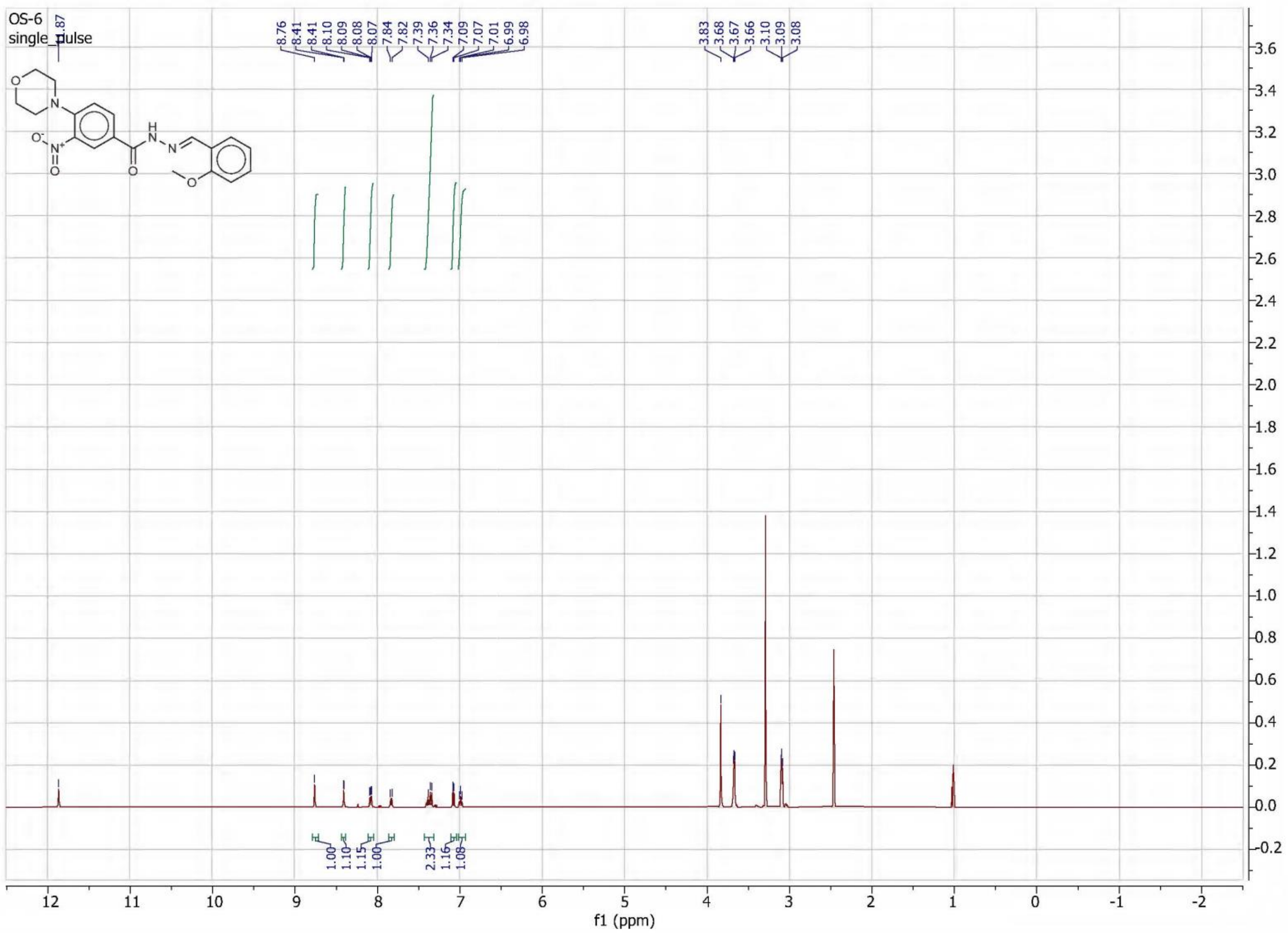

Figure S14. The ^{1}H NMR of compound **16**.

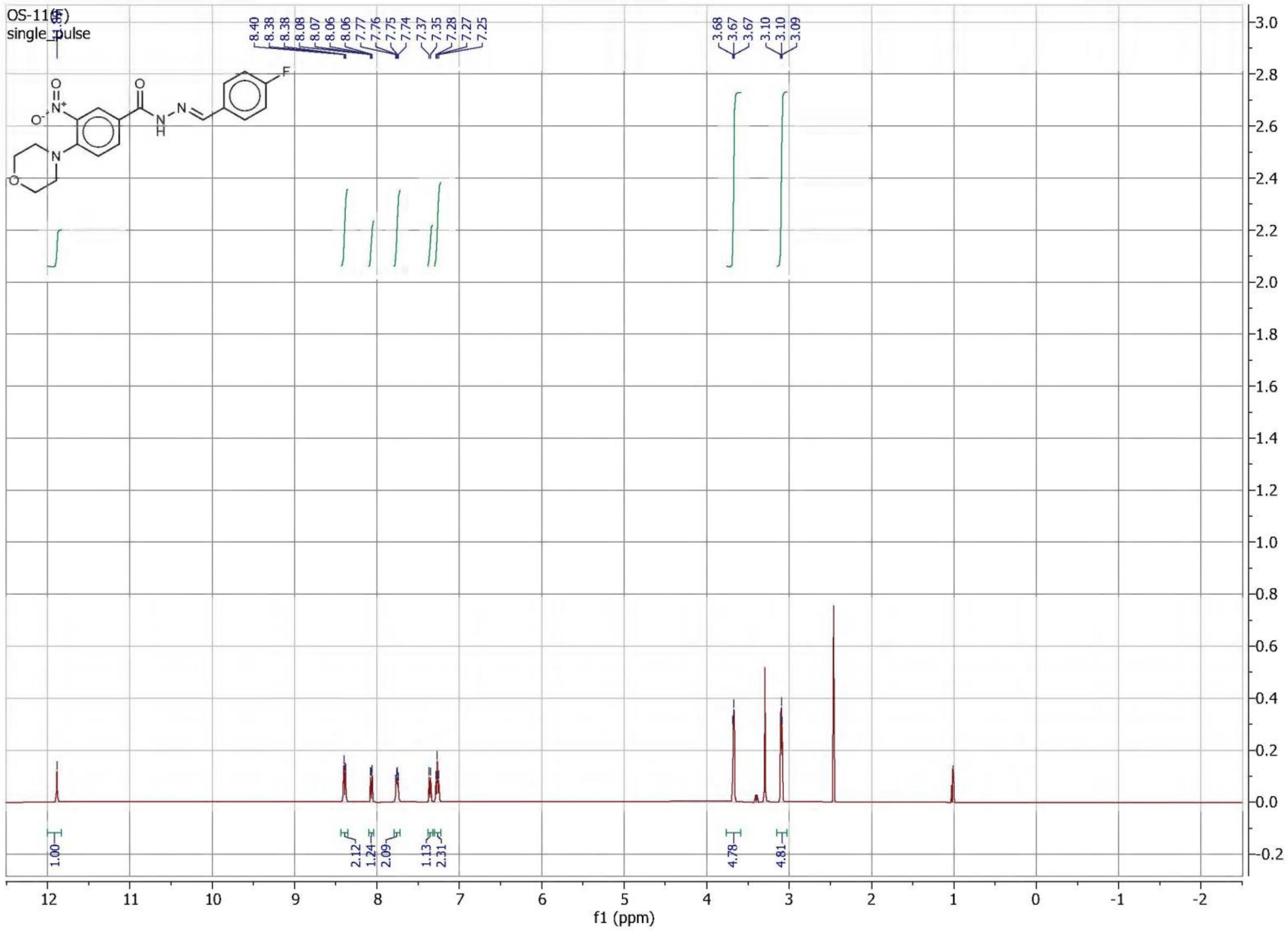

Figure S15. The ^{1}H NMR of compound **17**.

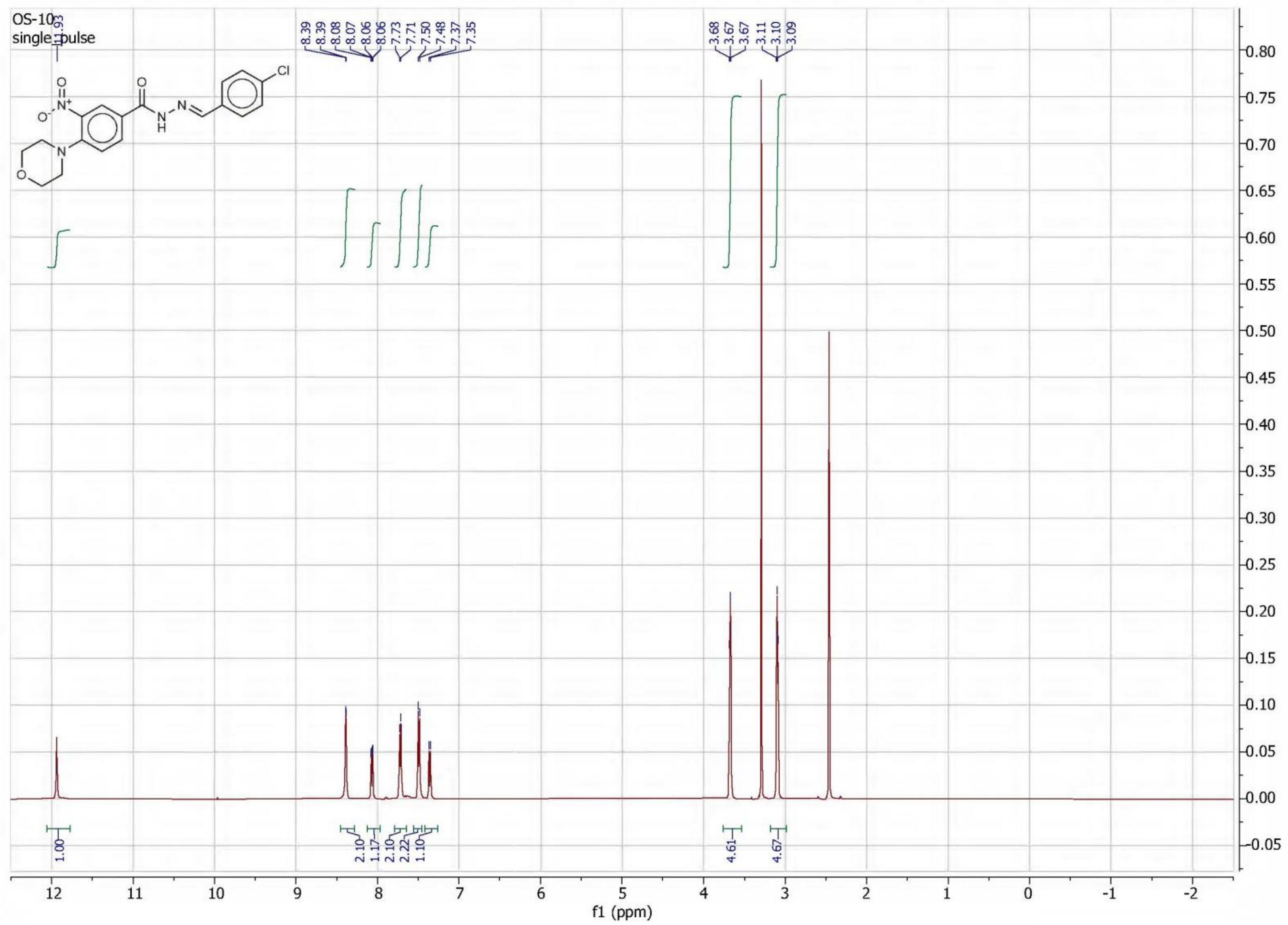

Figure S16. The ^{1}H NMR of compound **18**.

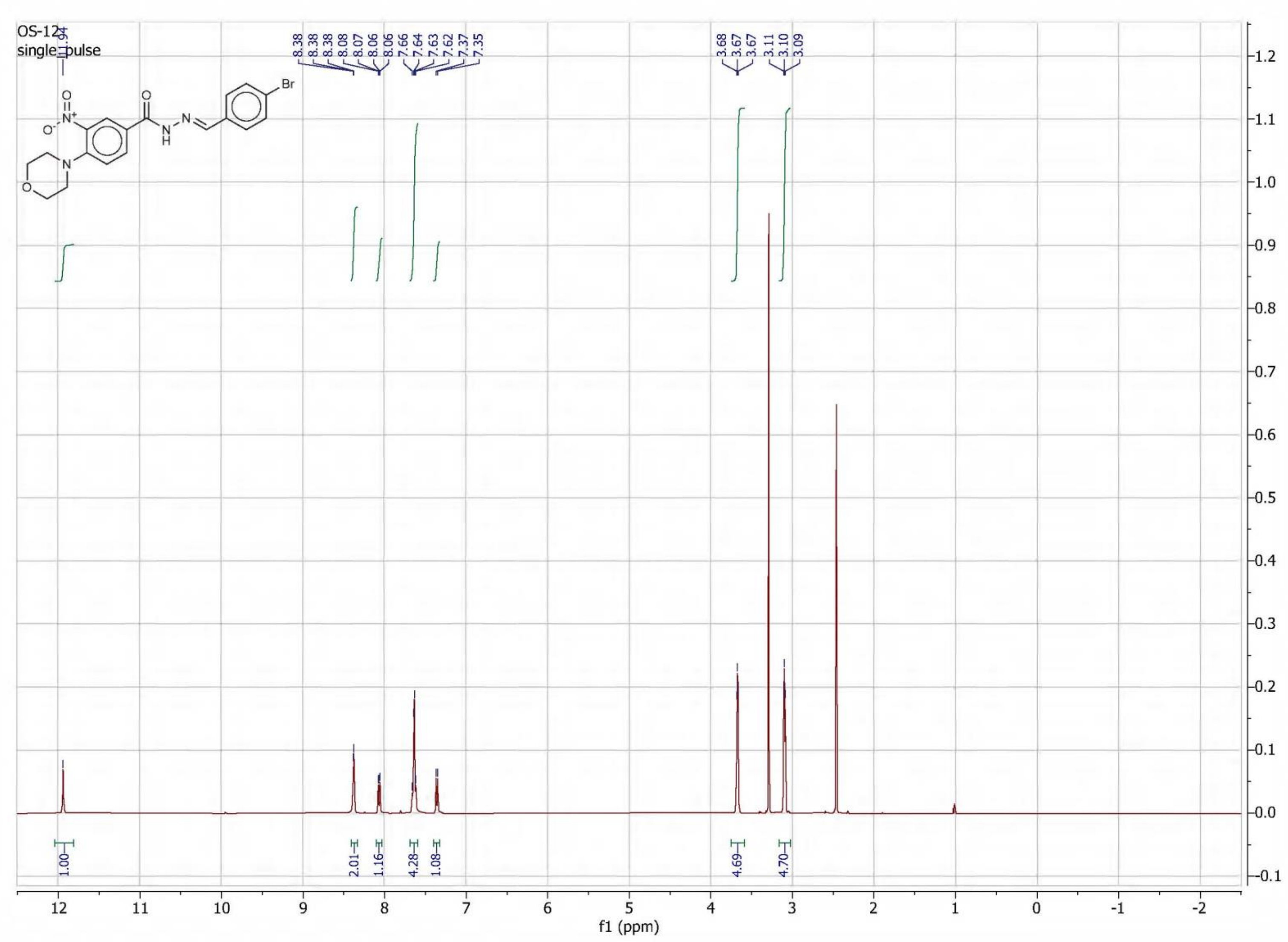

Figure S17. The ^{1}H NMR of compound **19**.

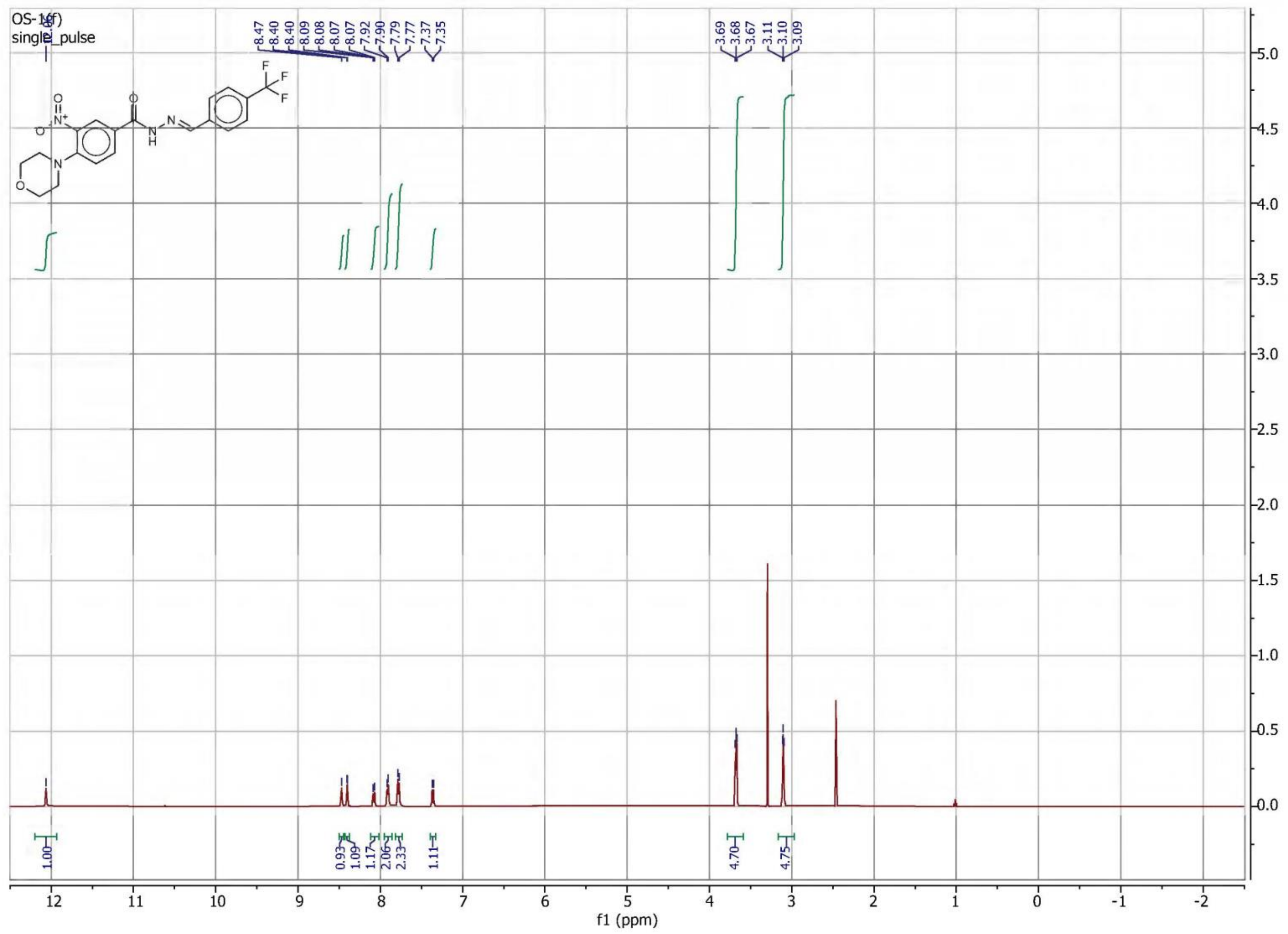

Figure S18. The ^{1}H NMR of compound **20**.

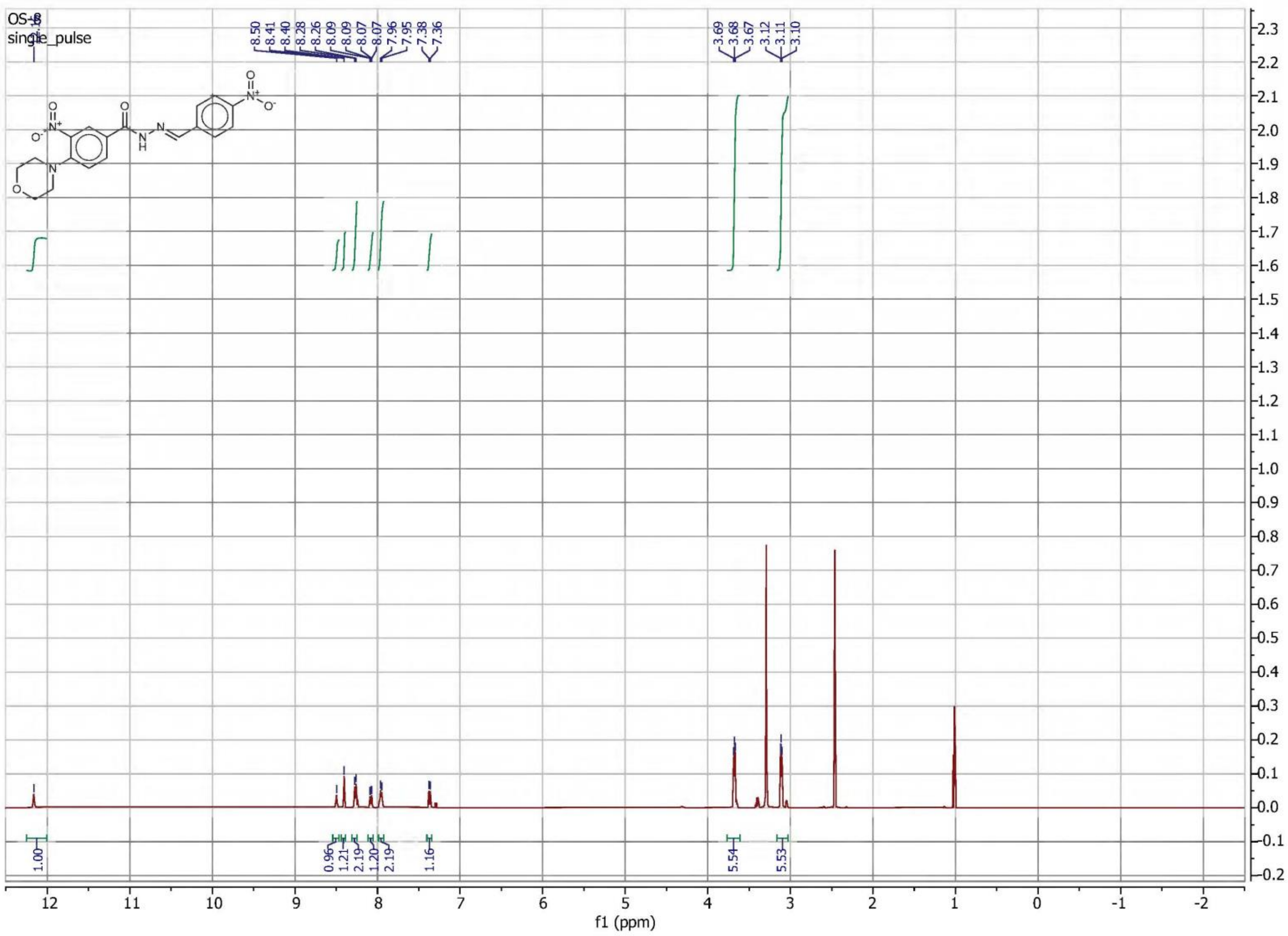

Figure S19. The ^{1}H NMR of compound **21**.

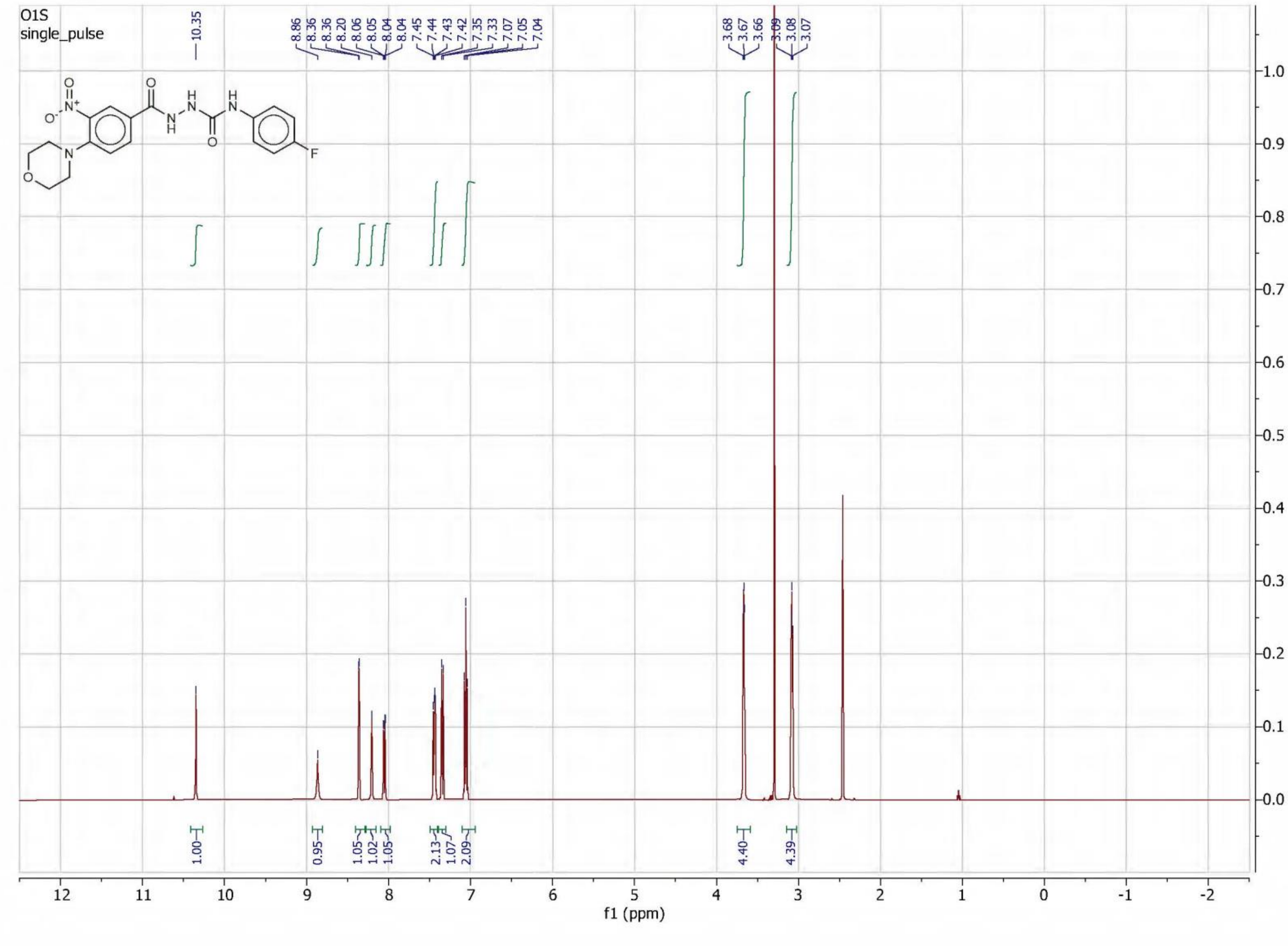

Figure S20. The ^{1}H NMR of compound **22**.

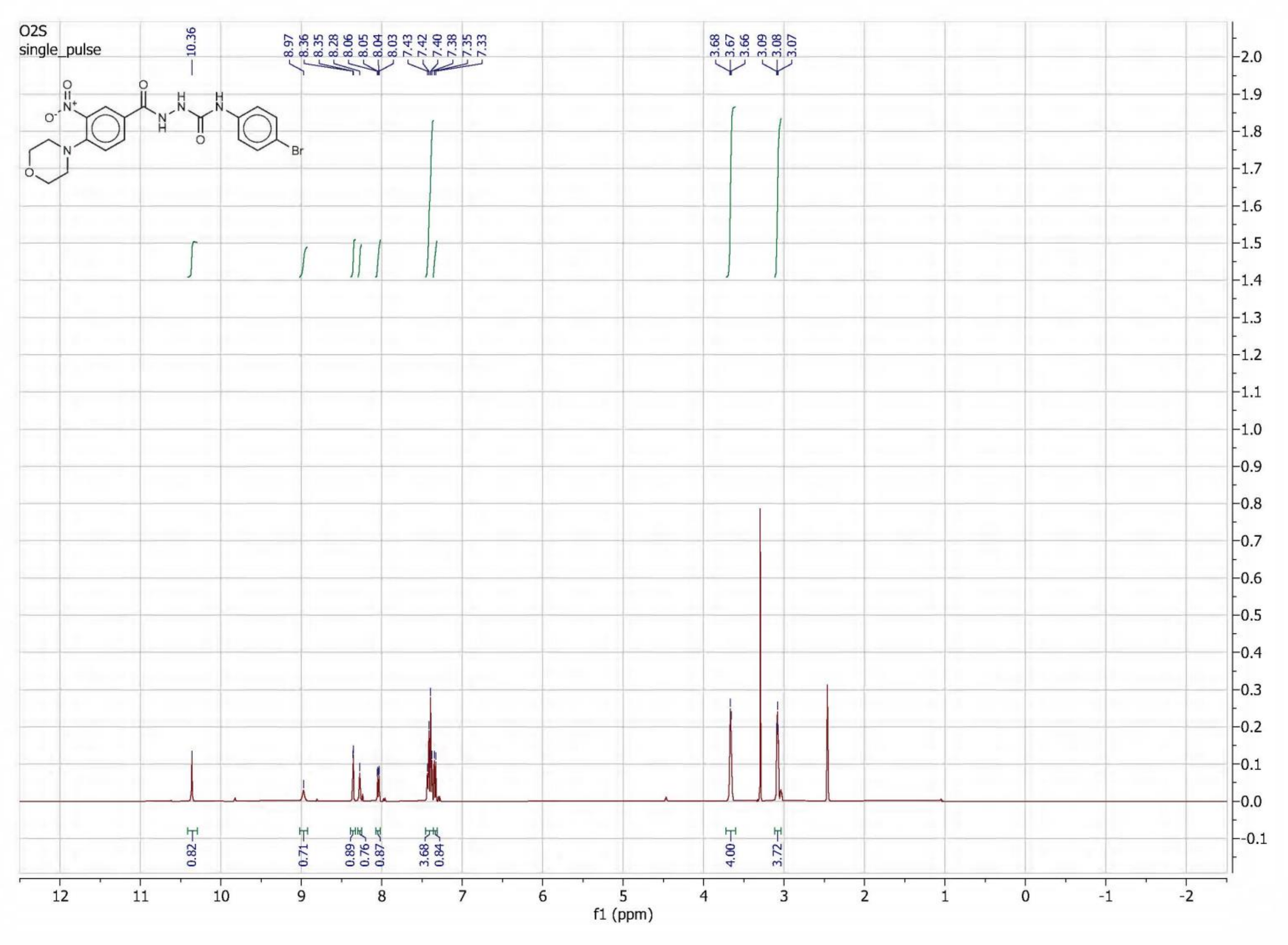

Figure S21. The ^{1}H NMR of compound **23**.

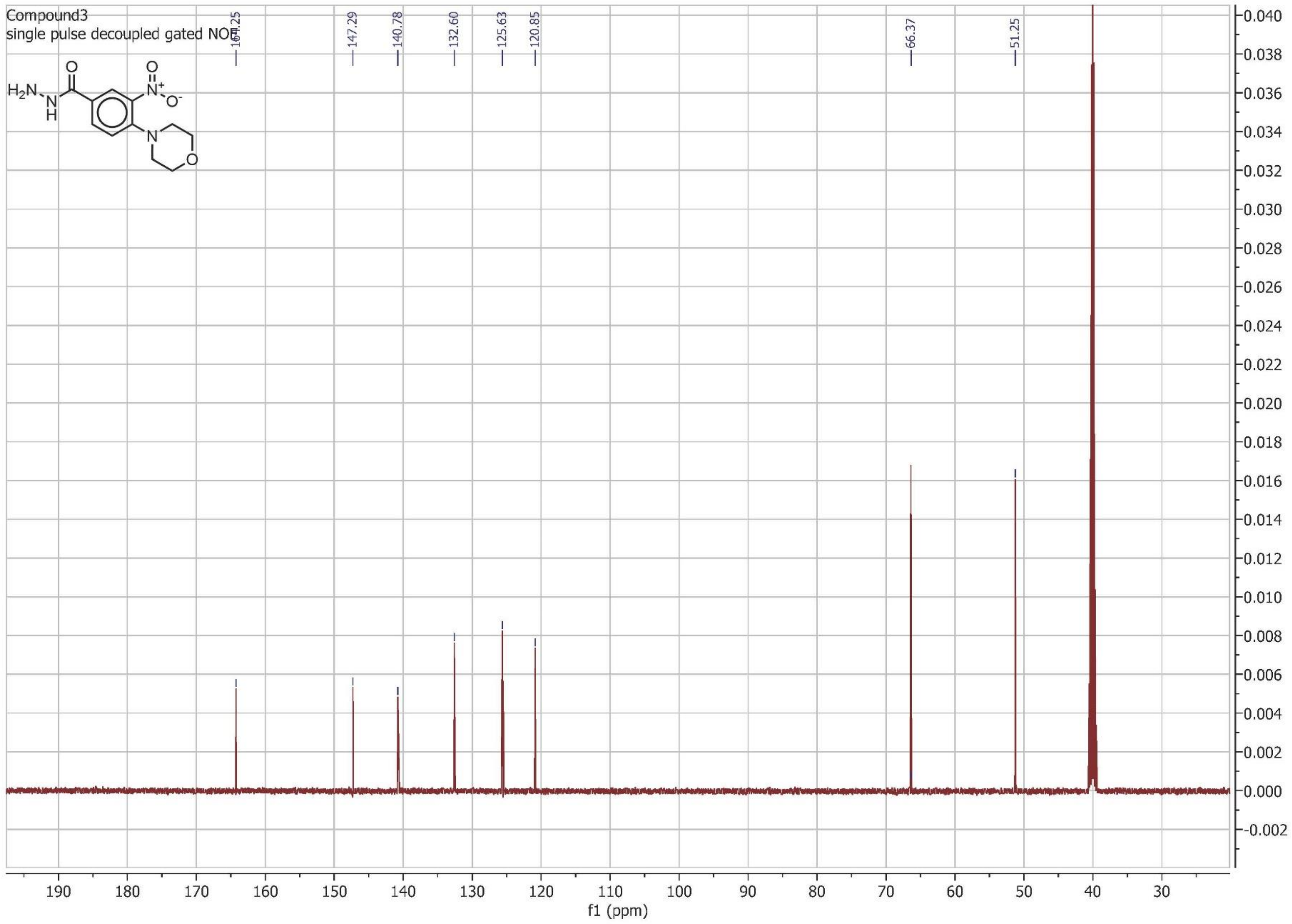

Figure S22. The ^{13}C NMR of compound **3**

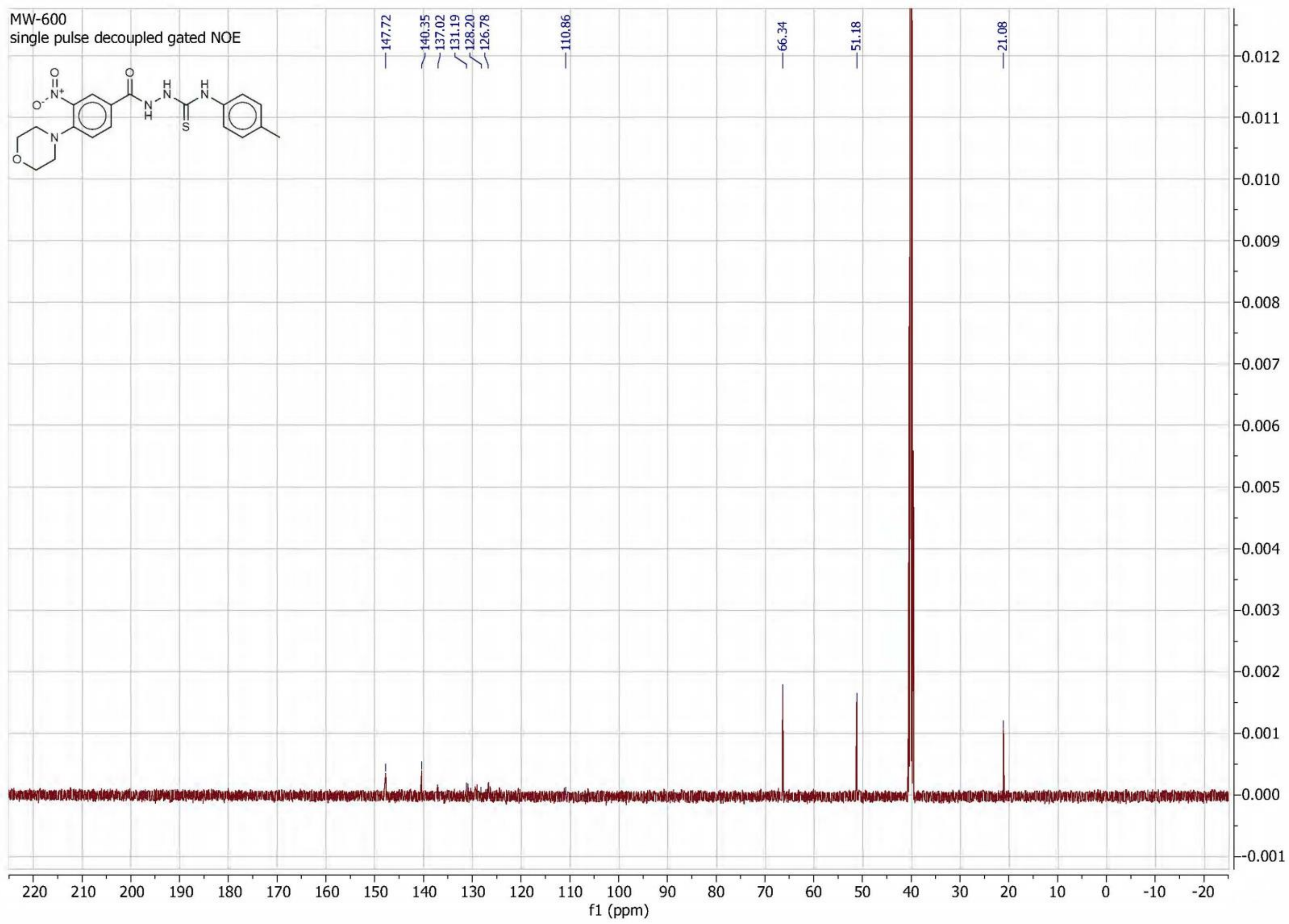

Figure S23. The ^{13}C NMR of compound **4**.

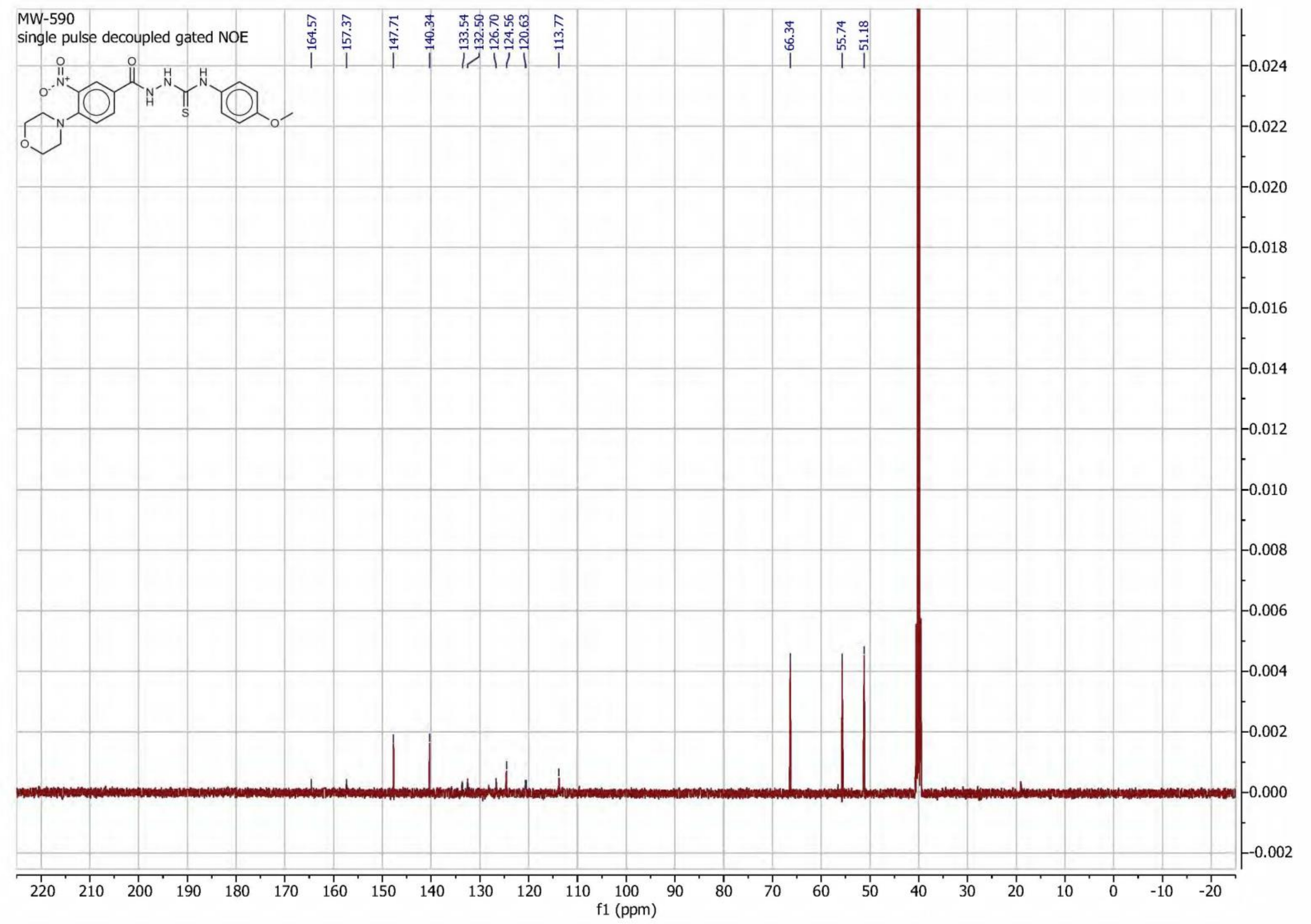

Figure S24. The ^{13}C NMR of compound **5**.

Figure S21. The ^{13}C NMR of compound 1

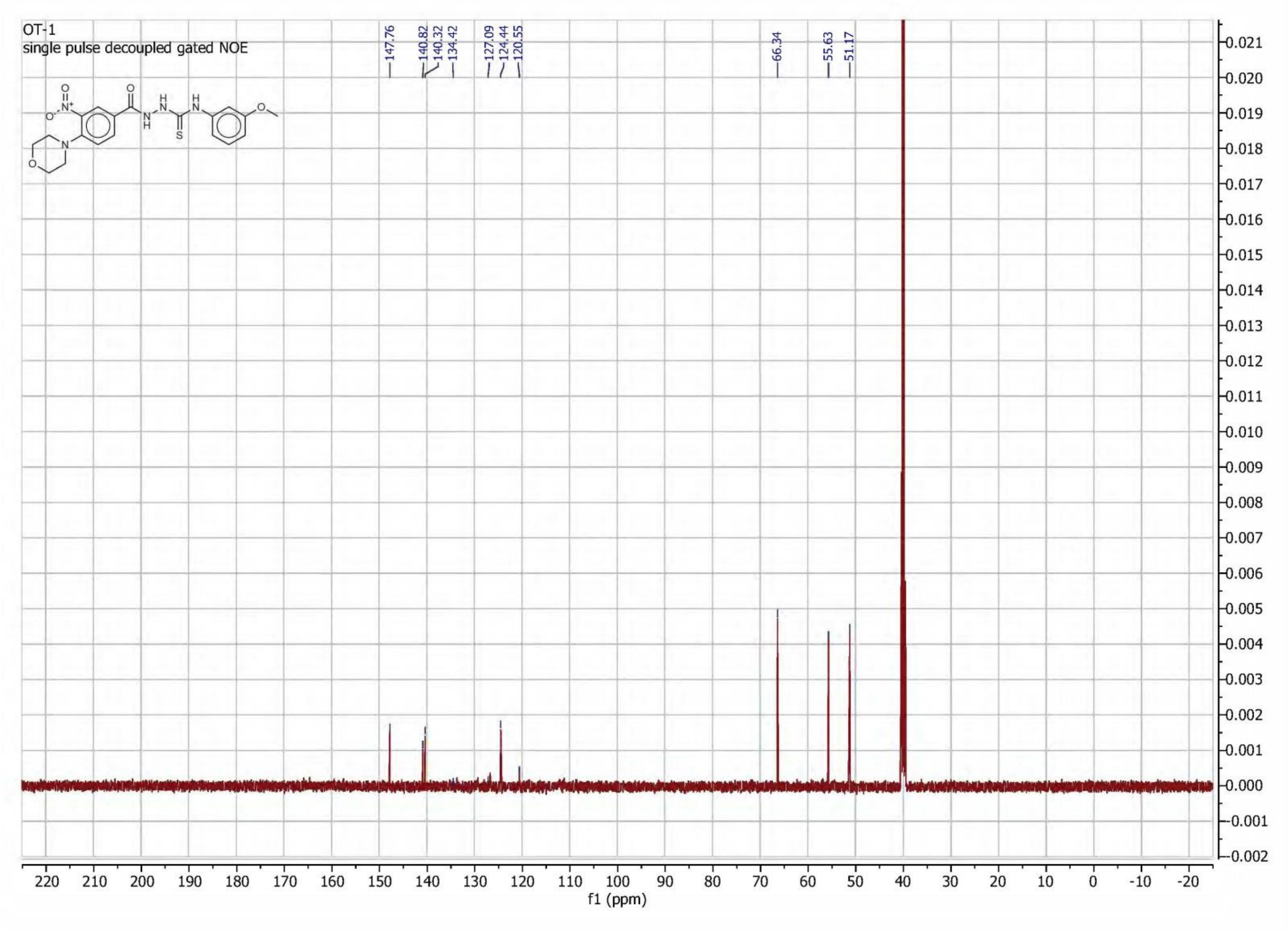

Figure S25. The ^{13}C NMR of compound **6**.

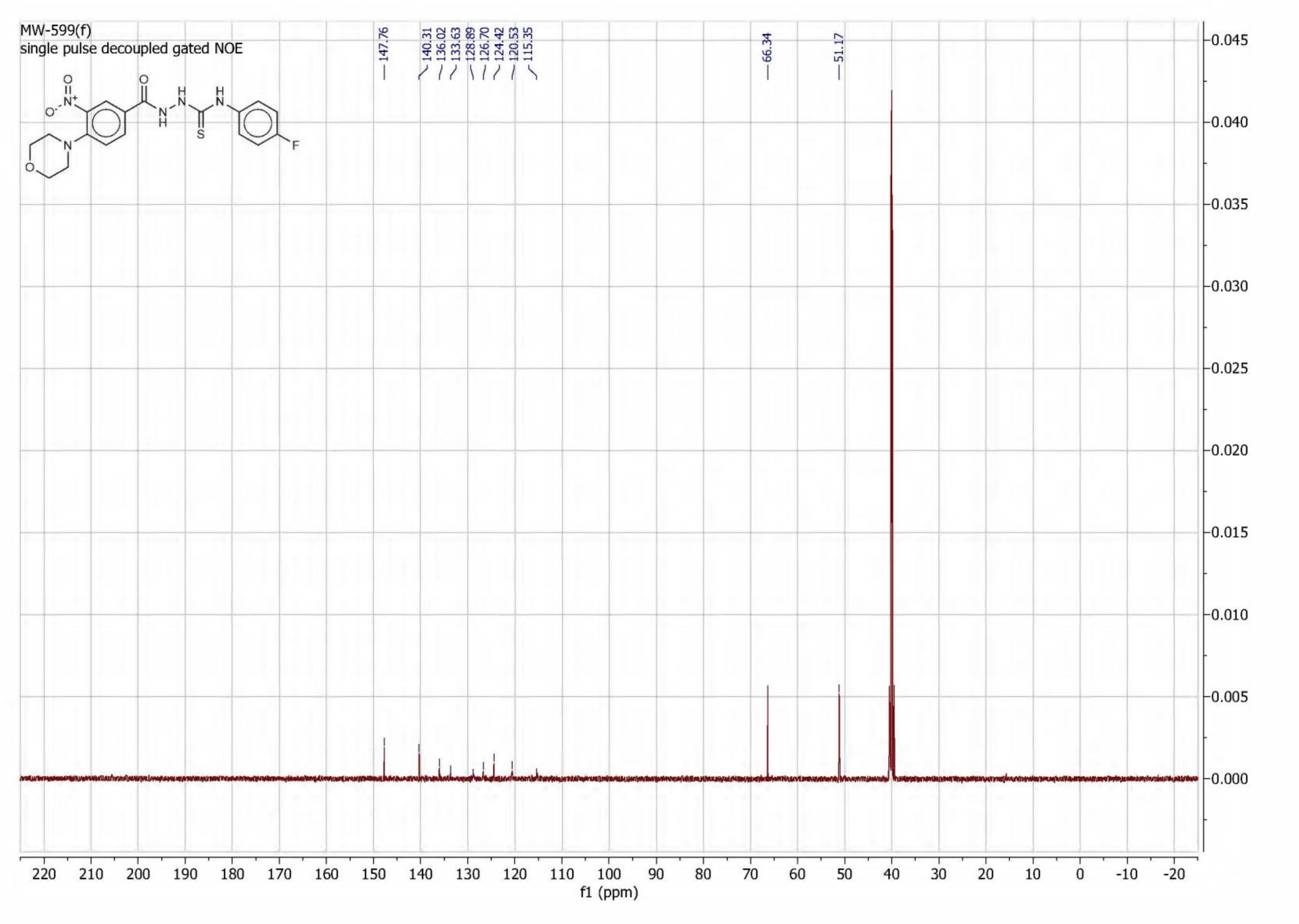

Figure S26. The ^{13}C NMR of compound **7**.

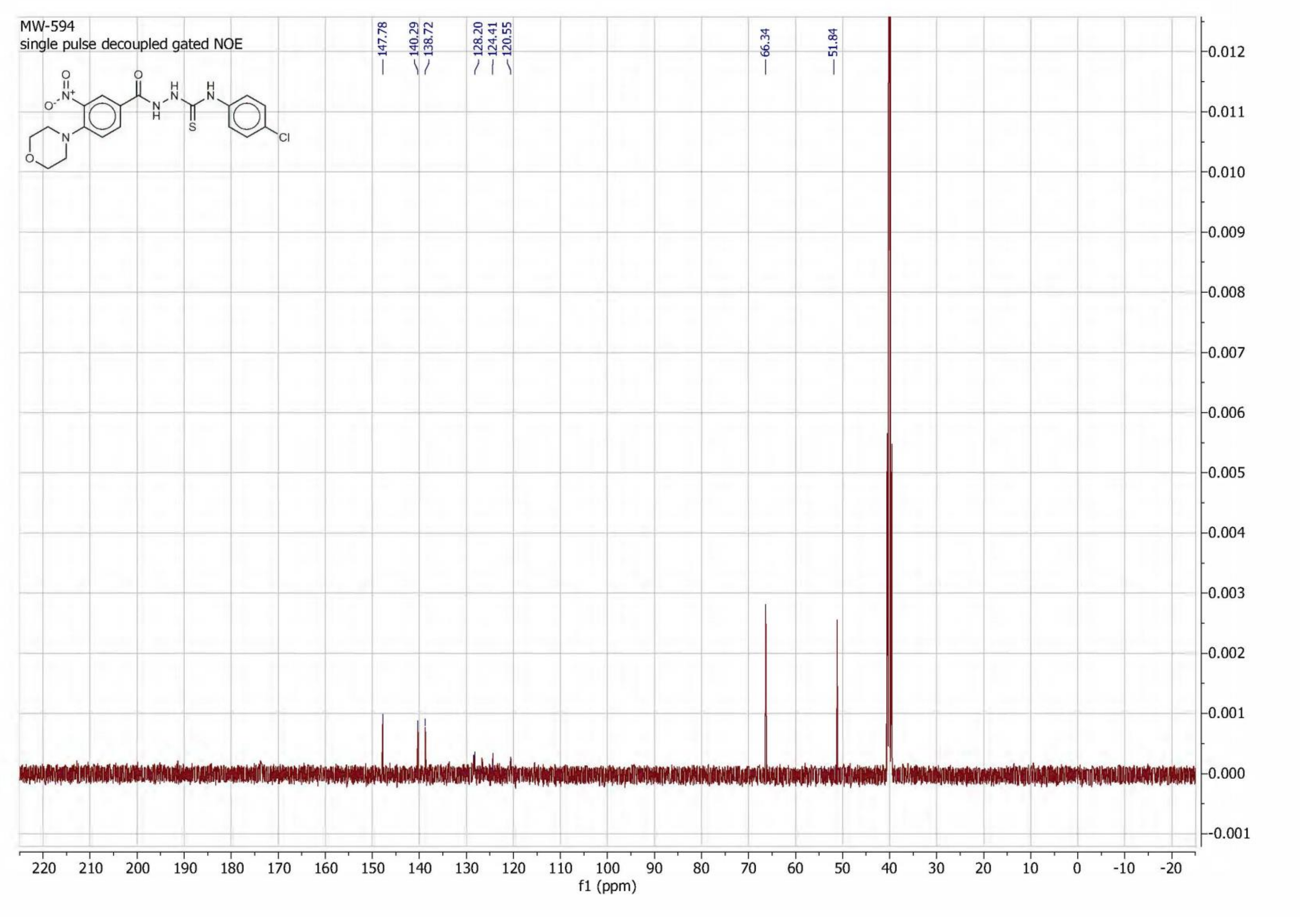

Figure S27. The ^{13}C NMR of compound **8**.

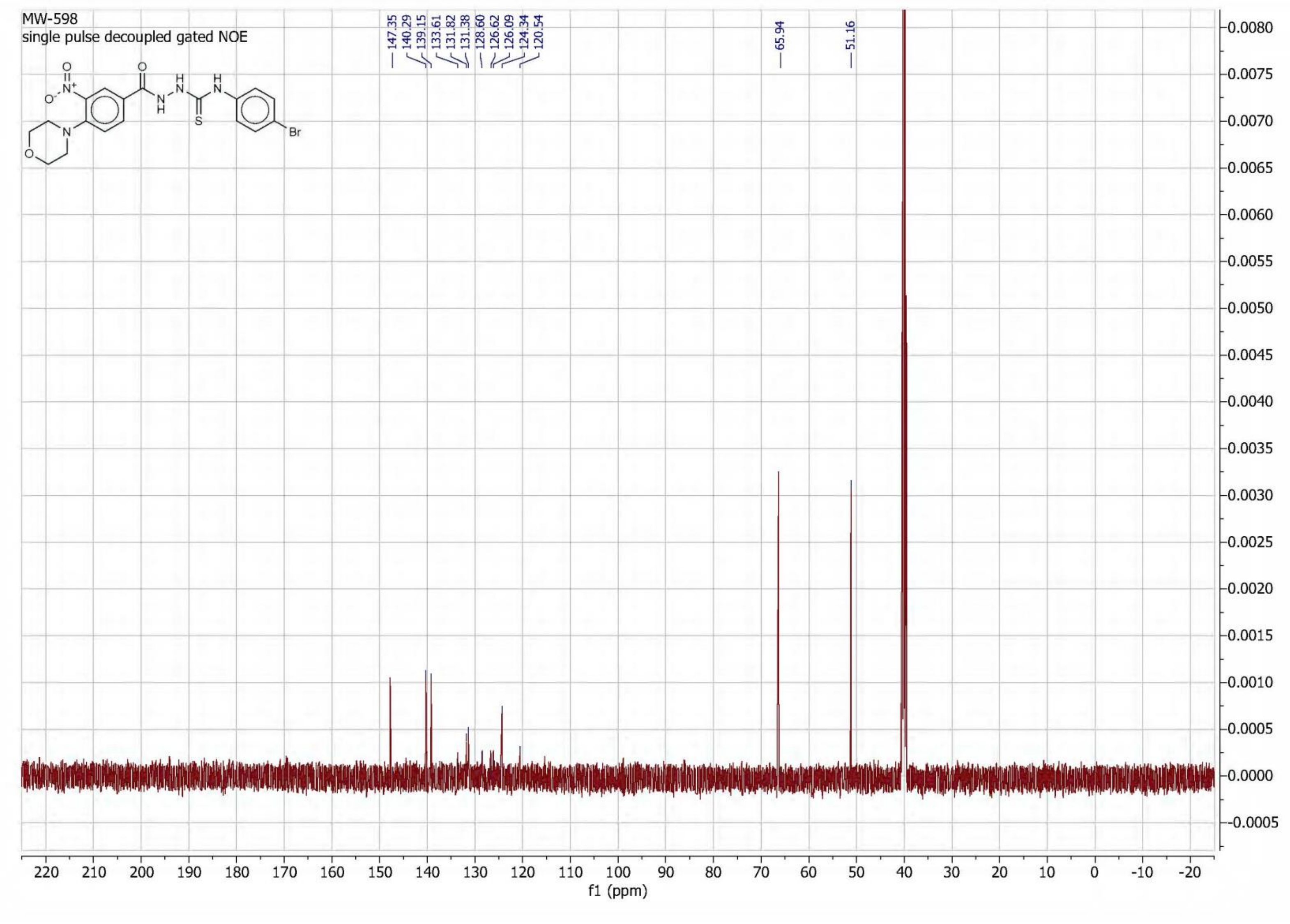

Figure S28. The ^{13}C NMR of compound **9**.

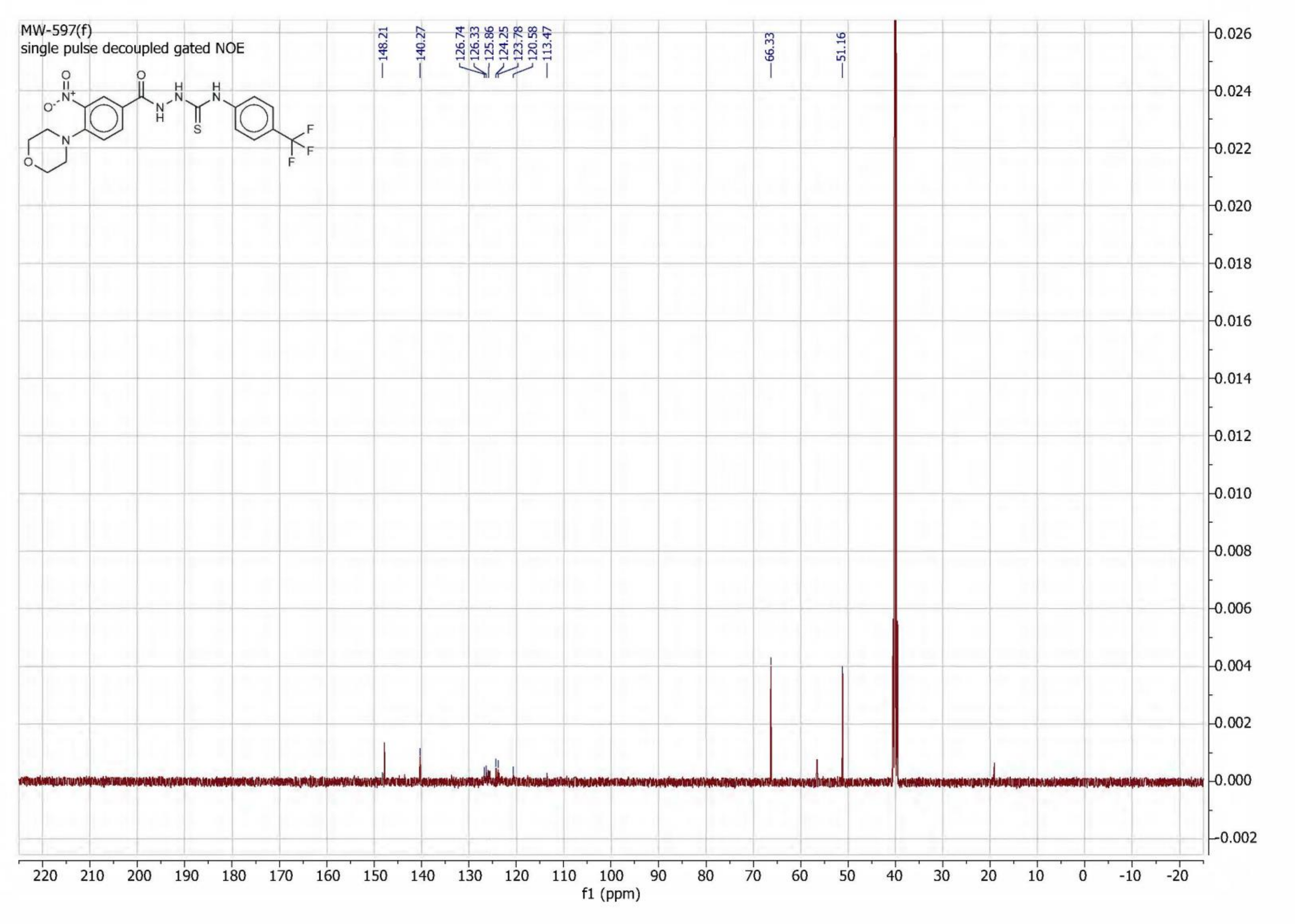

Figure S29. The ^{13}C NMR of compound **10**.

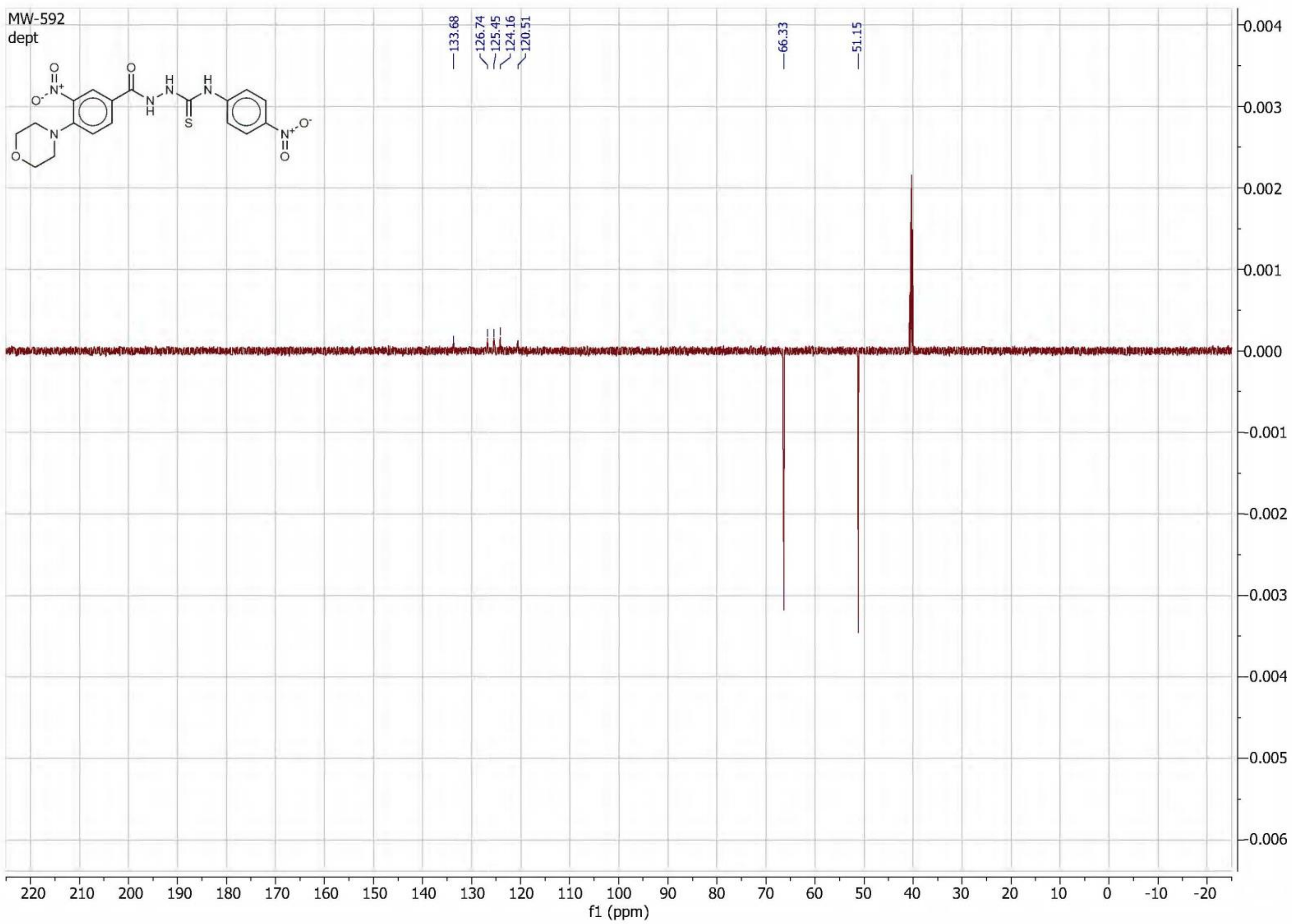

Figure S30. The ^{13}C NMR DEPT of compound **11**.

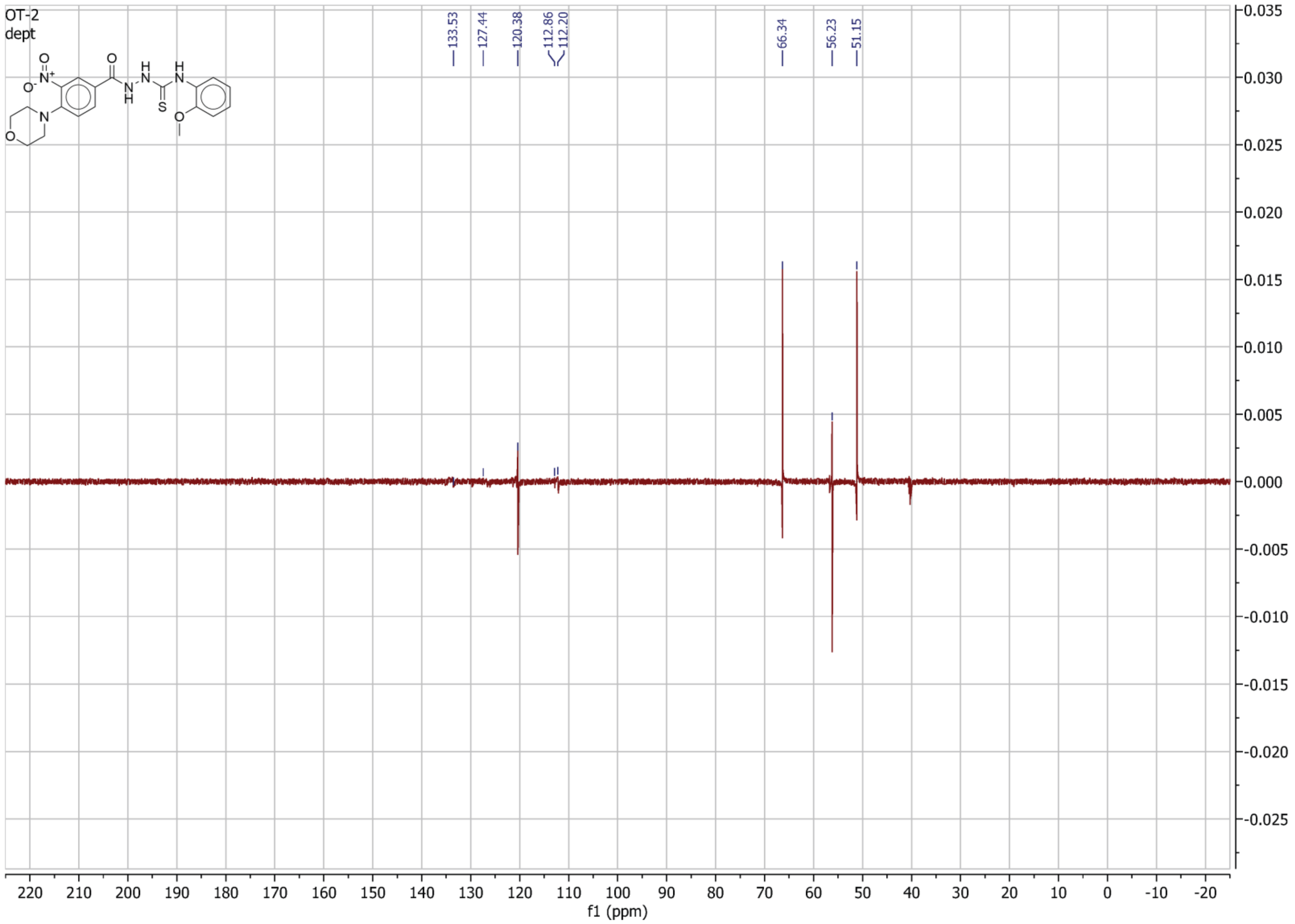

Figure S31. ^{13}C NMR DEPT of compound **12**.

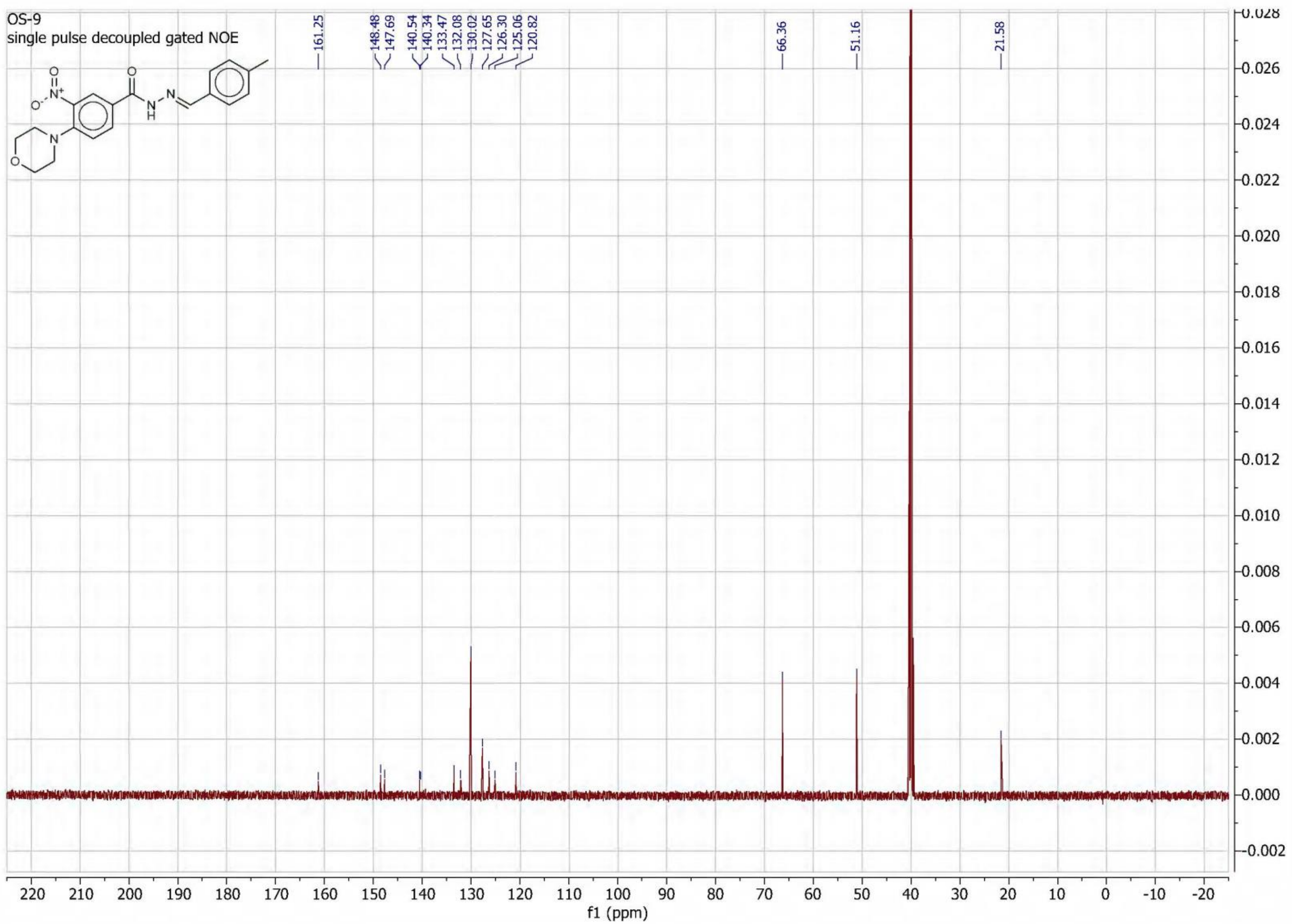

Figure S32. The ^{13}C NMR of compound **13**.

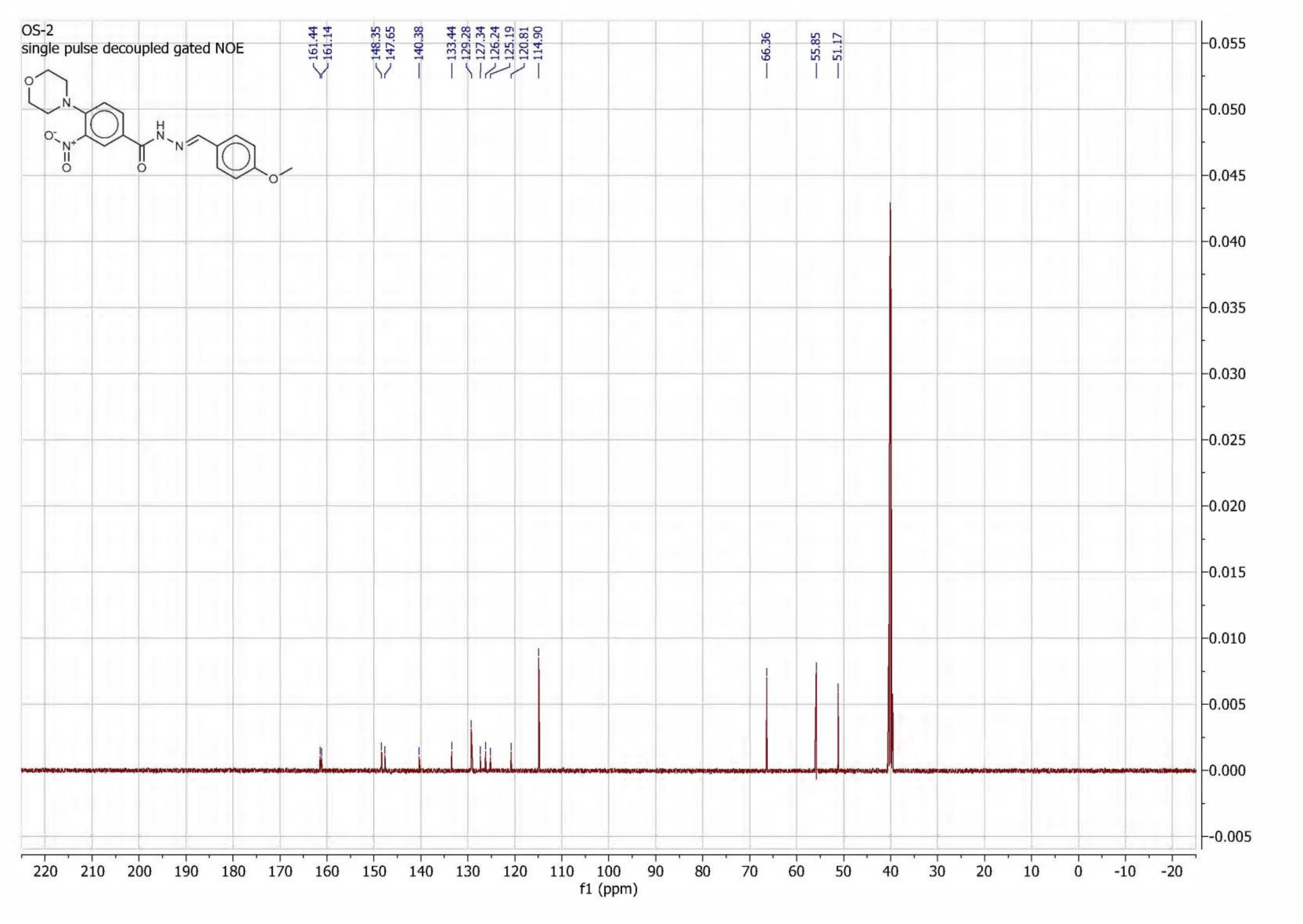

Figure S33. The ^{13}C NMR of compound **14**.

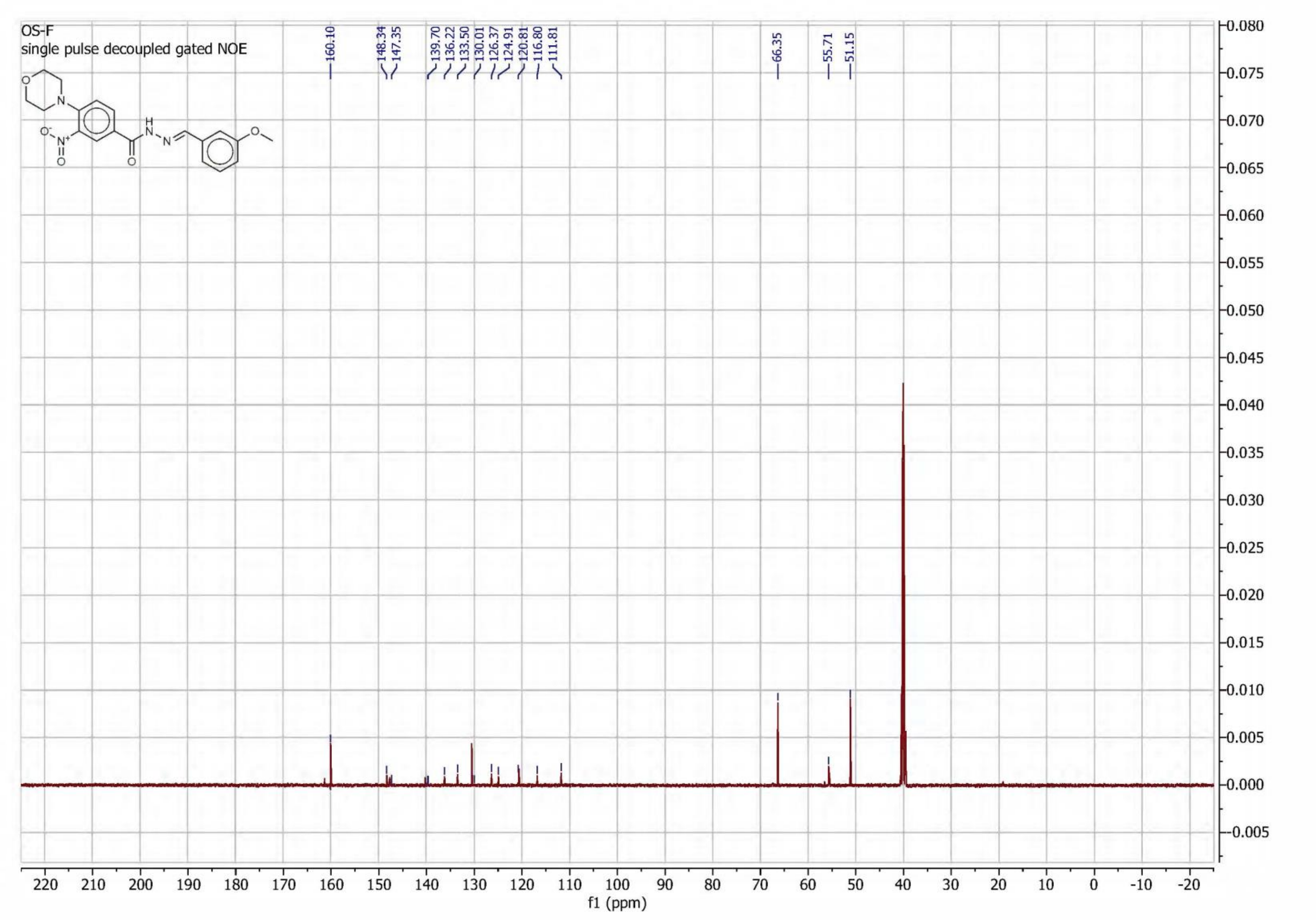

Figure S34. The ^{13}C NMR of compound **15**.

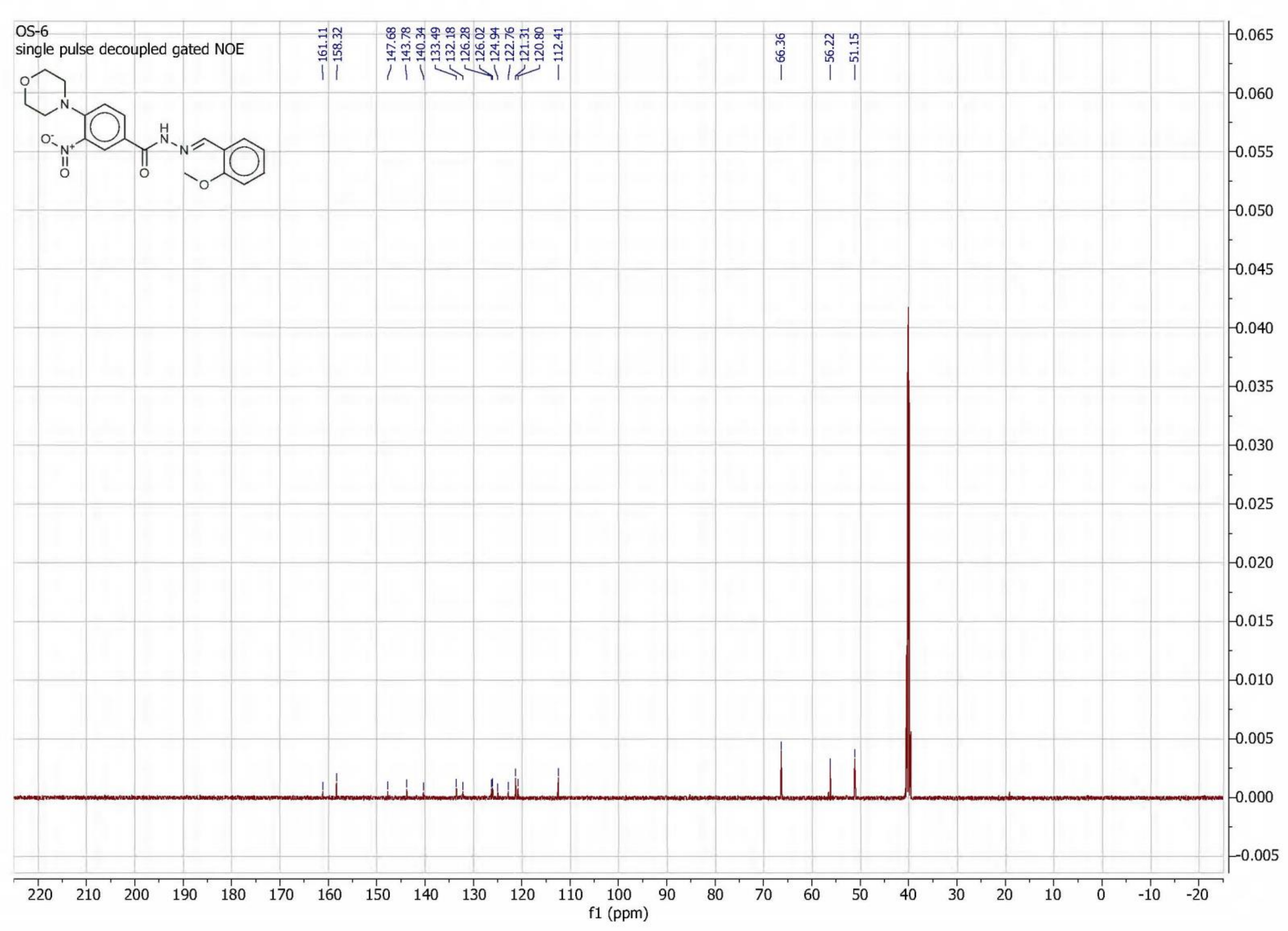

Figure S35. The ^{13}C NMR of compound **16**.

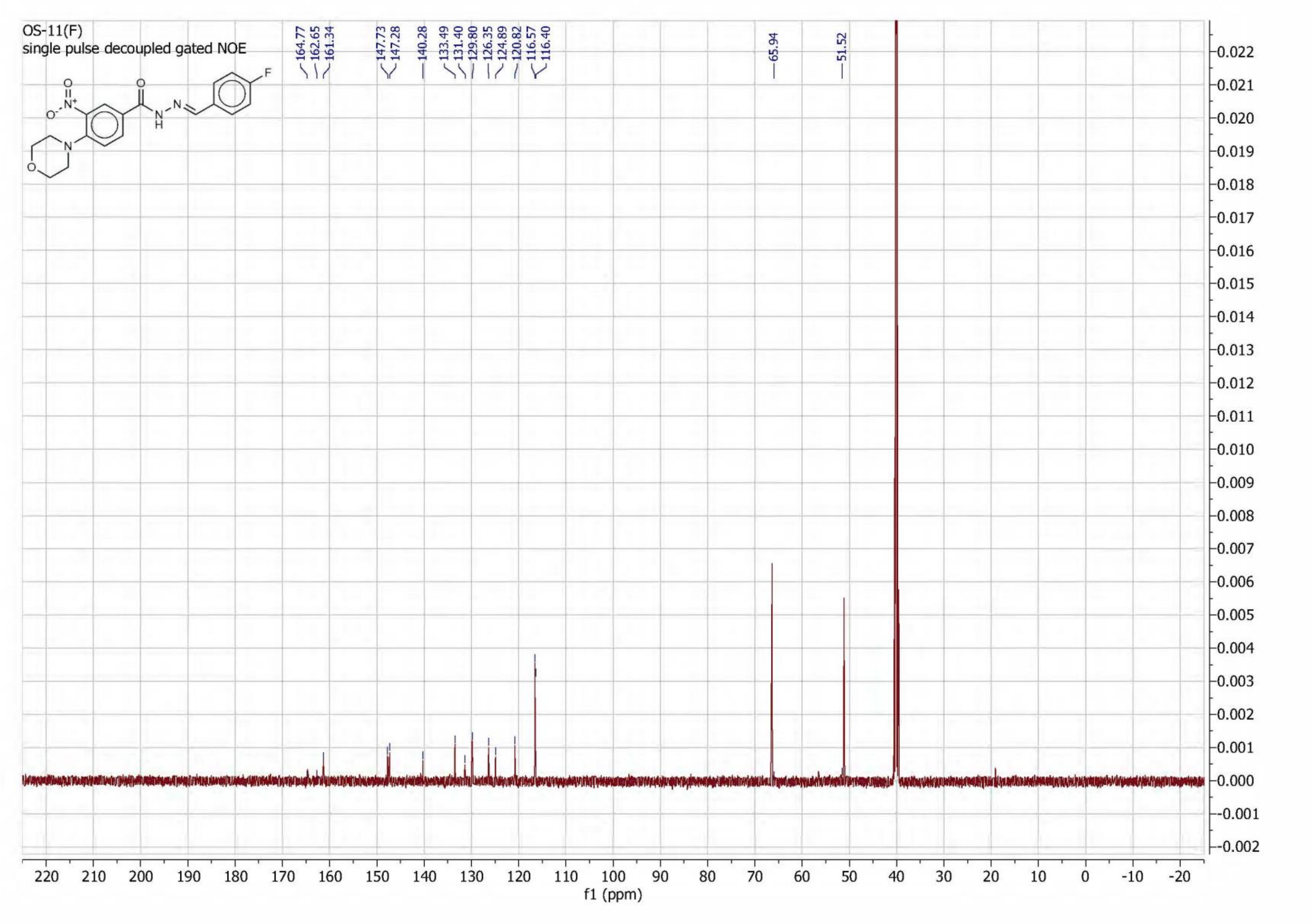

Figure S36. The ^{13}C NMR of compound **17**.

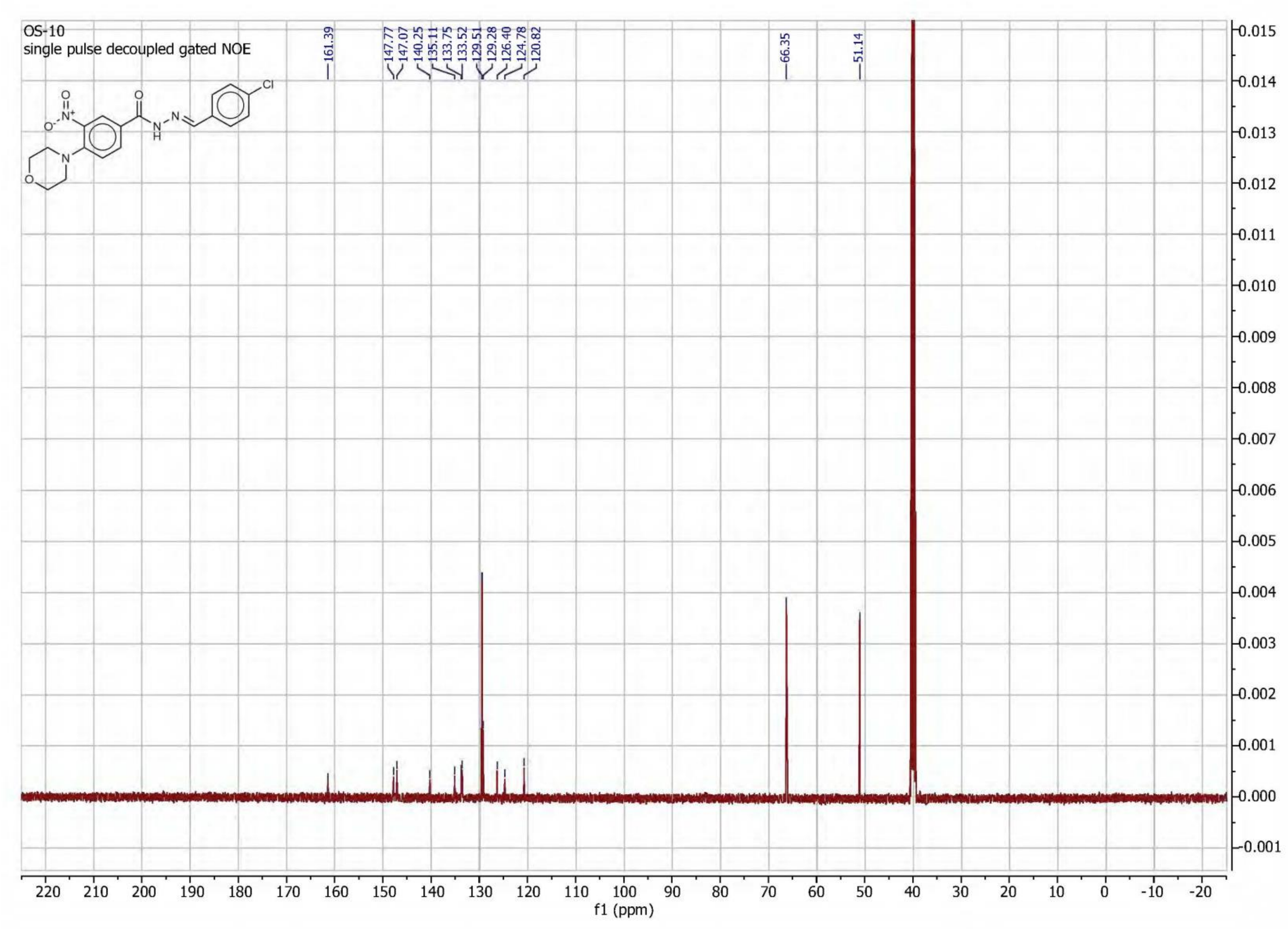

Figure S37. The ^{13}C NMR of compound **18**.

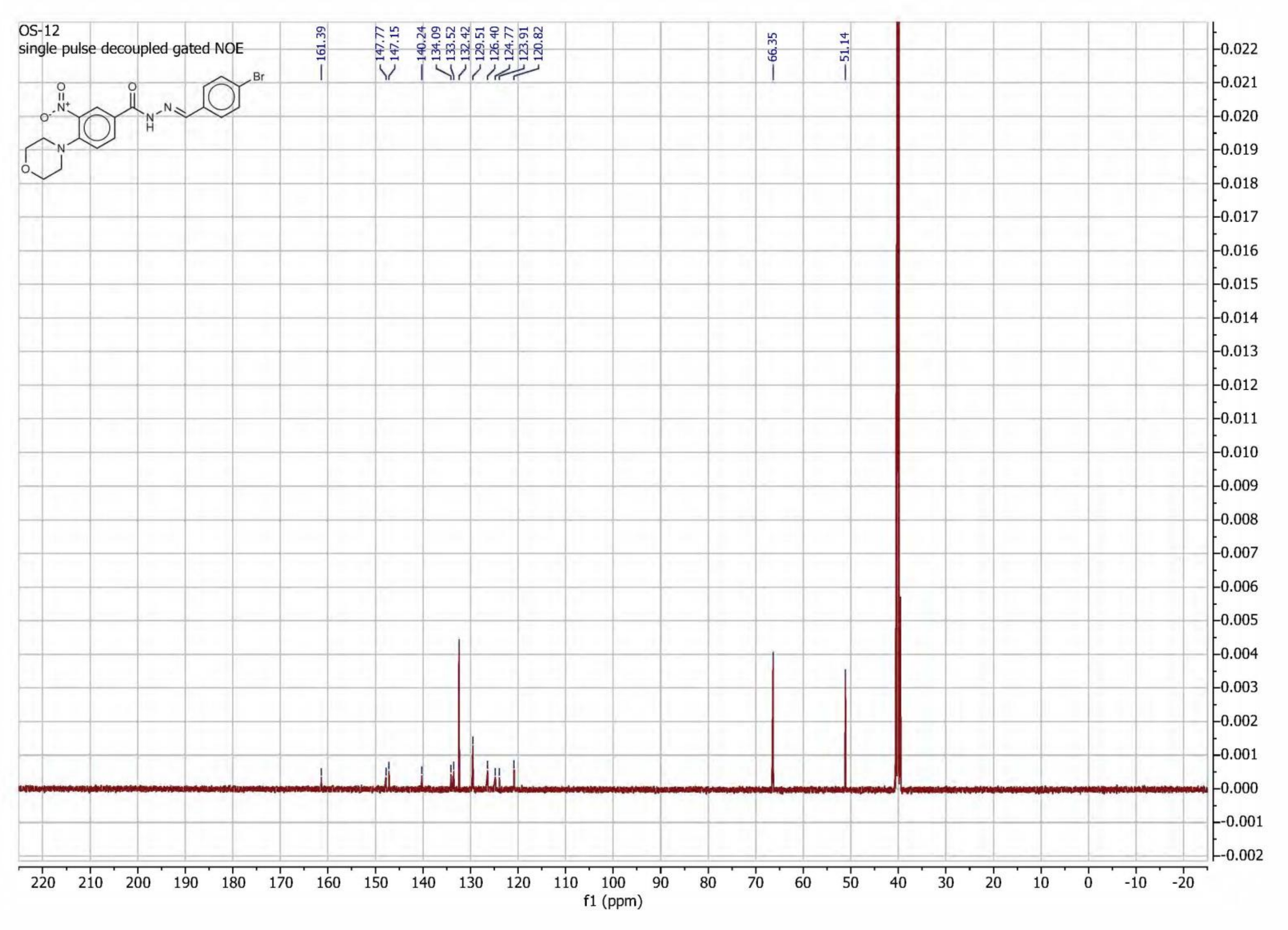

Figure S38. The ^{13}C NMR of compound **19**.

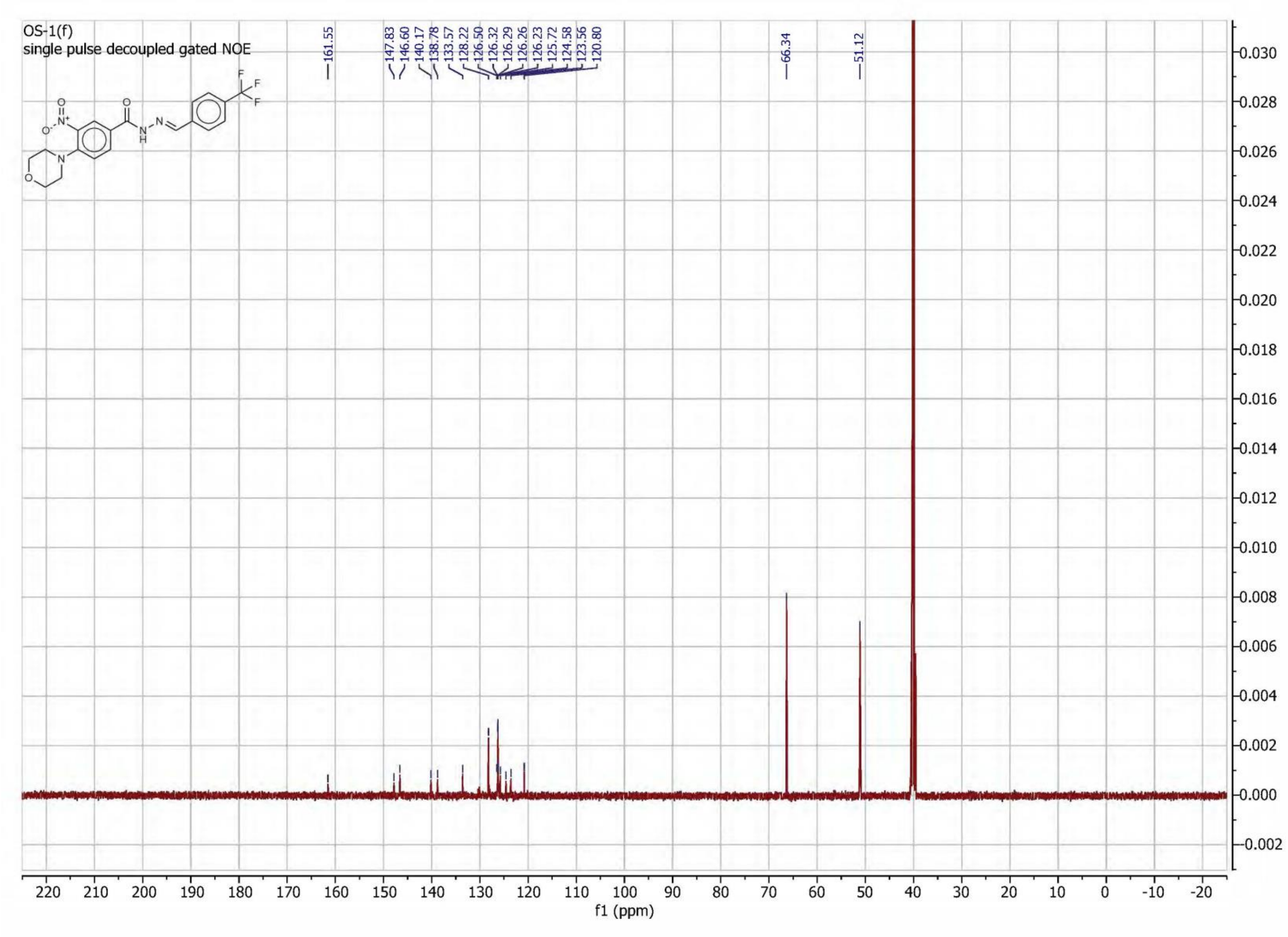

Figure S39. The ^{13}C NMR of compound **20**.

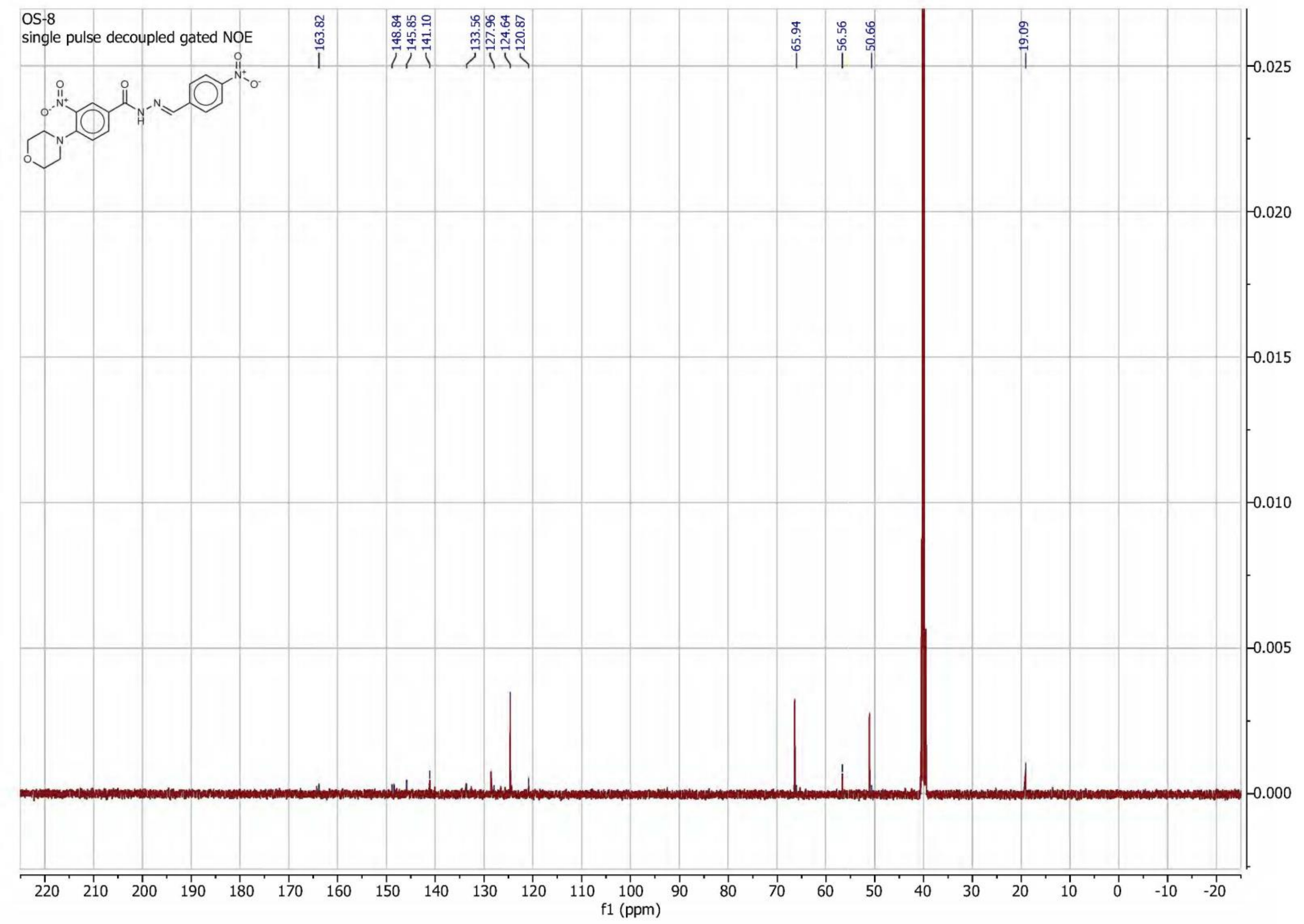

Figure S40. The ^{13}C NMR of compound **21**.

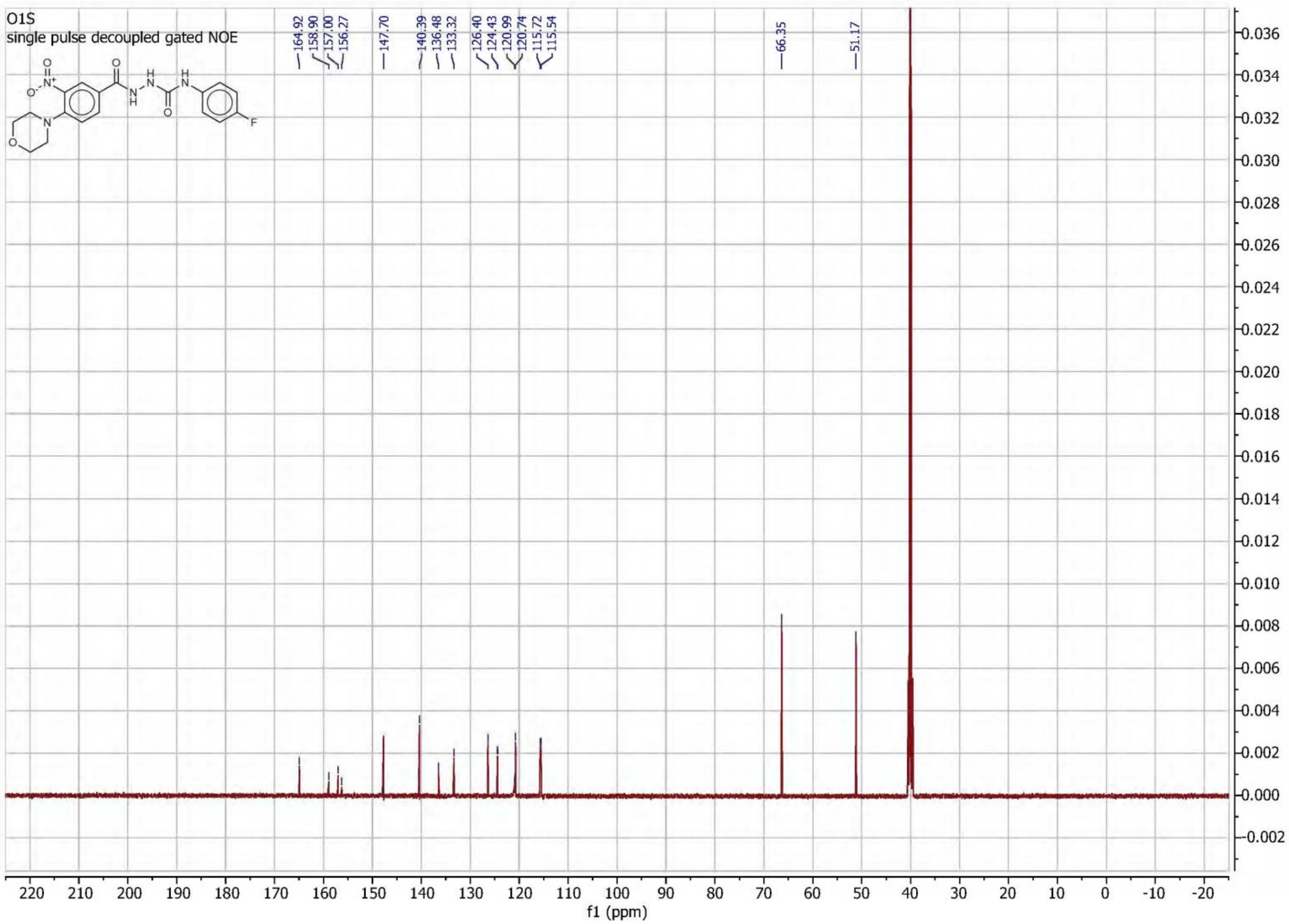

Figure S41. The ^{13}C NMR of compound **22**.

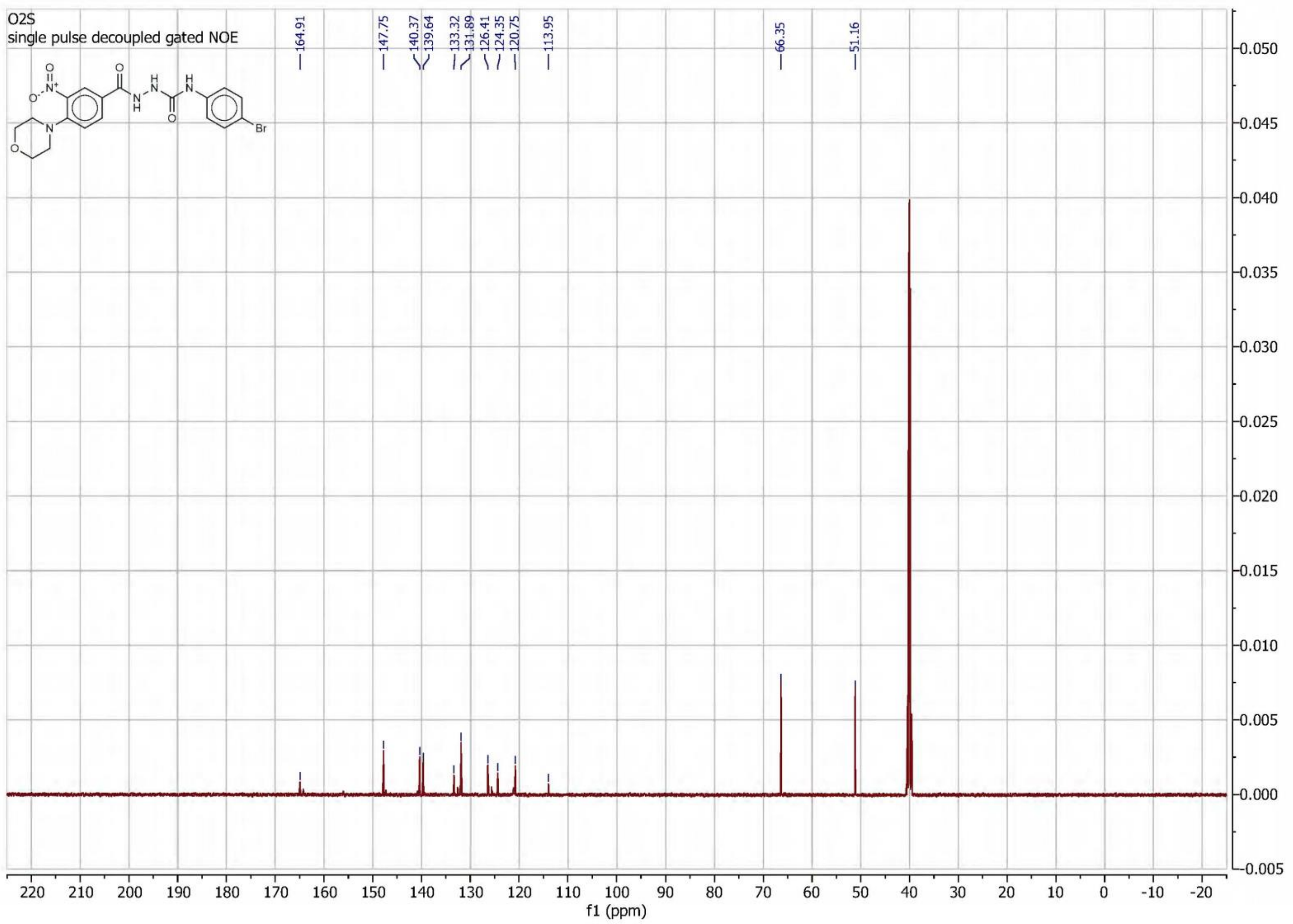

Figure S42. The ^{13}C NMR of compound **23**.

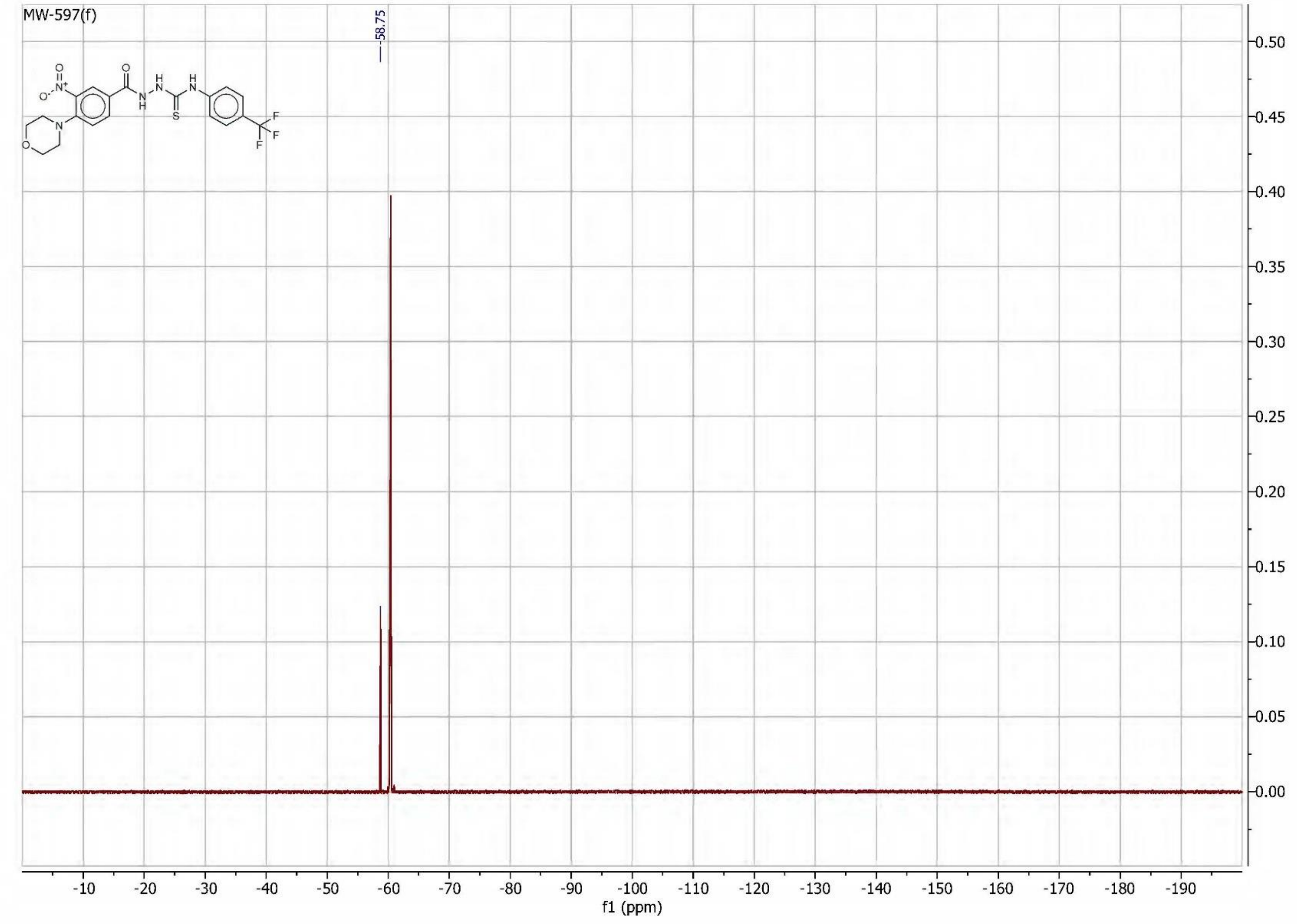

Figure S43. The ^{19}F NMR of compound **10**.

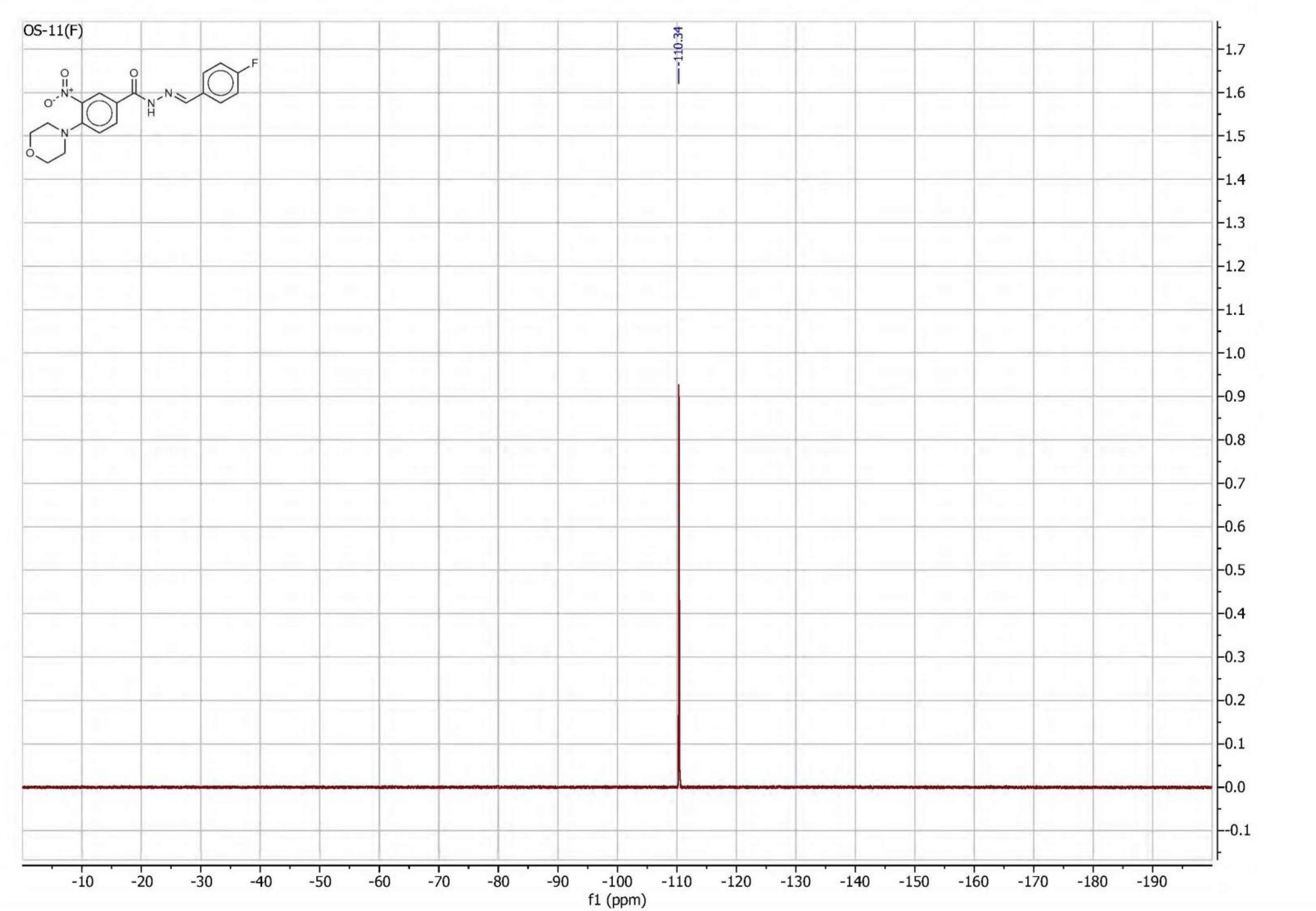

Figure S44. The ^{19}F NMR of compound **17**.

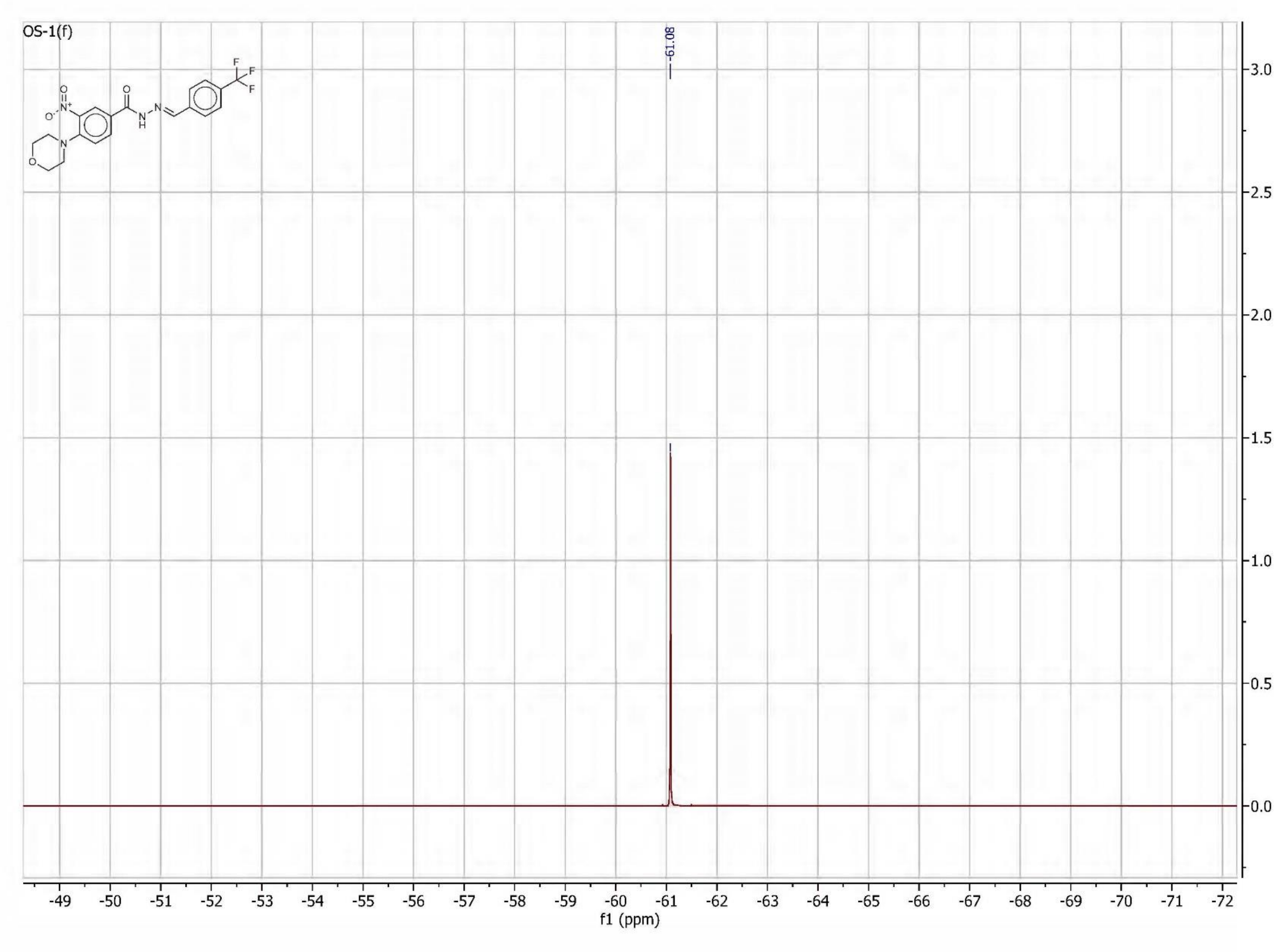

Figure S45. The ^{19}F NMR of compound **20**.

4. Supplementary crystallographic data for crystals **12**, **15** and **23**.

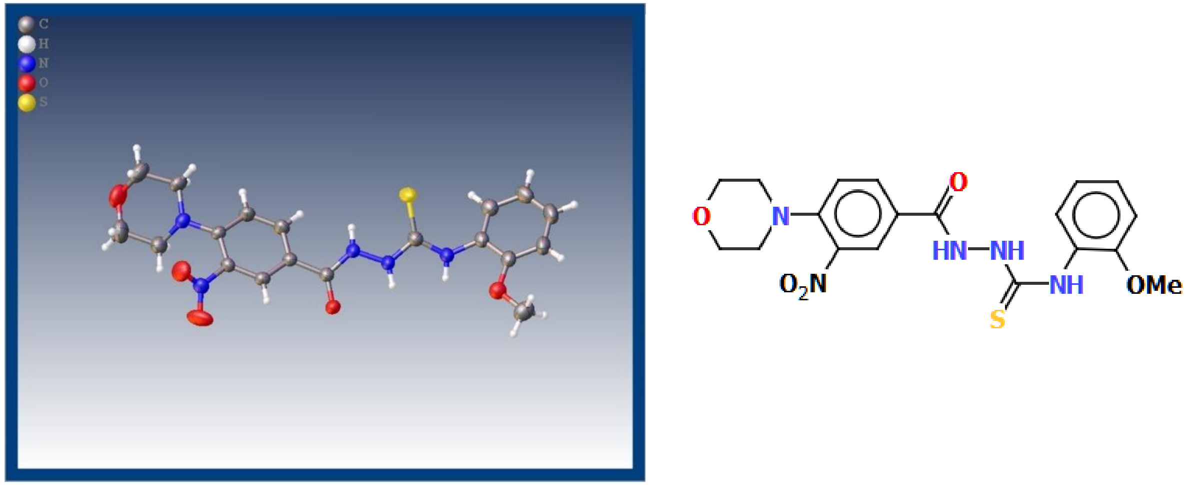

Figure S46. Molecular structure of **12**.

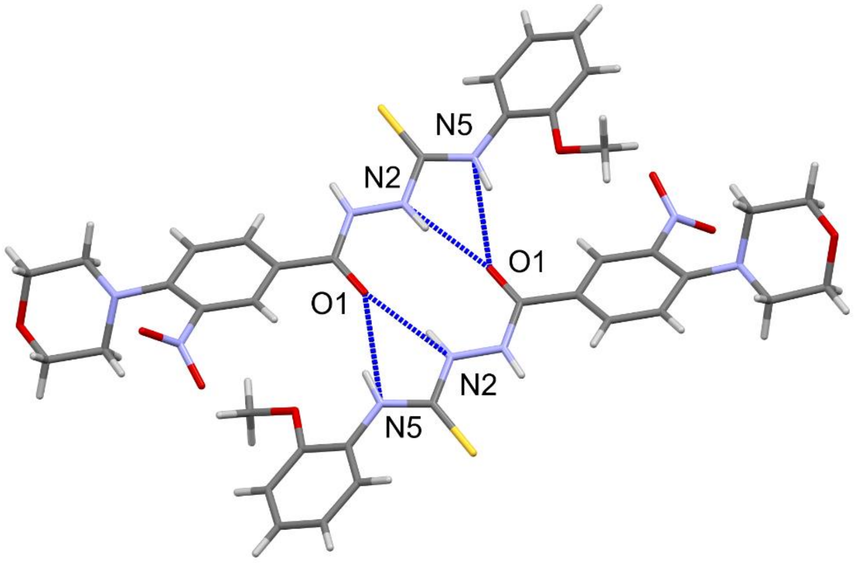

Figure S47. Centrosymmetric dimer in crystal of **12** formed by N2-H2…O1 and N5-H5…O1 hydrogen bonds.

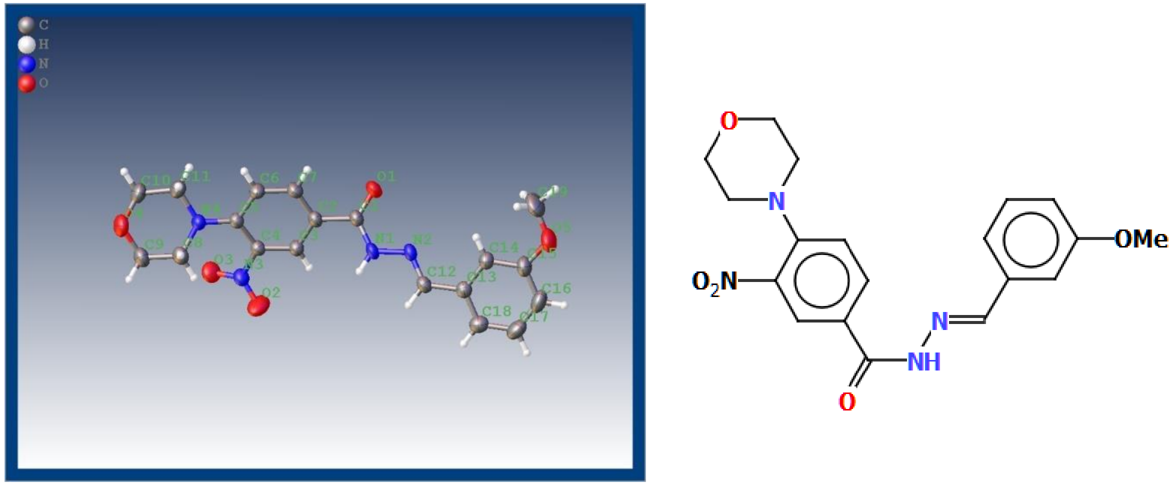

Figure S48. Molecular structure of **15**.

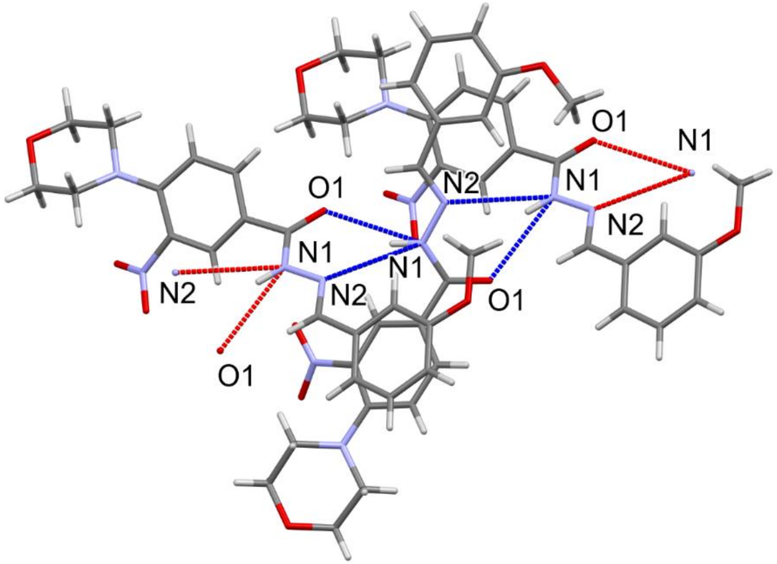

Figure S49. Bifurcated N1-H1…O1 and N1-H1…N2 hydrogen bonds in crystal of **15**.

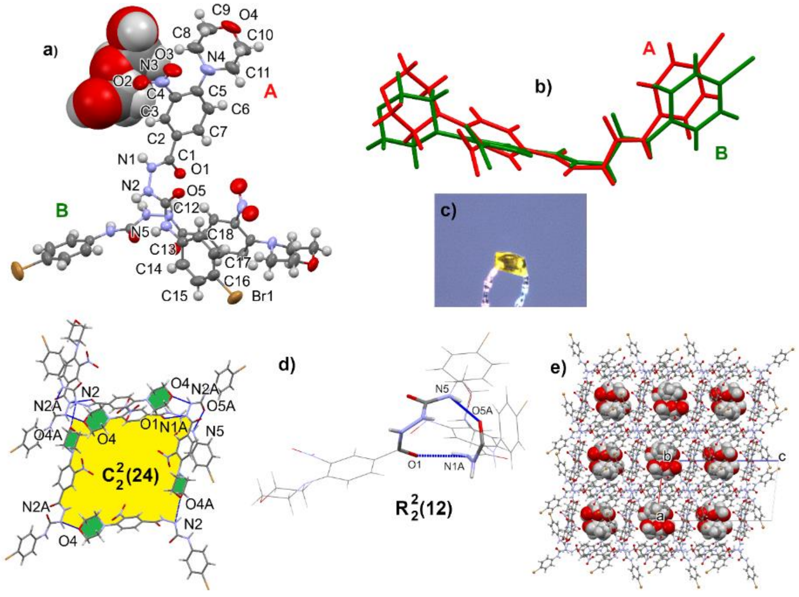

Figure S50. Molecular structure of **23**.

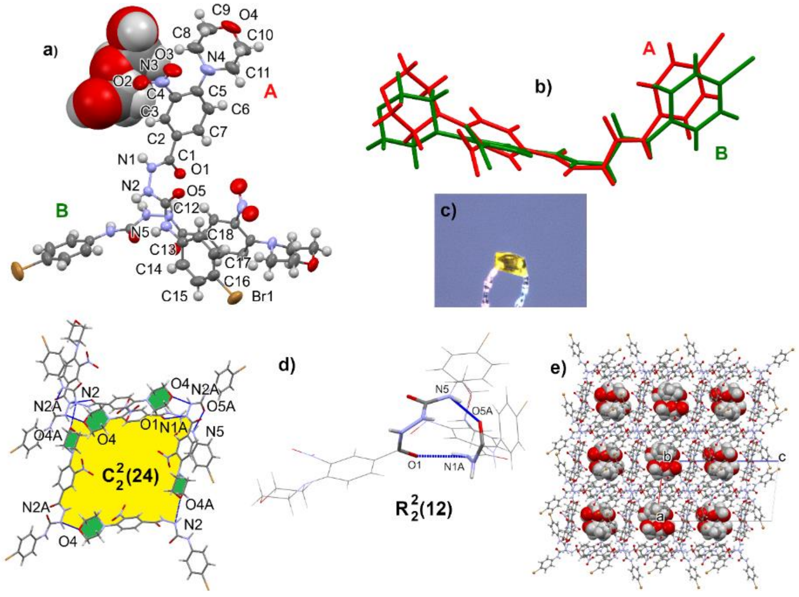

Figure S51. Bifurcated N1-H1…O1 and N1-H1…N2 hydrogen bonds in crystal of **23**.

Table S1 Crystal data and structure refinement for **12**, **15** and **23**.

Empirical formula (compound)	$C_{19}H_{21}N_5O_5S$ (**12**)	$C_{19}H_{20}N_4O_5$ (**15**)	$C_{37}H_{40}Br_2N_{10}O_{11}$ (**23**)
Formula weight	431.47	384.39	960.61
Temperature/K	293(2)	293(2)	293(2)
Crystal system	triclinic	monoclinic	monoclinic
Space group	*P*-1	$P2_1/c$	$P2_1/c$
a/Å	7.7989(7)	8.5020(2)	12.7298(2)
b/Å	10.6926(10)	26.7674(8)	14.8328(3)
c/Å	12.3918(9)	8.1607(2)	24.4071(4)
α/°	102.004(7)	90	90
β/°	94.181(7)	90.207(2)	96.6110(10)
γ/°	95.909(7)	90	90
Volume/Å^3	1000.66(15)	1857.17(8)	4577.87(14)
Z	2	4	4
ϱ_{calc}g/cm^3	1.432	1.375	1.394
μ/mm^{-1}	1.814	0.847	2.798
F(000)	452.0	808.0	1960.0
Crystal size/mm^3	0.25 × 0.2 × 0.04	0.45 × 0.15 × 0.08	0.3 × 0.3 × 0.18
Radiation	Cu Kα (λ = 1.54184)	Cu Kα (λ = 1.54184)	Cu Kα (λ = 1.54184)
2Θ range for data collection/°	7.328 to 152.118	10.404 to 151.95	6.986 to 152.34
Index ranges	-9 ≤ h ≤ 8, -13 ≤ k ≤ 13, -11 ≤ l ≤ 15	-10 ≤ h ≤ 9, -33 ≤ k ≤ 33, -10 ≤ l ≤ 9	-16 ≤ h ≤ 10, -18 ≤ k ≤ 18, -30 ≤ l ≤ 30
Reflections collected	6822	13220	33144
Independent reflections	4034 [R_{int} = 0.0328, R_{sigma} = 0.0584]	3820 [R_{int} = 0.0380, R_{sigma} = 0.0340]	9434 [R_{int} = 0.0424, R_{sigma} = 0.0358]
Data/restraints/parameters	4034/0/276	3820/0/258	9434/1/549
Goodness-of-fit on F^2	1.055	1.023	1.025
Final R indexes [I>=2σ (I)]	R_1 = 0.0539, wR_2 = 0.1262	R_1 = 0.0473, wR_2 = 0.1270	R_1 = 0.0714, wR_2 = 0.2269
Final R indexes [all data]	R_1 = 0.0805, wR_2 = 0.1478	R_1 = 0.0606, wR_2 = 0.1407	R_1 = 0.0858, wR_2 = 0.2468
Largest diff. peak/hole / e Å^{-3}	0.31/-0.35	0.29/-0.22	1.76/-0.86
CCDC No.	2445821	2445822	2447754

Table S2 Bond Lengths for **12**, **15** and **23**.

**12**			**15**			**23 A**			**23 B**		
**Atom**	**Atom**	**Length/Å**	**Atom**	**Atom**	**Length/Å**	**Atom**	**Atom**	**Length/Å**	**Atom**	**Atom**	**Length/Å**
S1	C12	1.664(3)	O1	C1	1.226(2)	Br1	C16	1.903(4)	Br1A	C16A	1.902(4)
O1	C1	1.234(3)	O2	N3	1.214(2)	O1	C1	1.226(4)	O1A	C1A	1.237(4)
O2	N3	1.218(3)	O3	N3	1.2121(19)	O2	N3	1.235(6)	O2A	N3A	1.215(6)
O3	N3	1.207(3)	O4	C9	1.420(3)	O3	N3	1.217(6)	O3A	N3A	1.217(5)
O4	C9	1.409(5)	O4	C10	1.418(3)	O4	C9	1.419(9)	O4A	C9A	1.423(6)
O4	C10	1.423(5)	O5	C15	1.356(2)	O4	C10	1.418(8)	O4A	C10A	1.404(6)
O5	C14	1.361(4)	O5	C19	1.413(3)	O5	C12	1.229(4)	O5A	C12A	1.232(4)
O5	C19	1.426(3)	N1	N2	1.3808(18)	N1	N2	1.386(4)	N1A	N2A	1.382(4)
N1	N2	1.382(3)	N1	C1	1.342(2)	N1	C1	1.360(4)	N1A	C1A	1.347(4)
N1	C1	1.333(3)	N2	C12	1.269(2)	N2	C12	1.373(4)	N2A	C12A	1.384(4)
N2	C12	1.360(3)	N3	C4	1.468(2)	N3	C4	1.475(5)	N3A	C4A	1.453(4)
N3	C4	1.474(3)	N4	C5	1.380(2)	N4	C5	1.390(5)	N4A	C5A	1.415(4)
N4	C5	1.412(3)	N4	C8	1.468(2)	N4	C8	1.475(6)	N4A	C8A	1.468(5)
N4	C8	1.473(4)	N4	C11	1.460(2)	N4	C11	1.455(7)	N4A	C11A	1.465(5)
N4	C11	1.465(3)	C1	C2	1.493(2)	N5	C12	1.351(4)	N5A	C12A	1.343(4)
N5	C12	1.343(3)	C2	C3	1.387(2)	N5	C13	1.412(4)	N5A	C13A	1.408(4)
N5	C13	1.422(3)	C2	C7	1.387(2)	C1	C2	1.485(4)	C1A	C2A	1.485(4)
C1	C2	1.487(3)	C3	C4	1.389(2)	C2	C3	1.377(5)	C2A	C3A	1.381(5)
C2	C3	1.391(3)	C4	C5	1.407(2)	C2	C7	1.399(5)	C2A	C7A	1.404(5)
C2	C7	1.385(3)	C5	C6	1.410(2)	C3	C4	1.396(5)	C3A	C4A	1.376(4)
C3	C4	1.379(3)	C6	C7	1.375(2)	C4	C5	1.395(6)	C4A	C5A	1.398(4)
C4	C5	1.400(3)	C8	C9	1.510(2)	C5	C6	1.394(6)	C5A	C6A	1.389(5)
C5	C6	1.405(3)	C10	C11	1.518(3)	C6	C7	1.376(5)	C6A	C7A	1.369(5)
C6	C7	1.377(3)	C12	C13	1.464(2)	C8	C9	1.533(7)	C8A	C9A	1.493(5)
C8	C9	1.517(4)	C13	C14	1.398(2)	C10	C11	1.518(7)	C10A	C11A	1.517(5)
C10	C11	1.512(4)	C13	C18	1.384(3)	C13	C14	1.393(5)	C13A	C14A	1.386(5)
C13	C14	1.399(4)	C14	C15	1.387(2)	C13	C18	1.382(5)	C13A	C18A	1.390(5)

C13	C18	1.378(4)	C15	C16	1.395(3)	C14	C15	1.389(7)	C14A	C15A	1.387(6)
C14	C15	1.391(4)	C16	C17	1.371(3)	C15	C16	1.367(8)	C15A	C16A	1.367(7)
C15	C16	1.372(5)	C17	C18	1.382(3)	C16	C17	1.361(6)	C16A	C17A	1.361(7)
C16	C17	1.384(5)				C17	C18	1.385(5)	C17A	C18A	1.385(6)
C17	C18	1.393(4)							O1M	C1M	1.443(19)
									O2M	C2M	1.43(3)
									O3M	C3M	1.47(3)

Table S3 Bond Angles for **12**, **15** and **23**.

**12**				**15**				**23**	**A**			**23**	**B**		
**Atom**	**Atom**	**Atom**	**Angle/°**	**Atom**	**Atom**	**Atom**	**Angle/°**	**Atom**	**Atom**	**Atom**	**Angle/°**	**Atom**	**Atom**	**Atom**	**Angle/°**
C9	O4	C10	109.3(3)	C10	O4	C9	109.91(15)	C10	O4	C9	110.2(4)	C10A	O4A	C9A	111.1(3)
C14	O5	C19	117.9(2)	C15	O5	C19	117.84(17)	C1	N1	N2	118.3(3)	C1A	N1A	N2A	119.0(3)
C1	N1	N2	118.9(2)	C1	N1	N2	118.81(13)	C12	N2	N1	116.3(3)	N1A	N2A	C12A	117.5(3)
C12	N2	N1	119.9(2)	C12	N2	N1	115.76(13)	O2	N3	C4	117.6(4)	O2A	N3A	O3A	122.8(4)
O2	N3	C4	116.8(2)	O2	N3	C4	118.39(14)	O3	N3	O2	123.0(4)	O2A	N3A	C4A	117.3(3)
O3	N3	O2	123.1(2)	O3	N3	O2	122.41(16)	O3	N3	C4	119.4(4)	O3A	N3A	C4A	119.9(4)
O3	N3	C4	120.0(2)	O3	N3	C4	119.19(15)	C5	N4	C8	120.3(4)	C5A	N4A	C8A	112.4(3)
C5	N4	C8	113.8(2)	C5	N4	C8	120.09(14)	C5	N4	C11	118.4(4)	C5A	N4A	C11A	115.2(3)
C5	N4	C11	115.5(2)	C5	N4	C11	119.69(14)	C11	N4	C8	111.3(4)	C11A	N4A	C8A	109.3(3)
C11	N4	C8	110.4(2)	C11	N4	C8	110.71(15)	C12	N5	C13	126.3(3)	C12A	N5A	C13A	128.0(3)
C12	N5	C13	127.6(2)	O1	C1	N1	123.03(15)	O1	C1	N1	120.8(3)	O1A	C1A	N1A	122.0(3)
O1	C1	N1	121.6(2)	O1	C1	C2	121.73(16)	O1	C1	C2	121.2(3)	O1A	C1A	C2A	121.5(3)
O1	C1	C2	122.9(2)	N1	C1	C2	115.19(14)	N1	C1	C2	117.9(3)	N1A	C1A	C2A	116.5(3)
N1	C1	C2	115.5(2)	C3	C2	C1	122.84(16)	C3	C2	C1	125.3(3)	C3A	C2A	C1A	123.9(3)
C3	C2	C1	119.3(2)	C7	C2	C1	118.73(15)	C3	C2	C7	118.3(3)	C3A	C2A	C7A	118.2(3)

12

Atom	Atom	Atom	Angle/°
C7	C2	C1	121.9(2)
C7	C2	C3	118.7(2)
C4	C3	C2	119.2(2)
C3	C4	N3	115.5(2)
C3	C4	C5	123.8(2)
C5	C4	N3	120.7(2)
C4	C5	N4	122.6(2)
C4	C5	C6	115.3(2)
C6	C5	N4	122.0(2)
C7	C6	C5	121.7(2)
C6	C7	C2	121.3(2)
N4	C8	C9	110.1(3)
O4	C9	C8	111.8(3)
O4	C10	C11	110.4(3)
N4	C11	C10	110.5(3)
N2	C12	S1	120.80(18)
N5	C12	S1	127.7(2)
N5	C12	N2	111.5(2)
C14	C13	N5	116.0(2)
C18	C13	N5	123.5(3)
C18	C13	C14	120.4(2)
O5	C14	C13	115.6(2)
O5	C14	C15	125.0(3)
C15	C14	C13	119.4(3)
C16	C15	C14	119.7(3)
C15	C16	C17	121.3(3)
C16	C17	C18	119.3(3)
C13	C18	C17	119.9(3)

15

Atom	Atom	Atom	Angle/°
C7	C2	C3	118.41(15)
C2	C3	C4	119.91(15)
C3	C4	N3	114.78(14)
C3	C4	C5	122.86(14)
C5	C4	N3	122.27(13)
N4	C5	C4	123.61(14)
N4	C5	C6	121.41(15)
C4	C5	C6	114.99(14)
C7	C6	C5	122.15(16)
C6	C7	C2	121.30(15)
N4	C8	C9	108.74(15)
O4	C9	C8	110.58(17)
O4	C10	C11	112.21(17)
N4	C11	C10	108.49(16)
N2	C12	C13	121.14(14)
C14	C13	C12	120.95(16)
C18	C13	C12	118.41(16)
C18	C13	C14	120.64(16)
C15	C14	C13	118.98(17)
O5	C15	C14	124.45(19)
O5	C15	C16	115.61(18)
C14	C15	C16	119.94(18)
C17	C16	C15	120.39(18)
C16	C17	C18	120.39(19)
C17	C18	C13	119.65(19)

23 A

Atom	Atom	Atom	Angle/°
C7	C2	C1	116.0(3)
C2	C3	C4	119.5(3)
C3	C4	N3	113.9(3)
C5	C4	N3	122.6(3)
C5	C4	C3	123.3(3)
N4	C5	C4	124.2(4)
N4	C5	C6	120.2(4)
C6	C5	C4	115.5(3)
C7	C6	C5	122.2(4)
C6	C7	C2	121.1(3)
N4	C8	C9	108.0(5)
O4	C9	C8	111.7(5)
O4	C10	C11	110.8(5)
N4	C11	C10	108.8(5)
O5	C12	N2	121.0(3)
O5	C12	N5	124.0(3)
N5	C12	N2	115.0(3)
C14	C13	N5	117.4(3)
C18	C13	N5	124.5(3)
C18	C13	C14	118.1(3)
C15	C14	C13	120.7(4)
C16	C15	C14	119.6(4)
C15	C16	Br1	120.6(3)
C17	C16	Br1	118.8(4)
C17	C16	C15	120.6(4)
C16	C17	C18	120.2(4)
C13	C18	C17	120.7(4)

23 B

Atom	Atom	Atom	Angle/°
C7A	C2A	C1A	117.9(3)
C4A	C3A	C2A	119.7(3)
C3A	C4A	N3A	116.4(3)
C3A	C4A	C5A	123.1(3)
C5A	C4A	N3A	120.4(3)
C4A	C5A	N4A	120.6(3)
C6A	C5A	N4A	123.4(3)
C6A	C5A	C4A	115.9(3)
C7A	C6A	C5A	122.0(3)
C6A	C7A	C2A	120.9(3)
N4A	C8A	C9A	110.0(4)
O4A	C9A	C8A	111.4(3)
O4A	C10A	C11A	111.0(4)
N4A	C11A	C10A	108.3(3)
O5A	C12A	N2A	121.6(3)
O5A	C12A	N5A	125.6(3)
N5A	C12A	N2A	112.8(3)
C14A	C13A	N5A	124.4(3)
C14A	C13A	C18A	119.7(3)
C18A	C13A	N5A	115.8(3)
C13A	C14A	C15A	119.3(4)
C16A	C15A	C14A	120.1(4)
C15A	C16A	Br1A	118.9(4)
C17A	C16A	Br1A	119.9(4)
C17A	C16A	C15A	121.2(4)
C16A	C17A	C18A	119.6(4)
C17A	C18A	C13A	120.0(4)

Atom	Atom	Atom	Angle/°
C10	O4	C9	109.91(15)
C15	O5	C19	117.84(17)
C1	N1	N2	118.81(13)
C12	N2	N1	115.76(13)
O2	N3	C4	118.39(14)
O3	N3	O2	122.41(16)
O3	N3	C4	119.19(15)
C5	N4	C8	120.09(14)
C5	N4	C11	119.69(14)
C11	N4	C8	110.71(15)
O1	C1	N1	123.03(15)
O1	C1	C2	121.73(16)
N1	C1	C2	115.19(14)
C3	C2	C1	122.84(16)
C7	C2	C1	118.73(15)
C7	C2	C3	118.41(15)
C2	C3	C4	119.91(15)
C3	C4	N3	114.78(14)
C3	C4	C5	122.86(14)
C5	C4	N3	122.27(13)
N4	C5	C4	123.61(14)
N4	C5	C6	121.41(15)
C4	C5	C6	114.99(14)
C7	C6	C5	122.15(16)

C6	C7	C2	121.30(15)
N4	C8	C9	108.74(15)
O4	C9	C8	110.58(17)
O4	C10	C11	112.21(17)
N4	C11	C10	108.49(16)
N2	C12	C13	121.14(14)
C14	C13	C12	120.95(16)
C18	C13	C12	118.41(16)
C18	C13	C14	120.64(16)
C15	C14	C13	118.98(17)
O5	C15	C14	124.45(19)
O5	C15	C16	115.61(18)
C14	C15	C16	119.94(18)
C17	C16	C15	120.39(18)
C16	C17	C18	120.39(19)
C17	C18	C13	119.65(19)

Table S4 Torsion Angles for **12**, **15** and **23**.

**12**					**15**					**23**	**A**				**23**	**B**			
**A**	**B**	**C**	**D**	**Angle/°**	**A**	**B**	**C**	**D**	**Angle/°**	**A**	**B**	**C**	**D**	**Angle/°**	**A**	**B**	**C**	**D**	**Angle/°**
O1	C1	C2	C3	26.7(4)	O1	C1	C2	C3	144.7(2)	Br1	C16	C17	C18	-178.9(3)	Br1A	C16A	C17A	C18A	-179.5(4)
O1	C1	C2	C7	-151.6(3)	O1	C1	C2	C7	-36.7(3)	O1	C1	C2	C3	174.3(3)	O1A	C1A	C2A	C3A	-170.7(3)
O2	N3	C4	C3	-48.9(4)	O2	N3	C4	C3	26.8(3)	O1	C1	C2	C7	-12.7(5)	O1A	C1A	C2A	C7A	7.2(5)
O2	N3	C4	C5	132.6(3)	O2	N3	C4	C5	-156.6(2)	O2	N3	C4	C3	-37.4(6)	O2A	N3A	C4A	C3A	-45.5(5)
O3	N3	C4	C3	131.0(3)	O3	N3	C4	C3	-152.06(17)	O2	N3	C4	C5	148.1(5)	O2A	N3A	C4A	C5A	131.6(4)
O3	N3	C4	C5	-47.5(4)	O3	N3	C4	C5	24.6(2)	O3	N3	C4	C3	139.8(4)	O3A	N3A	C4A	C3A	133.6(4)
O4	C10	C11	N4	-58.9(4)	O4	C10	C11	N4	56.9(2)	O3	N3	C4	C5	-34.8(6)	O3A	N3A	C4A	C5A	-49.3(6)
O5	C14	C15	C16	178.8(3)	O5	C15	C16	C17	-179.8(2)	O4	C10	C11	N4	-59.2(6)	O4A	C10A	C11A	N4A	59.8(5)

12

A	B	C	D	Angle/°
N1	N2	C12	S1	8.1(4)
N1	N2	C12	N5	-173.0(3)
N1	C1	C2	C3	-155.1(3)
N1	C1	C2	C7	26.6(4)
N2	N1	C1	O1	1.5(5)
N2	N1	C1	C2	-176.8(3)
N3	C4	C5	N4	-6.6(4)
N3	C4	C5	C6	175.8(3)
N4	C5	C6	C7	-176.1(3)
N4	C8	C9	O4	56.7(4)
N5	C13	C14	O5	-0.3(4)
N5	C13	C14	C15	179.3(3)
N5	C13	C18	C17	-178.8(3)
C1	N1	N2	C12	-179.3(3)
C1	C2	C3	C4	-179.0(3)
C1	C2	C7	C6	178.0(3)
C2	C3	C4	N3	-176.3(2)
C2	C3	C4	C5	2.2(4)
C3	C2	C7	C6	-0.3(4)
C3	C4	C5	N4	175.1(3)
C3	C4	C5	C6	-2.5(4)
C4	C5	C6	C7	1.5(4)
C5	N4	C8	C9	175.7(3)
C5	N4	C11	C10	-175.0(3)
C5	C6	C7	C2	-0.2(5)
C7	C2	C3	C4	-0.6(4)
C8	N4	C5	C4	-61.9(4)
C8	N4	C5	C6	115.5(3)
C8	N4	C11	C10	54.2(3)

15

A	B	C	D	Angle/°
N1	N2	C12	C13	179.29(15)
N1	C1	C2	C3	-37.8(2)
N1	C1	C2	C7	140.77(18)
N2	N1	C1	O1	0.4(3)
N2	N1	C1	C2	-177.09(15)
N2	C12	C13	C14	18.0(3)
N2	C12	C13	C18	-161.24(17)
N3	C4	C5	N4	10.6(2)
N3	C4	C5	C6	-169.57(15)
N4	C5	C6	C7	175.69(17)
N4	C8	C9	O4	-59.7(2)
C1	N1	N2	C12	-173.95(17)
C1	C2	C3	C4	176.86(16)
C1	C2	C7	C6	-174.27(17)
C2	C3	C4	N3	172.57(15)
C2	C3	C4	C5	-4.1(2)
C3	C2	C7	C6	4.4(3)
C3	C4	C5	N4	-172.98(16)
C3	C4	C5	C6	6.8(2)
C4	C5	C6	C7	-4.1(3)
C5	N4	C8	C9	-155.46(17)
C5	N4	C11	C10	157.32(17)
C5	C6	C7	C2	-1.3(3)
C7	C2	C3	C4	-1.7(2)
C8	N4	C5	C4	51.3(2)
C8	N4	C5	C6	-128.49(19)
C8	N4	C11	C10	-56.3(2)
C9	O4	C10	C11	-59.1(2)
C10	O4	C9	C8	60.1(2)

23 A

A	B	C	D	Angle/°
N1	N2	C12	O5	22.1(5)
N1	N2	C12	N5	-158.8(3)
N1	C1	C2	C3	-9.4(5)
N1	C1	C2	C7	163.6(3)
N2	N1	C1	O1	12.2(4)
N2	N1	C1	C2	-164.1(3)
N3	C4	C5	N4	-4.3(6)
N3	C4	C5	C6	173.4(4)
N4	C5	C6	C7	176.9(4)
N4	C8	C9	O4	56.8(6)
N5	C13	C14	C15	179.0(4)
N5	C13	C18	C17	-179.3(4)
C1	N1	N2	C12	66.1(4)
C1	C2	C3	C4	171.0(3)
C1	C2	C7	C6	-173.0(3)
C2	C3	C4	N3	-172.5(3)
C2	C3	C4	C5	2.0(6)
C3	C2	C7	C6	0.5(5)
C3	C4	C5	N4	-178.3(4)
C3	C4	C5	C6	-0.6(6)
C4	C5	C6	C7	-0.8(6)
C5	N4	C8	C9	158.7(4)
C5	N4	C11	C10	-156.4(4)
C5	C6	C7	C2	0.9(6)
C7	C2	C3	C4	-1.8(5)
C8	N4	C5	C4	-53.7(6)
C8	N4	C5	C6	128.7(5)
C8	N4	C11	C10	57.9(5)
C9	O4	C10	C11	60.3(6)

23 B

A	B	C	D	Angle/°
N1A	N2A	C12A	O5A	17.8(4)
N1A	N2A	C12A	N5A	-164.0(3)
N1A	C1A	C2A	C3A	8.3(5)
N1A	C1A	C2A	C7A	-173.8(4)
N2A	N1A	C1A	O1A	3.4(5)
N2A	N1A	C1A	C2A	-175.6(3)
N3A	C4A	C5A	N4A	3.7(6)
N3A	C4A	C5A	C6A	-174.9(4)
N4A	C5A	C6A	C7A	177.8(4)
N4A	C8A	C9A	O4A	-56.5(5)
N5A	C13A	C14A	C15A	-179.9(4)
N5A	C13A	C18A	C17A	-179.4(4)
C1A	N1A	N2A	C12A	78.9(4)
C1A	C2A	C3A	C4A	174.4(3)
C1A	C2A	C7A	C6A	-176.2(4)
C2A	C3A	C4A	N3A	178.6(3)
C2A	C3A	C4A	C5A	1.7(6)
C3A	C2A	C7A	C6A	1.9(7)
C3A	C4A	C5A	N4A	-179.4(3)
C3A	C4A	C5A	C6A	2.0(6)
C4A	C5A	C6A	C7A	-3.7(7)
C5A	N4A	C8A	C9A	-173.0(3)
C5A	N4A	C11A	C10A	173.8(3)
C5A	C6A	C7A	C2A	1.9(8)
C7A	C2A	C3A	C4A	-3.6(6)
C8A	N4A	C5A	C4A	94.8(4)
C8A	N4A	C5A	C6A	-86.7(5)
C8A	N4A	C11A	C10A	-58.6(4)
C9A	O4A	C10A	C11A	-59.1(5)

12

A	B	C	D	Angle/°
C9	O4	C10	C11	61.6(4)
C10	O4	C9	C8	-61.0(4)
C11	N4	C5	C4	168.9(3)
C11	N4	C5	C6	-13.7(4)
C11	N4	C8	C9	-52.6(4)
C12	N5	C13	C14	136.9(3)
C12	N5	C13	C18	-45.6(5)
C13	N5	C12	S1	6.8(5)
C13	N5	C12	N2	-172.0(3)
C13	C14	C15	C16	-0.7(5)
C14	C13	C18	C17	-1.4(5)
C14	C15	C16	C17	-0.7(6)
C15	C16	C17	C18	1.0(5)
C16	C17	C18	C13	0.0(5)
C18	C13	C14	O5	-177.8(3)
C18	C13	C14	C15	1.8(4)
C19	O5	C14	C13	-178.2(3)
C19	O5	C14	C15	2.2(5)

15

A	B	C	D	Angle/°
C11	N4	C5	C4	-165.48(17)
C11	N4	C5	C6	14.7(3)
C11	N4	C8	C9	58.3(2)
C12	C13	C14	C15	-178.81(17)
C12	C13	C18	C17	178.35(18)
C13	C14	C15	O5	-179.8(2)
C13	C14	C15	C16	-0.3(3)
C14	C13	C18	C17	-0.9(3)
C14	C15	C16	C17	0.6(3)
C15	C16	C17	C18	-1.1(4)
C16	C17	C18	C13	1.3(3)
C18	C13	C14	C15	0.4(3)
C19	O5	C15	C14	0.2(4)
C19	O5	C15	C16	-179.4(3)

23 A

A	B	C	D	Angle/°
C10	O4	C9	C8	-59.6(6)
C11	N4	C5	C4	163.8(4)
C11	N4	C5	C6	-13.8(6)
C11	N4	C8	C9	-56.3(6)
C12	N5	C13	C14	-171.0(4)
C12	N5	C13	C18	7.3(6)
C13	N5	C12	O5	-2.9(6)
C13	N5	C12	N2	178.1(3)
C13	C14	C15	C16	0.5(7)
C14	C13	C18	C17	-0.9(6)
C14	C15	C16	Br1	178.5(4)
C14	C15	C16	C17	-1.2(7)
C15	C16	C17	C18	0.8(7)
C16	C17	C18	C13	0.3(7)
C18	C13	C14	C15	0.6(7)

23 B

A	B	C	D	Angle/°
C10A	O4A	C9A	C8A	57.4(5)
C11A	N4A	C5A	C4A	-139.1(4)
C11A	N4A	C5A	C6A	39.4(6)
C11A	N4A	C8A	C9A	57.8(4)
C12A	N5A	C13A	C14A	-2.7(5)
C12A	N5A	C13A	C18A	177.9(3)
C13A	N5A	C12A	O5A	9.1(5)
C13A	N5A	C12A	N2A	-169.0(3)
C13A	C14A	C15A	C16A	-0.3(7)
C14A	C13A	C18A	C17A	1.1(6)
C14A	C15A	C16A	Br1A	-179.9(4)
C14A	C15A	C16A	C17A	0.6(8)
C15A	C16A	C17A	C18A	0.0(8)
C16A	C17A	C18A	C13A	-0.8(7)
C18A	C13A	C14A	C15A	-0.5(6)

Table S5. Hydrogen bonding geometry in **12**, **15** and **23**.

Crystal	D-H…A	D-H (Å)	H...A (Å)	D...A (Å)	D-H...A (°)	Symmetry operation
12	N2-H2…O1	0.86	1.998	2.782	151.1	1-x, 1-y, 2-z
12	N5-H5…O1	0.86	2.146	2.943	153.8	1-x, 1-y, 2-z
15	N1-H1…O1	0.87	2.313	3.087(2)	148.9	x, 1/2-y, -1/2+z
15	N1-H1…N2	0.87	2.551	3.290(2)	143.8	x, 1/2-y, -1/2+z
23	N5-H5…O5A	0.86	2.015	2.851(4)	163.6	-

23	N5A-H5A…O5	0.86	1.959	2.813(4)	171.9	1-x, -1/2+y, 3/2-z
23	N1A-H1A…O1	0.86	2.014	2.844(4)	162.1	-
23	N1-H1…O1A	0.86	2.069	2.906(4)	164.3	1-x, 1/2+y, 3/2-z
23	N2-H2…O4A	0.86	2.227	2.868(4)	131.3	-1+x, y, z
23	N2A-H2A…O4	0.86	2.325	2.965(4)	131.5	1-x, 1-y, 1-z

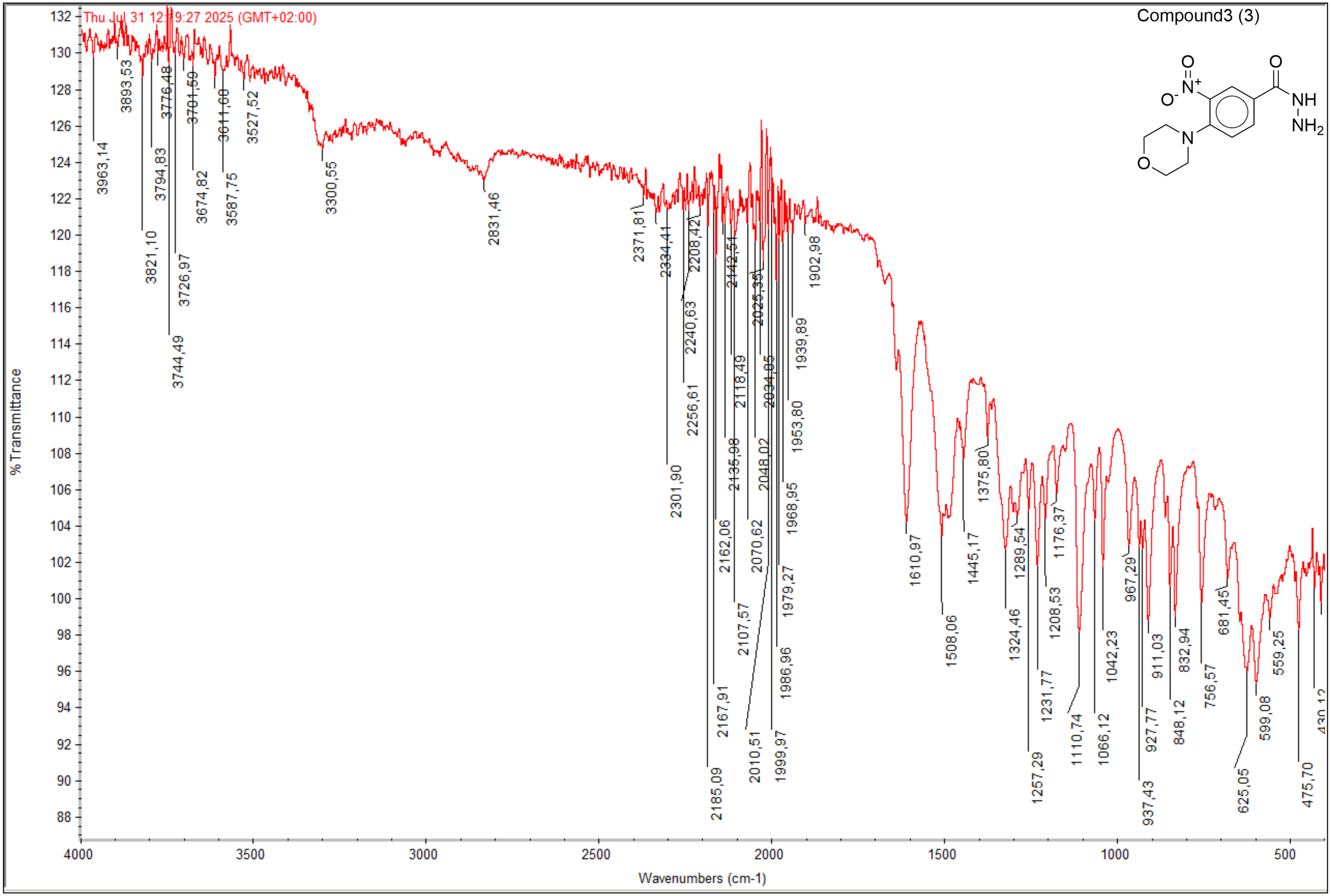

Figure S52. The IR spectra of compound **3**

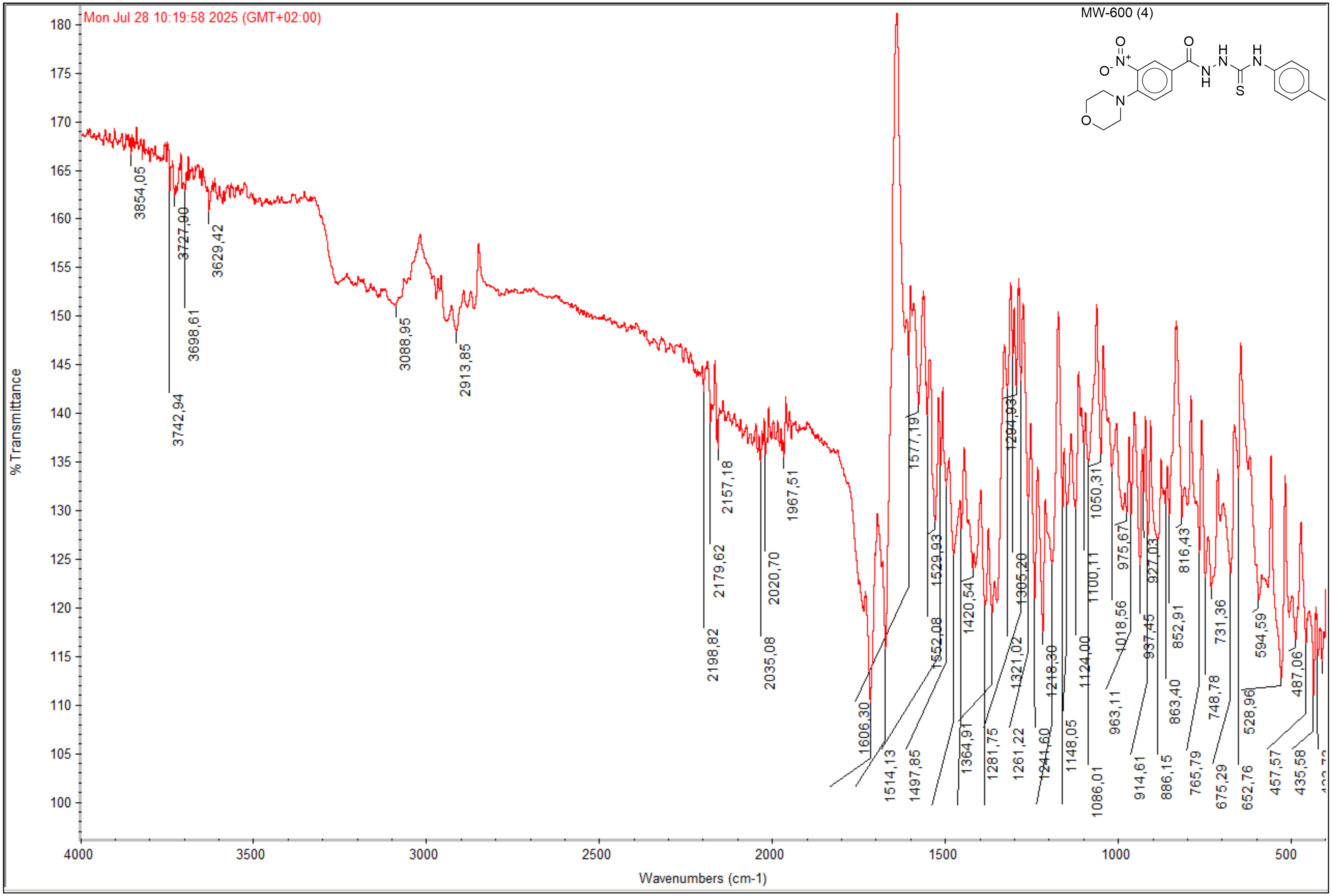

Figure **S53.** The IR spectra of compound **4**

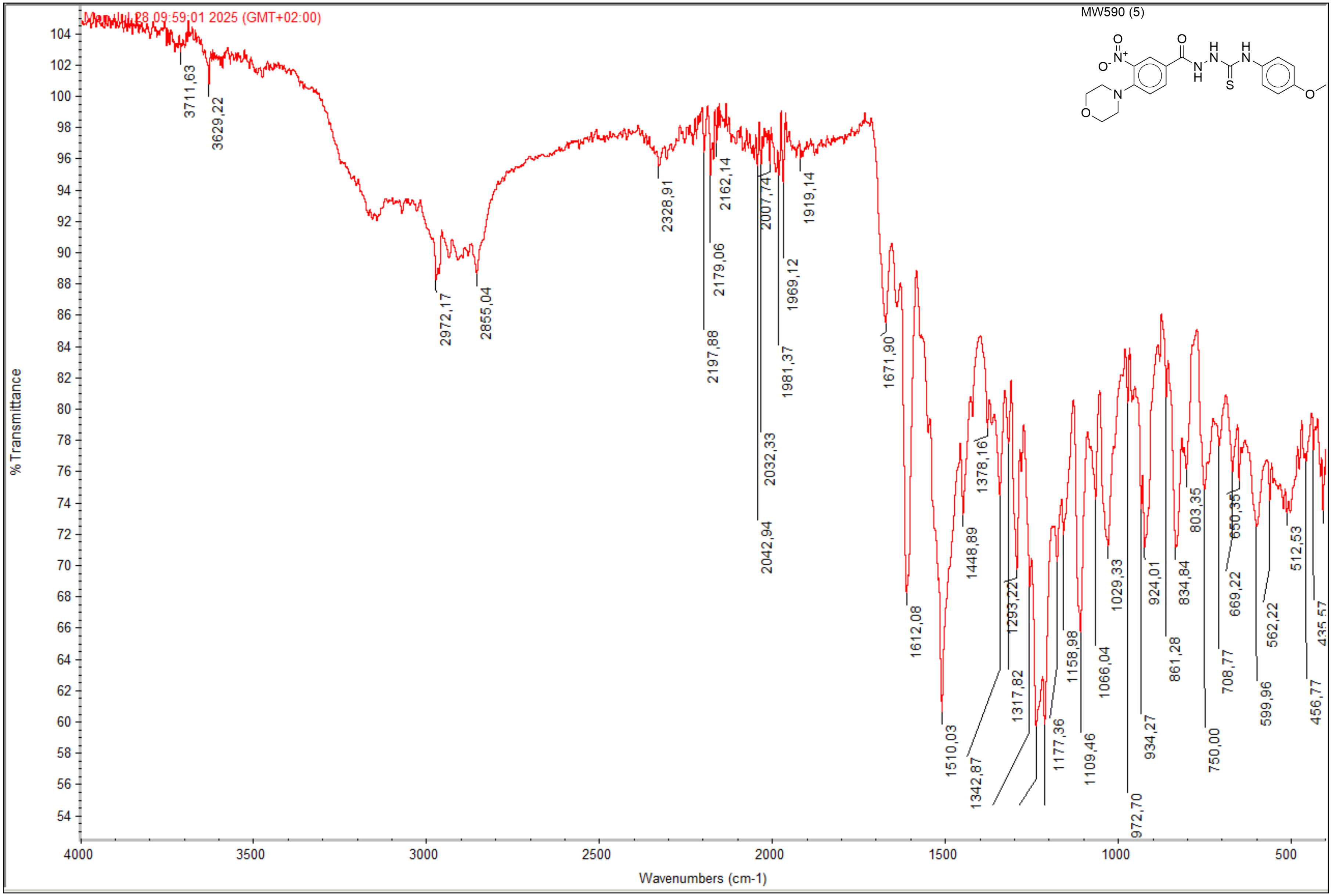

Figure **S54**. The IR spectra of compound **5**

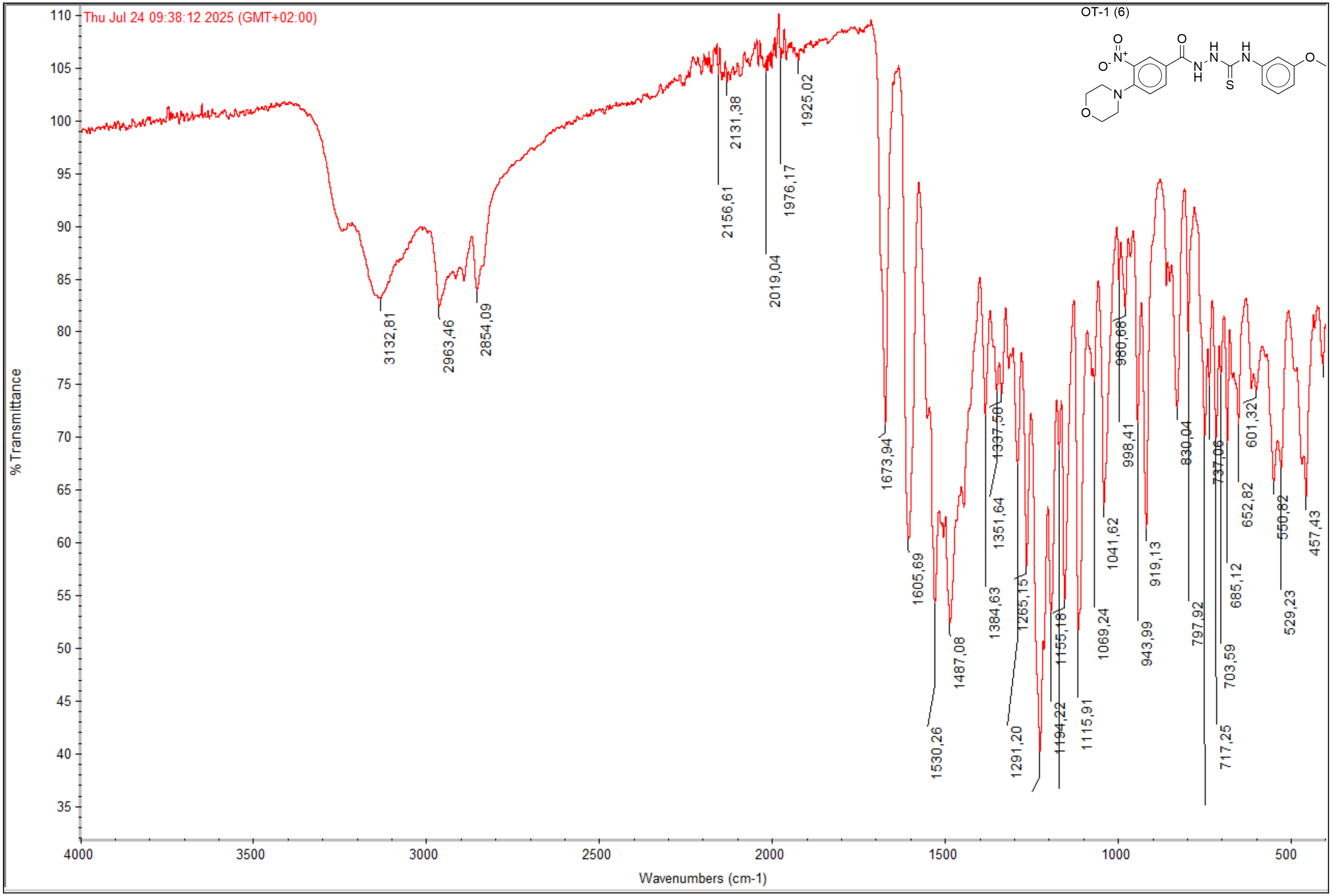

Figure **S55**. The IR spectra of compound **6**

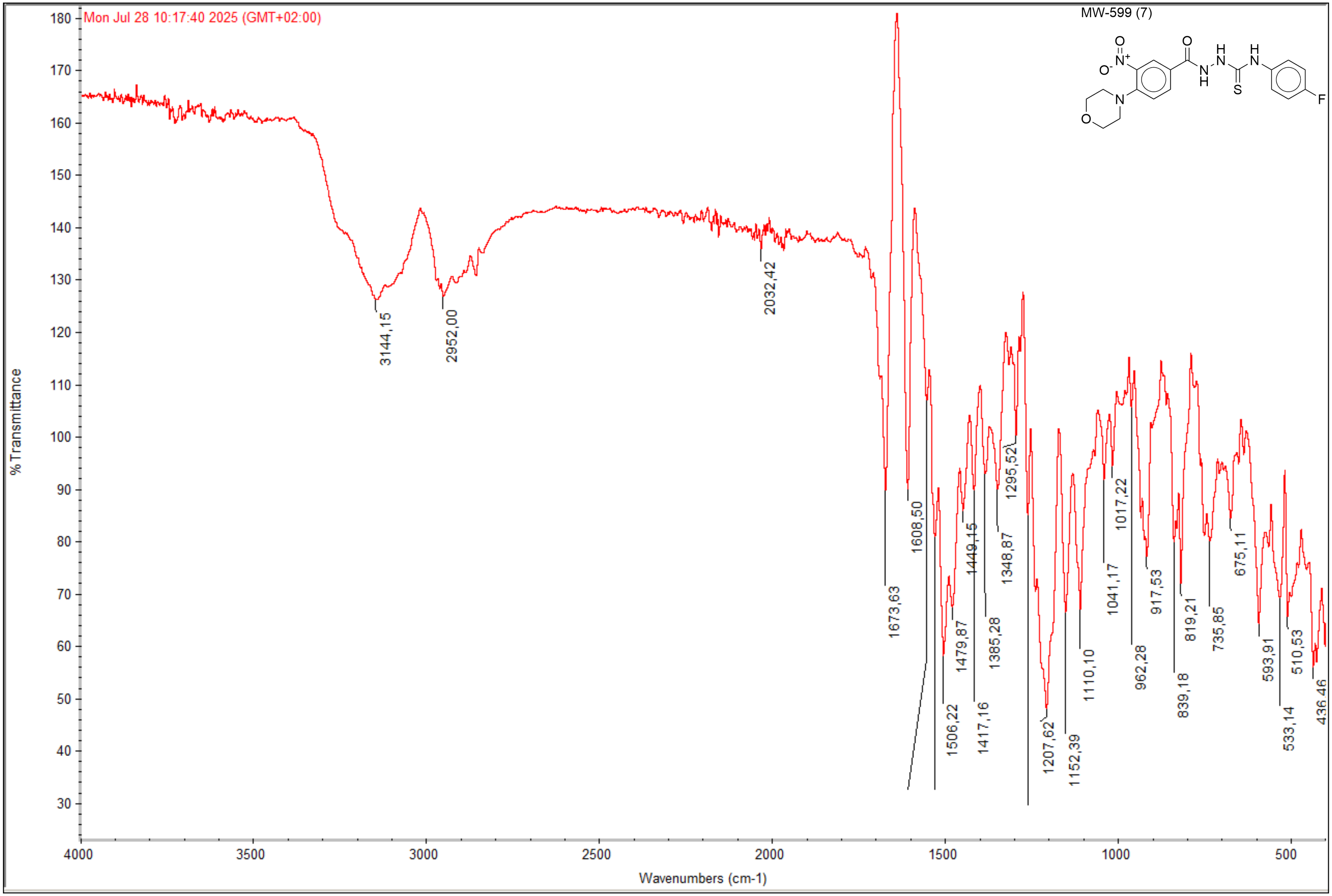

Figure **S56**. The IR spectra of compound **7**

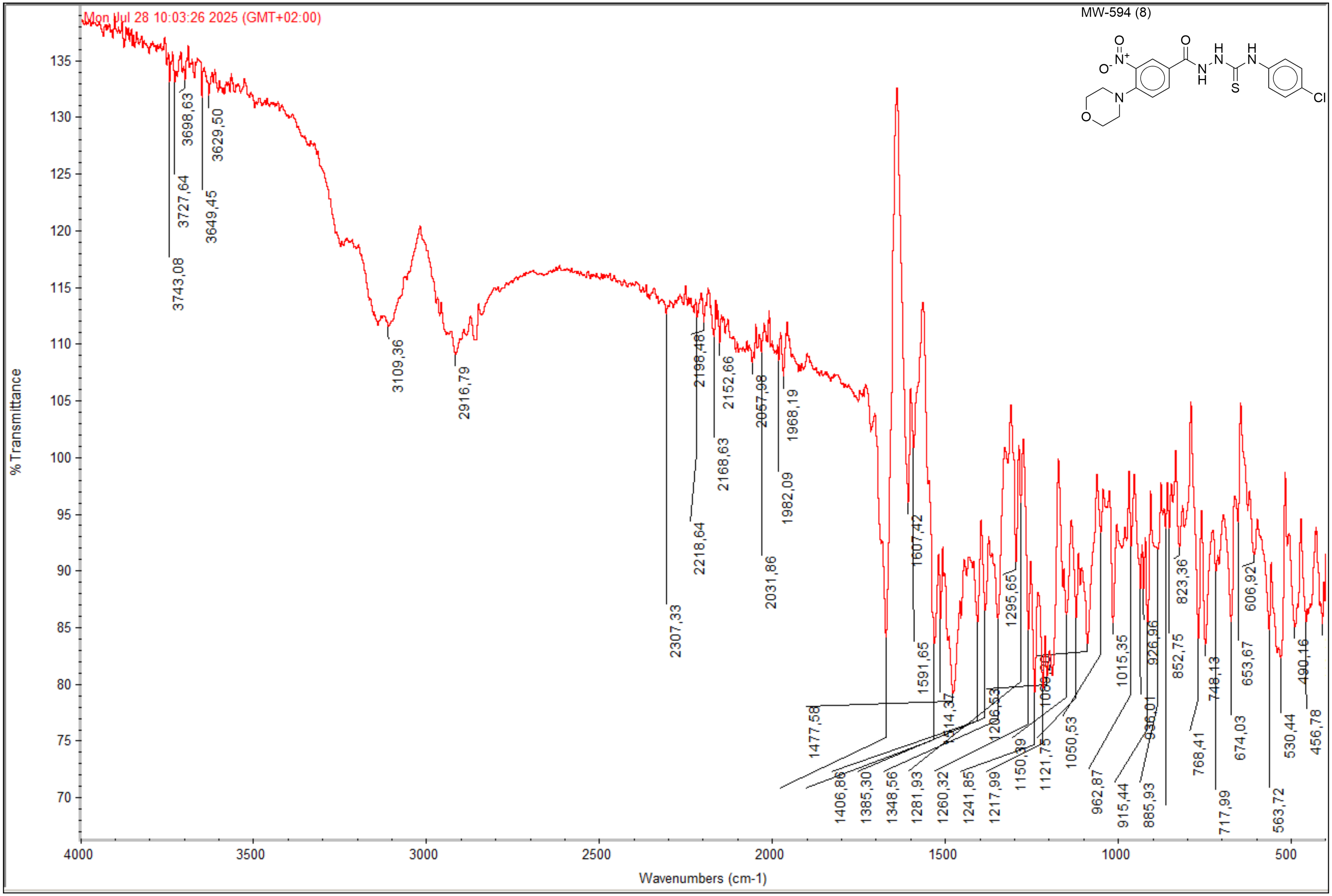

Figure **S57**. The IR spectra of compound **8**

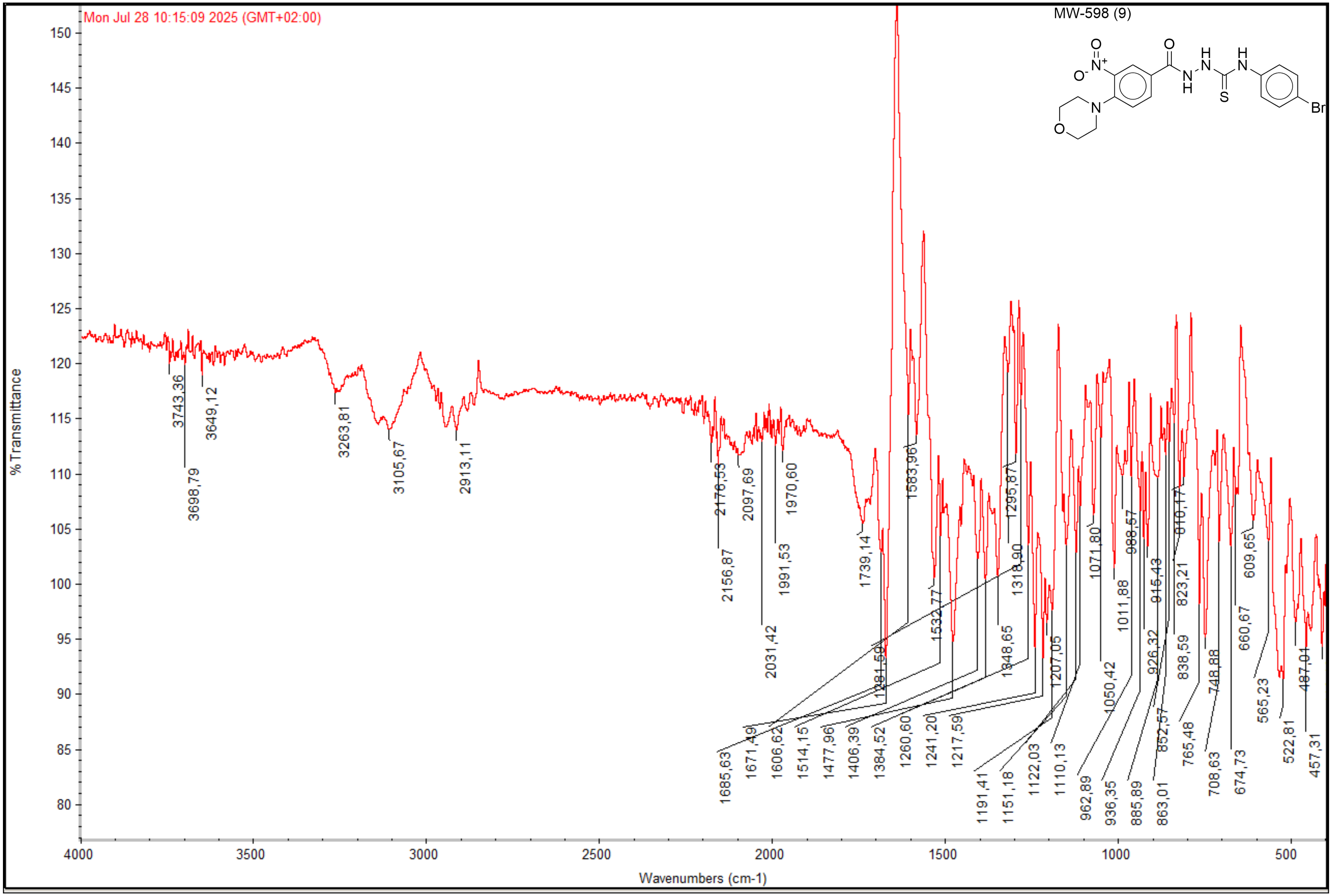

Figure **S58**. The IR spectra of compound **9**

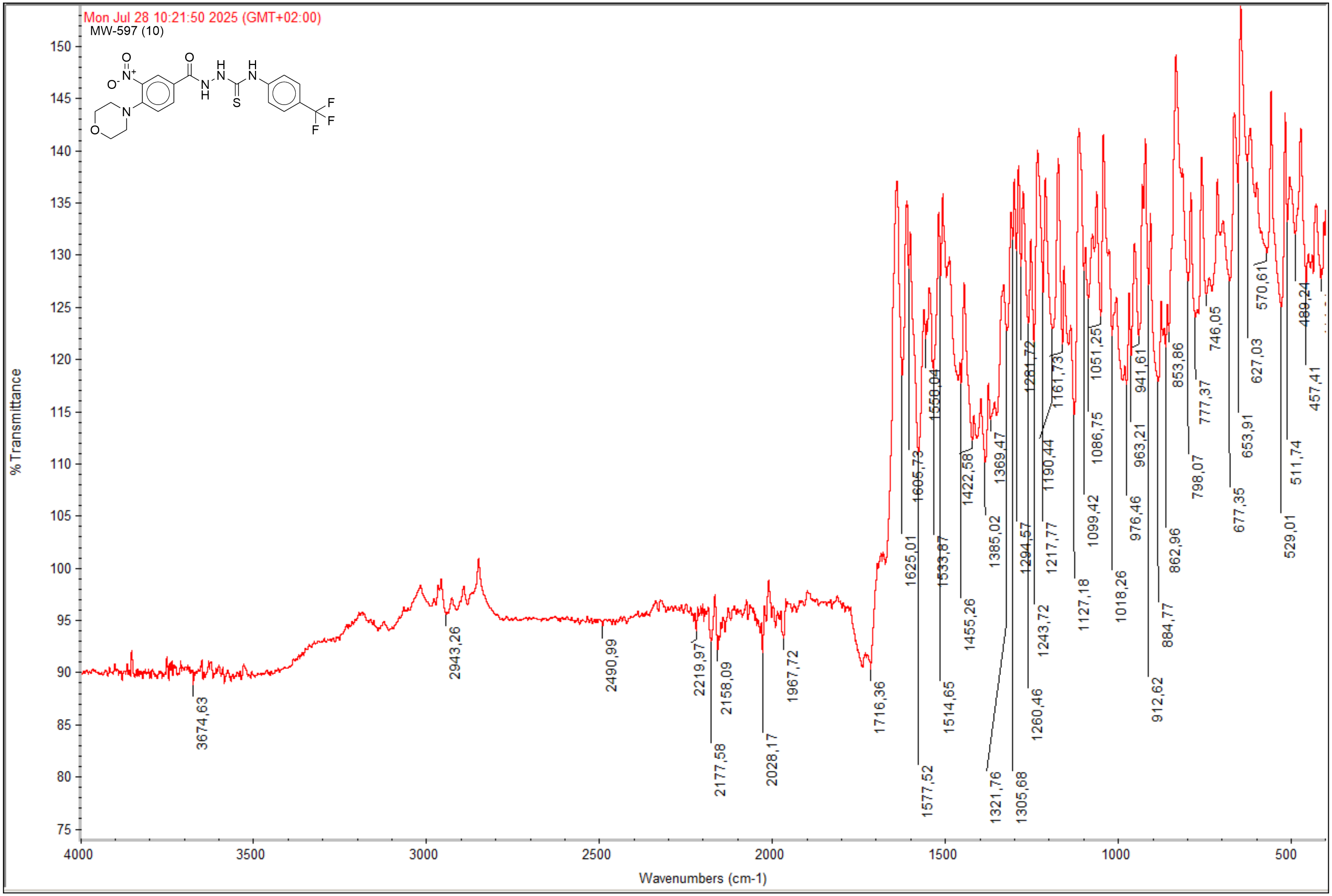

Figure **S59**. The IR spectra of compound **10**

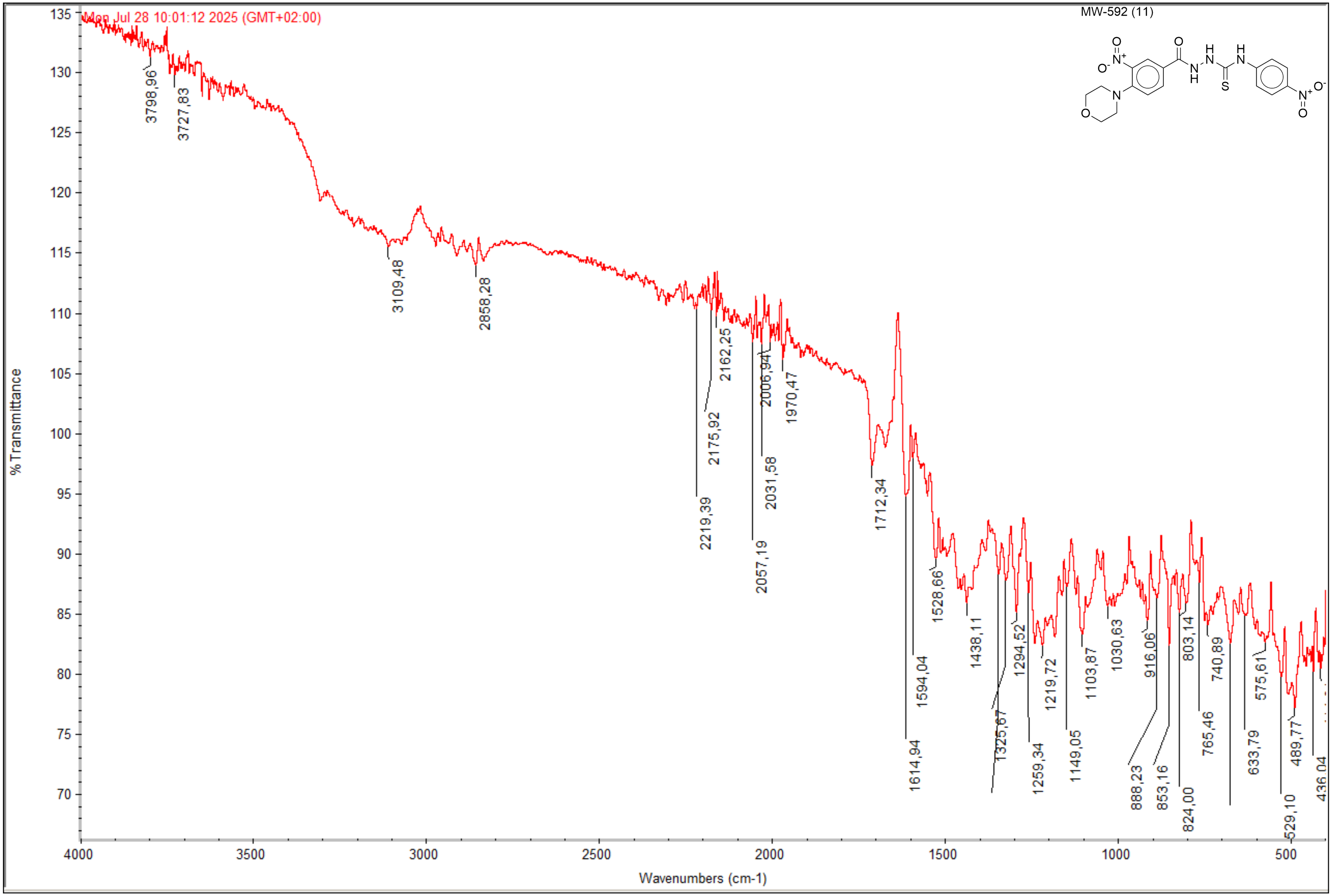

Figure **S60**. The IR spectra of compound **11**

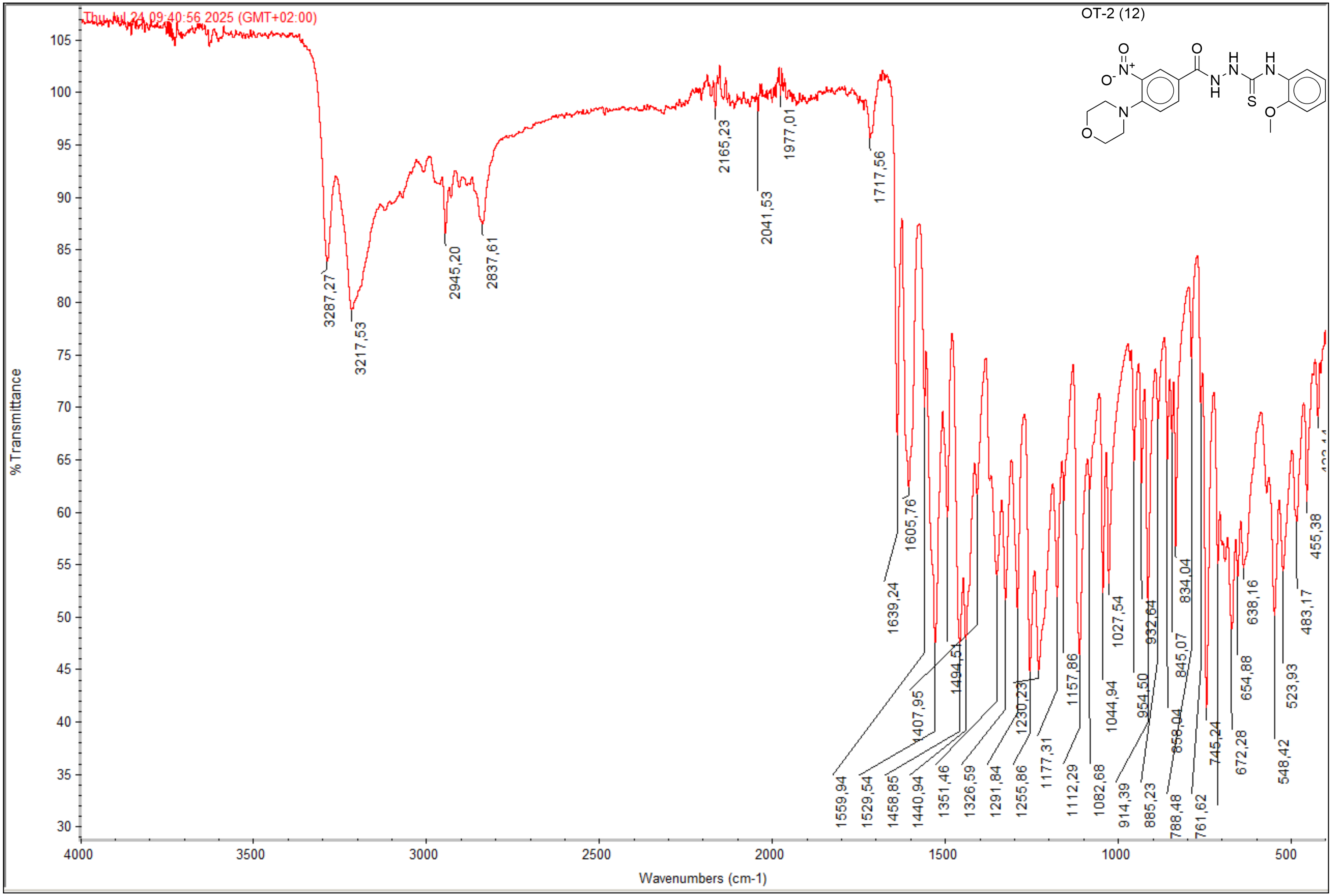

Figure **S61.** The IR spectra of compound **12**

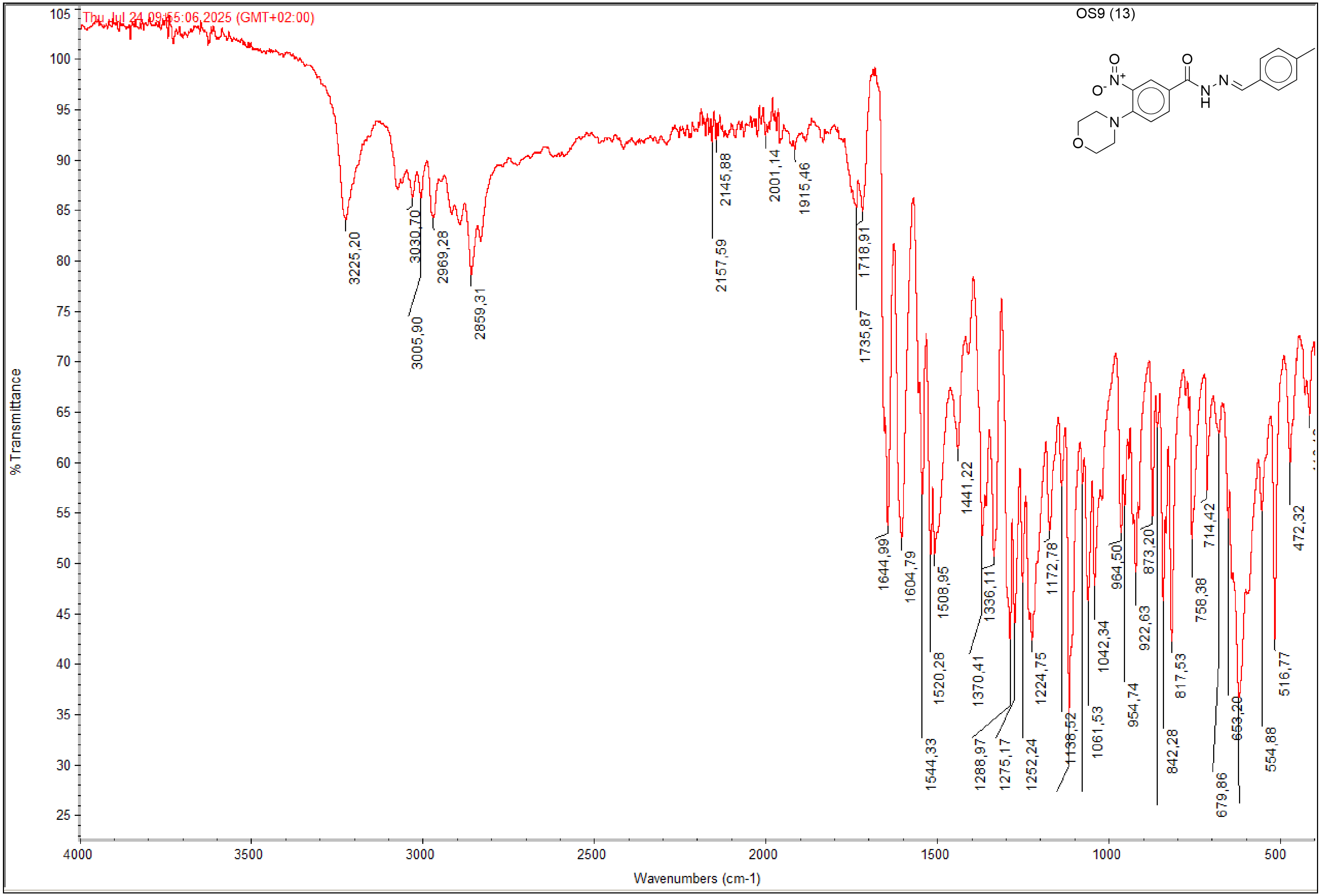

Figure S62. The IR spectra of compound **13**

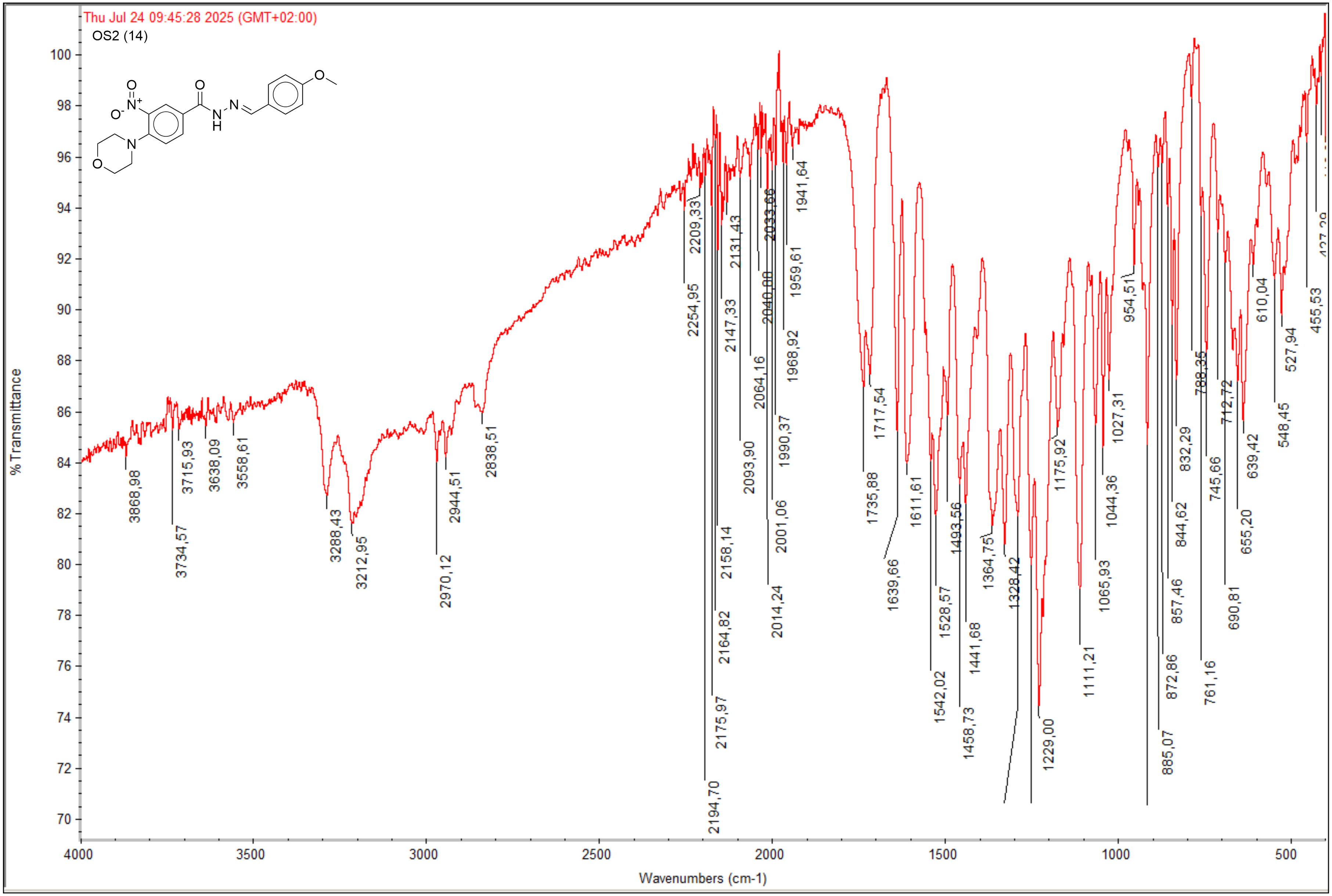

Figure S63. The IR spectra of compound **14**

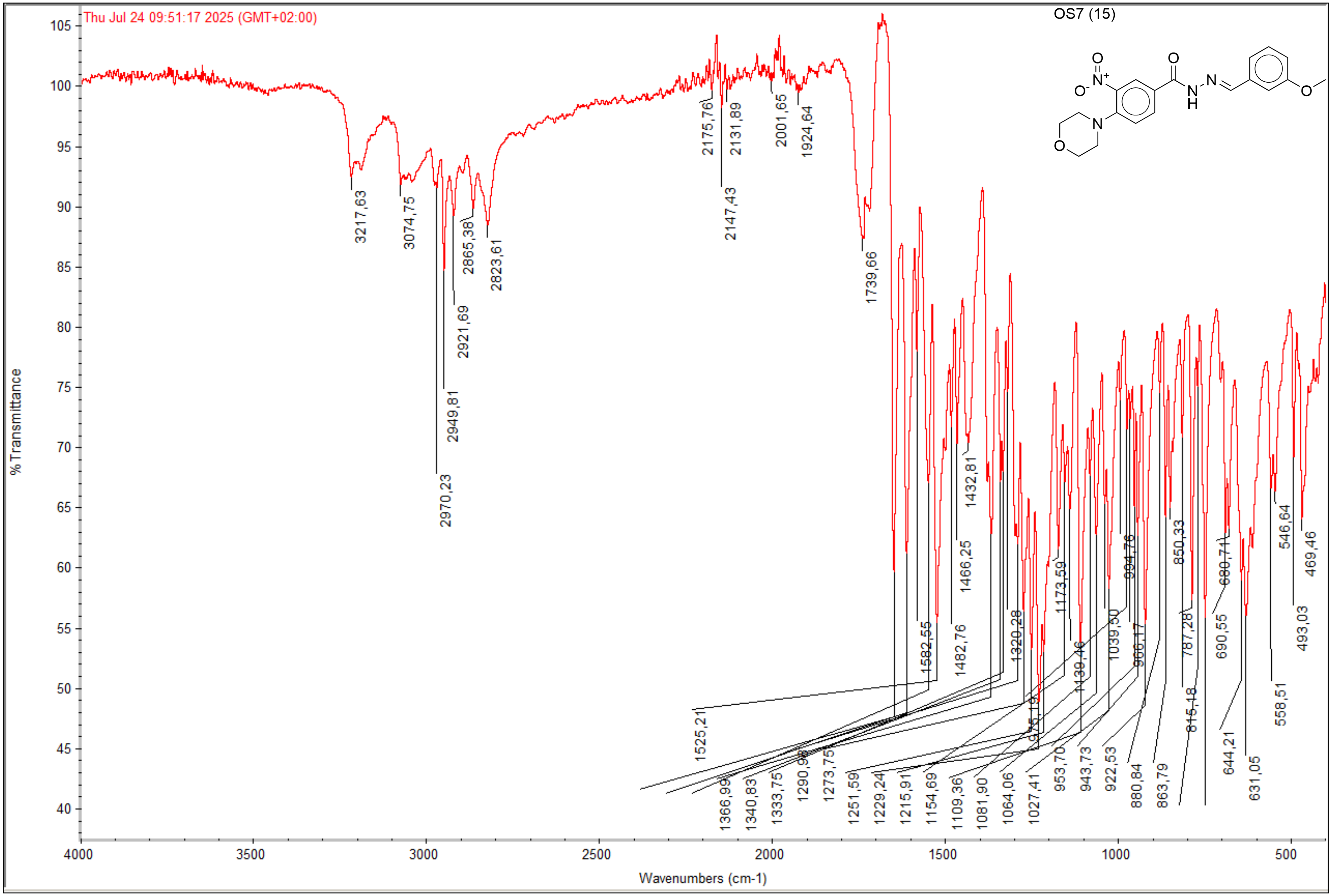

Figure S64. The IR spectra of compound **15**

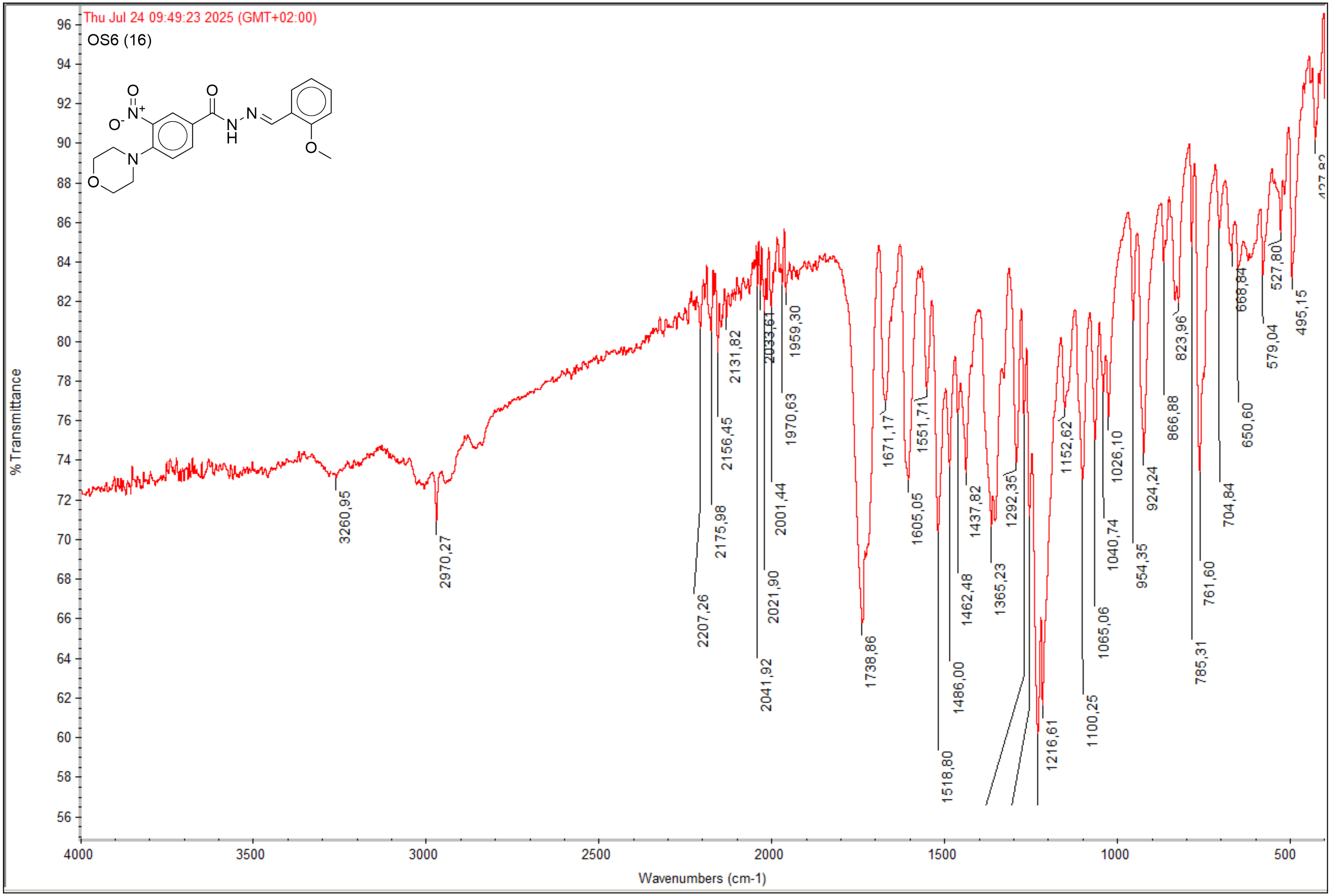

Figure S65. The IR spectra of compound **16**

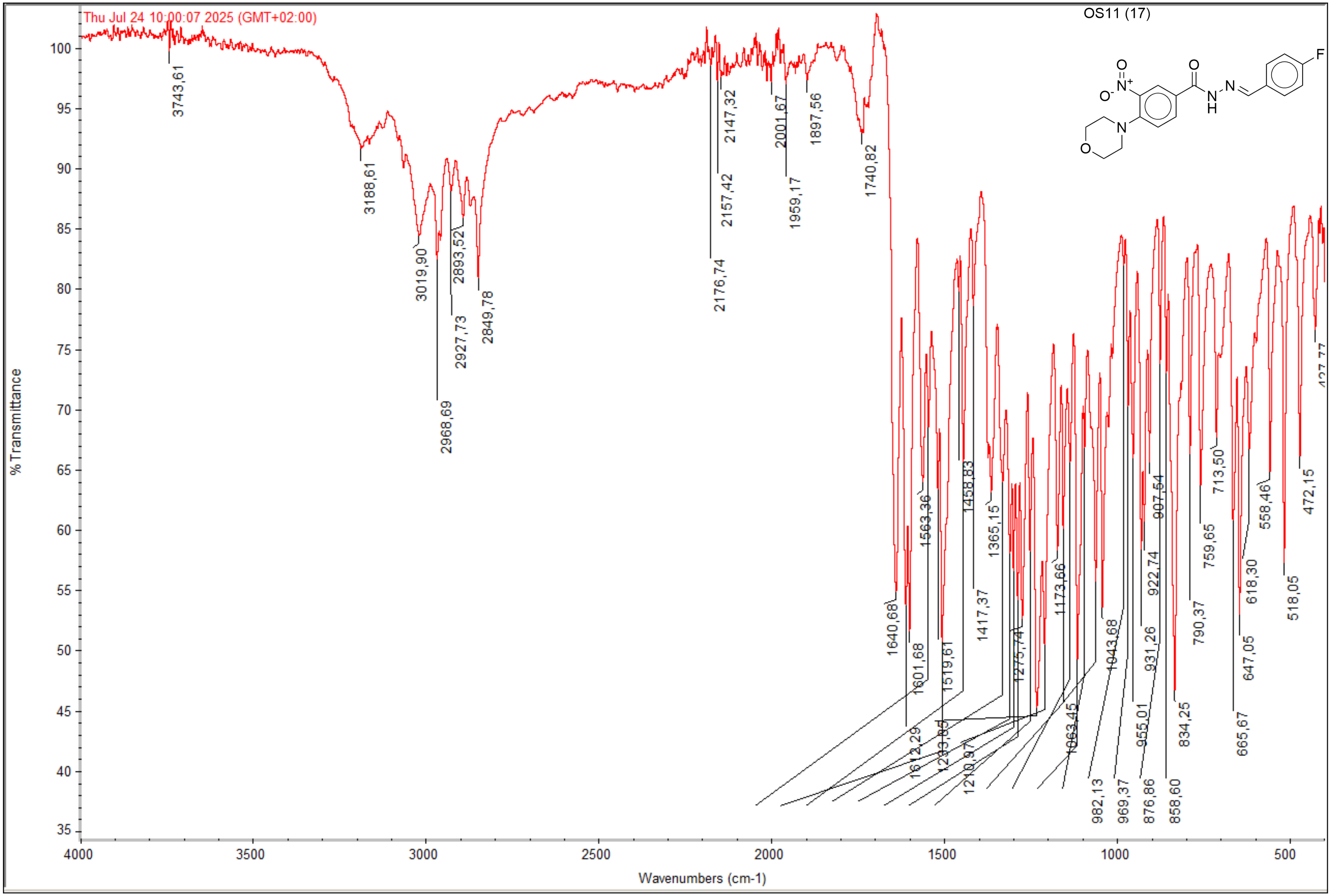

Figure S66. The IR spectra of compound **17**

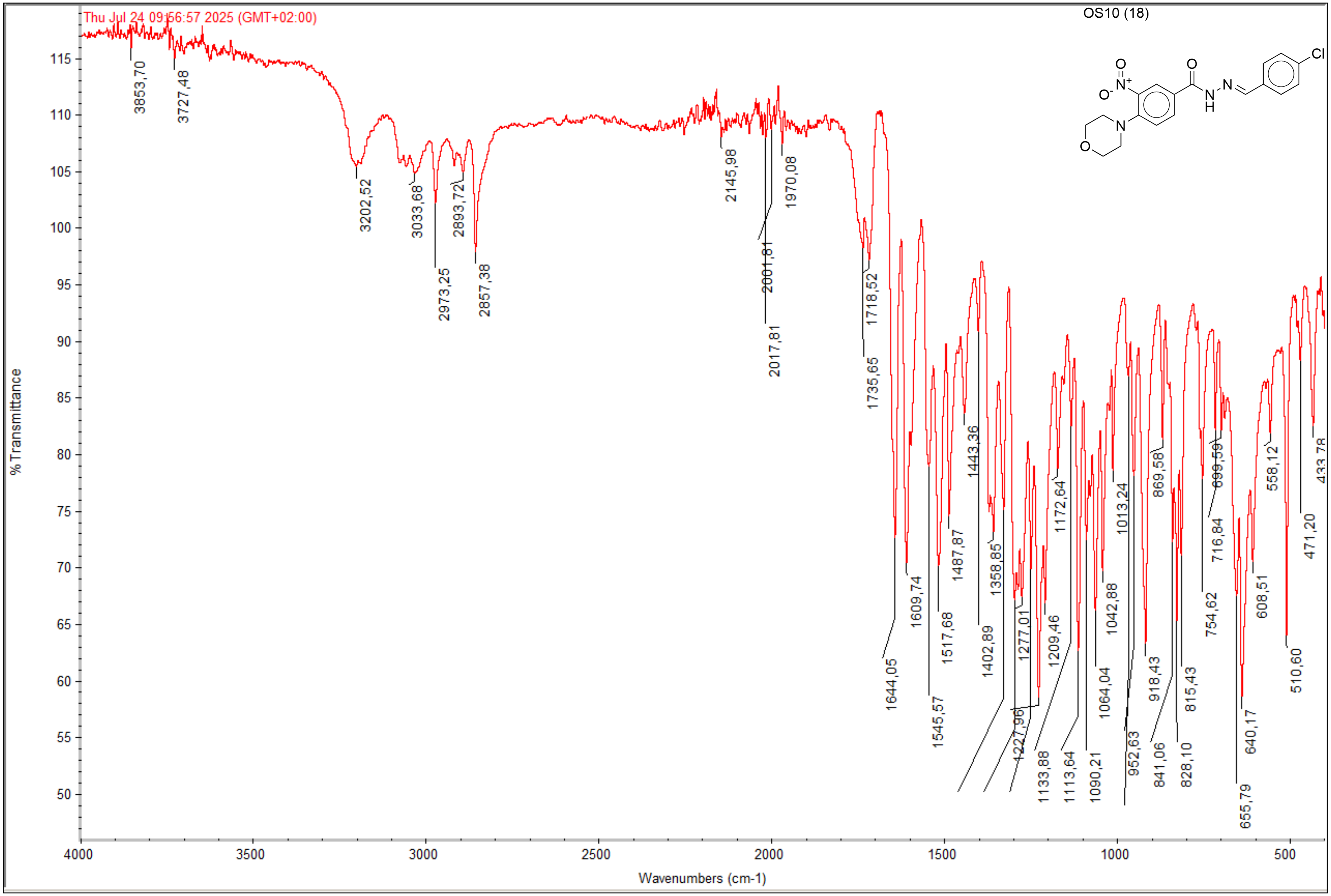

Figure S67. The IR spectra of compound **18**

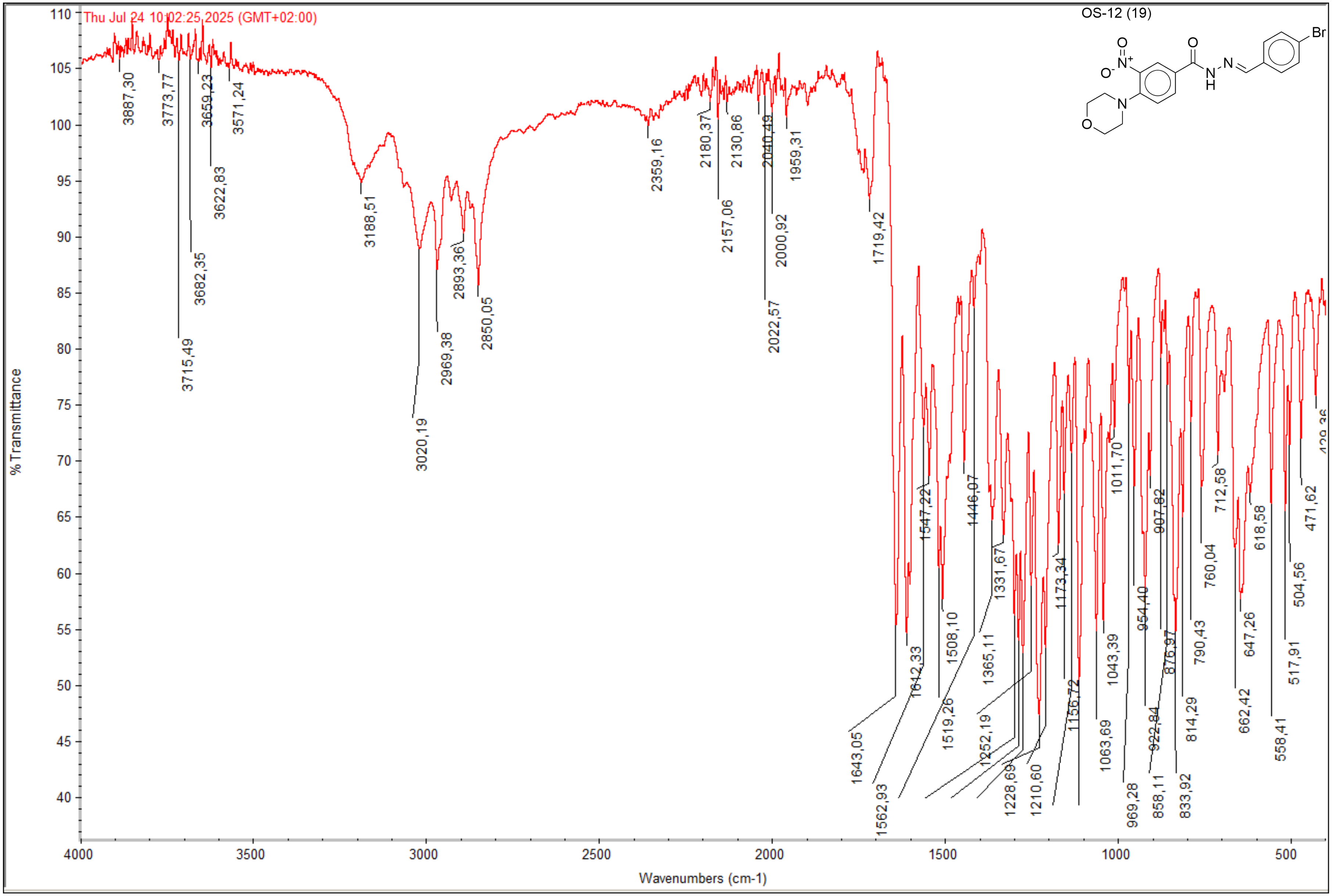

Figure S68. The IR spectra of compound **19**

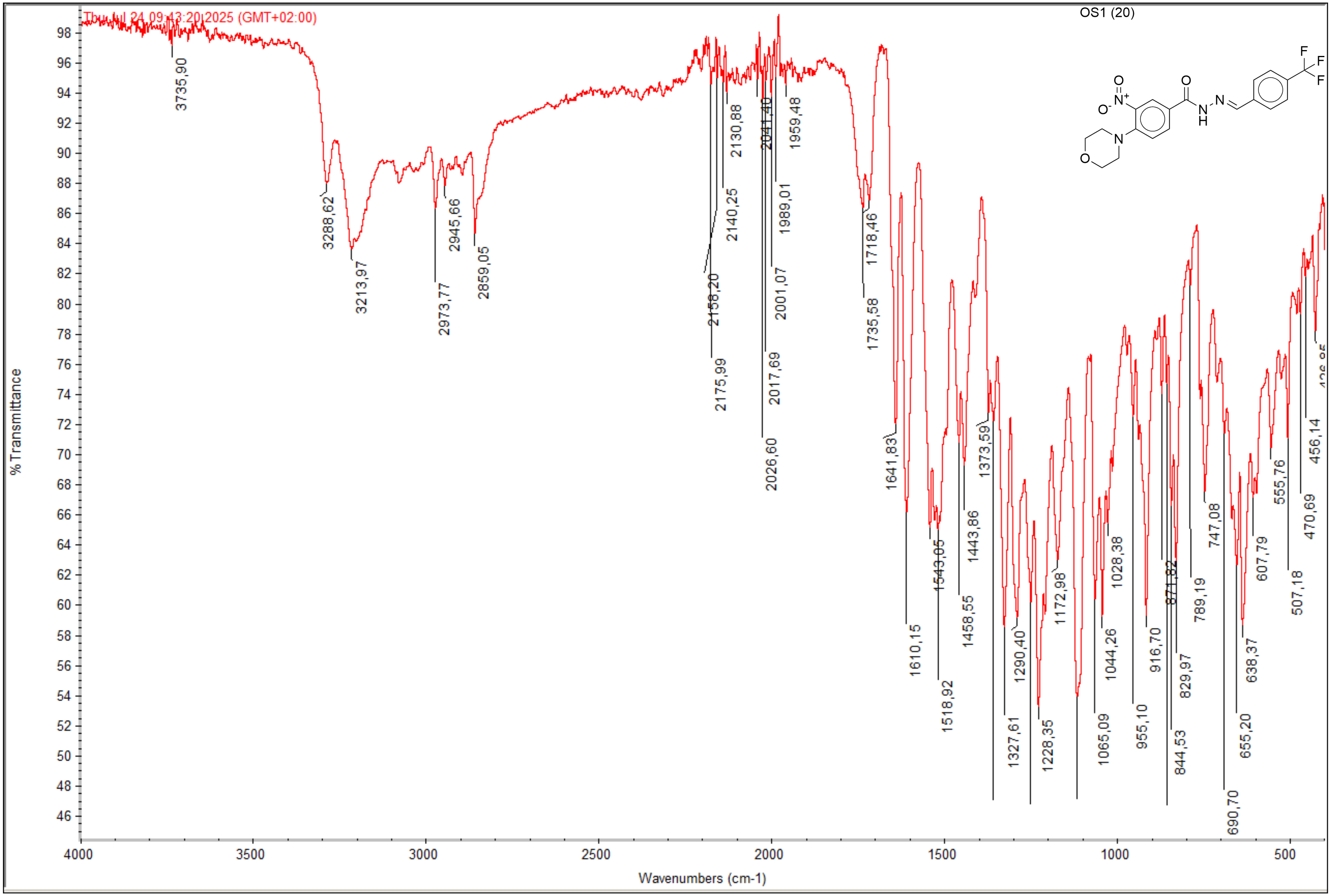

Figure S69. The IR spectra of compound **20**

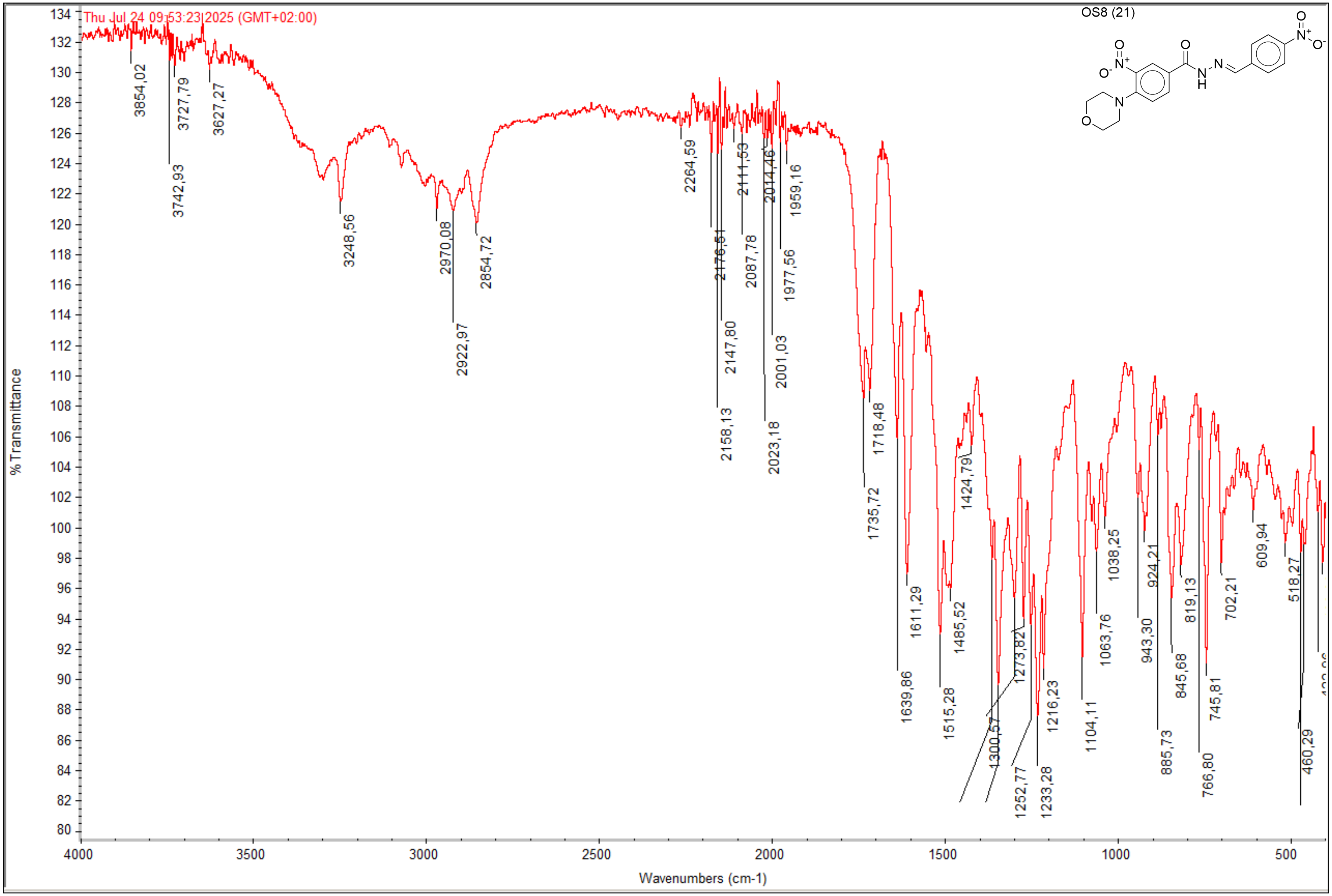

Figure **S70.** The IR spectra of compound **21**

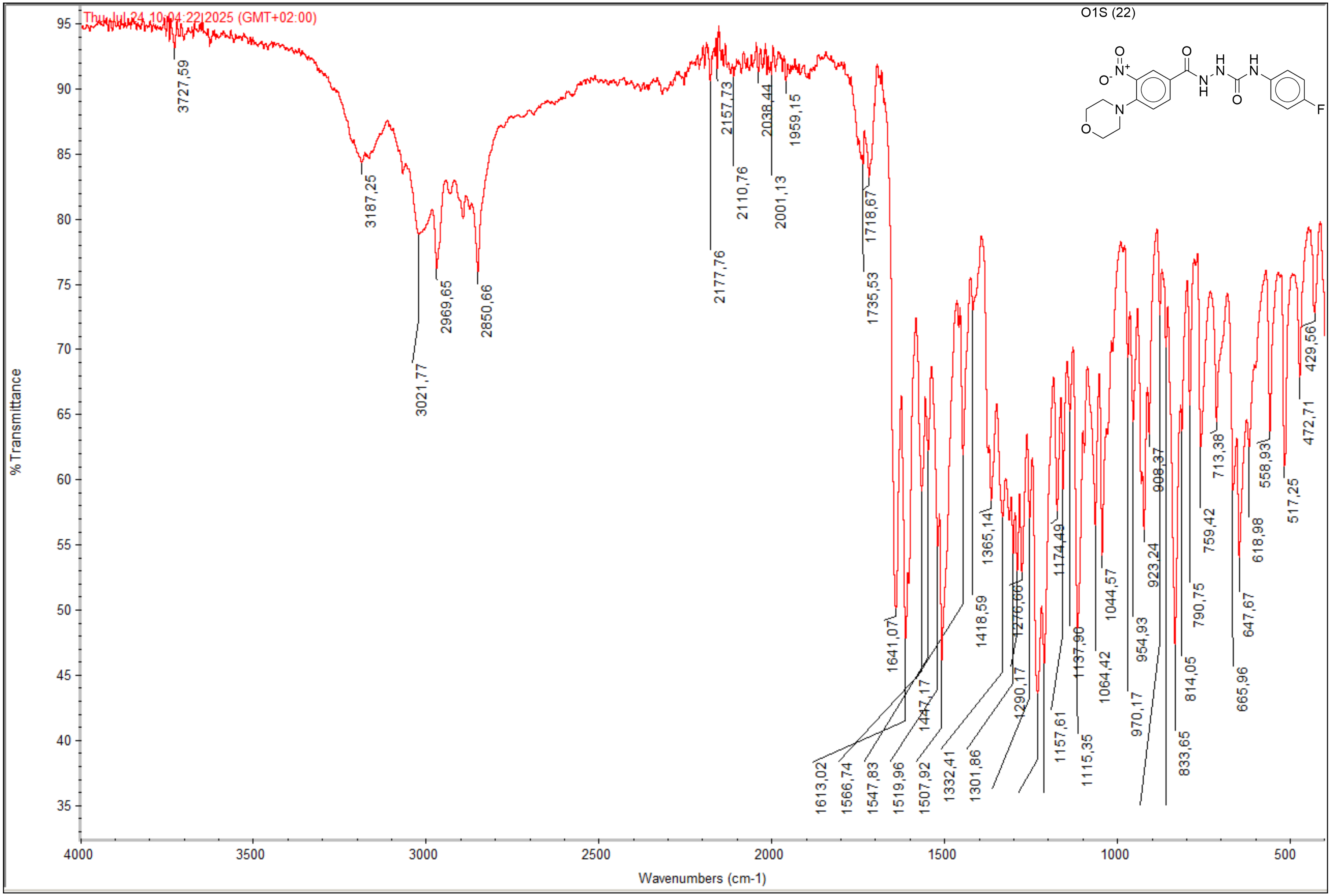

Figure S71. The IR spectra of compound **22**

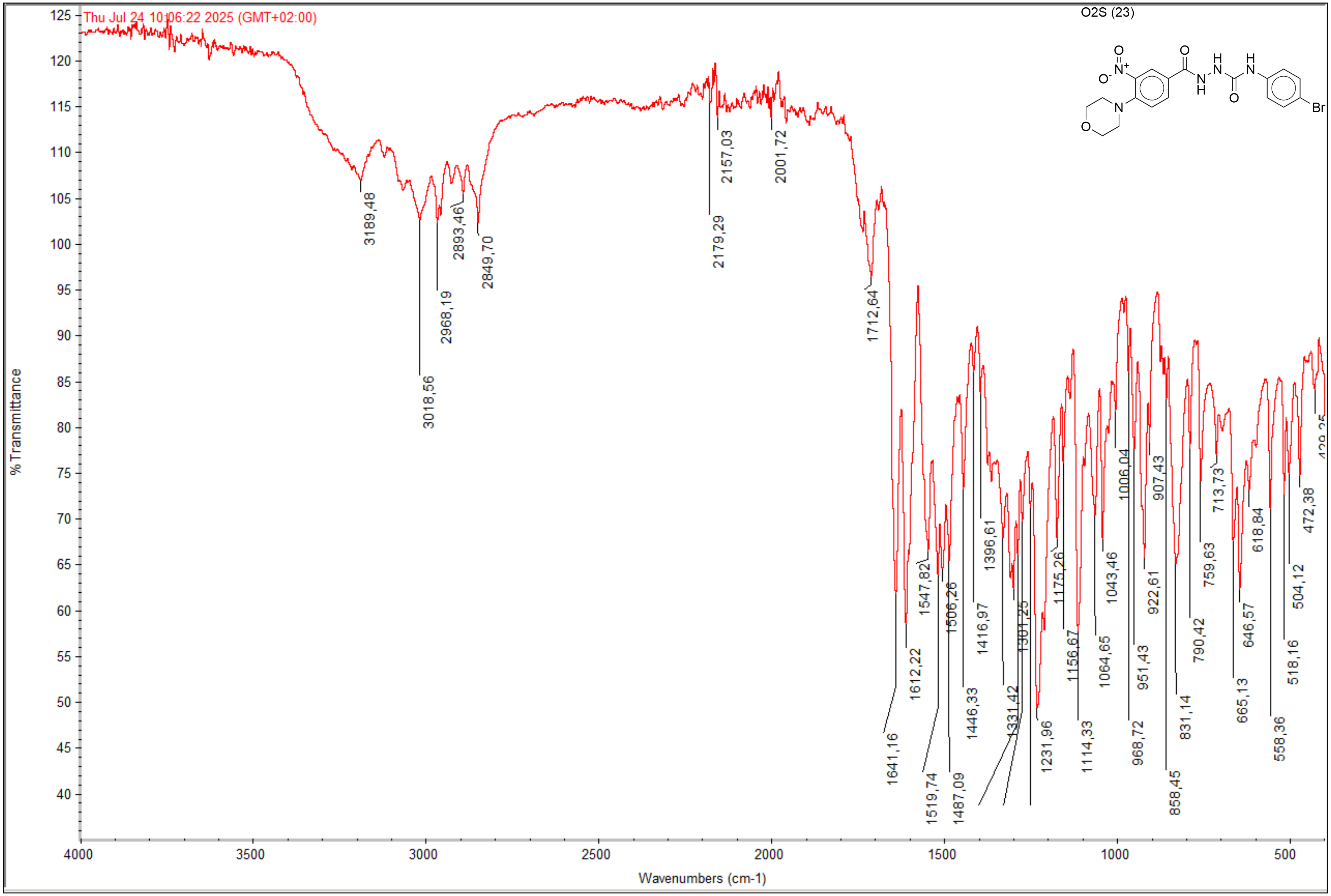

Figure S72. The IR spectra of compound **23**
